# Isogenic Pairs of hiPSC-CMs with Hypertrophic Cardiomyopathy/LVNC-Associated ACTC1 E99K Mutation Unveil Differential Functional Deficits

**DOI:** 10.1016/j.stemcr.2018.10.006

**Published:** 2018-11-01

**Authors:** James G.W. Smith, Thomas Owen, Jamie R. Bhagwan, Diogo Mosqueira, Elizabeth Scott, Ingra Mannhardt, Asha Patel, Roberto Barriales-Villa, Lorenzo Monserrat, Arne Hansen, Thomas Eschenhagen, Sian E. Harding, Steve Marston, Chris Denning

**Affiliations:** 1 Wolfson Centre for Stem Cells, Tissue Engineering and Modelling, Centre for Biomolecular Sciences, University of Nottingham, University Park, Nottingham NG7 2RD, UK; 2 National Heart and Lung Institute, Imperial College, London W12 0NN, UK; 3 Institute of Experimental Pharmacology and Toxicology, University Medical Centre, Hamburg-Eppendorf, Hamburg, Germany; 4 Inherited Cardiovascular Diseases Unit, Cardiology Service, Complexo Hospitalario Universitario A Coruña, Servizo Galego de Saúde (SERGAS), Universidade da Coruña, A Coruña, Spain; 5 Instituto de Investigación Biomédica de A Coruña (INIBIC), A Coruña, Spain; 6 Department of Gene Therapy, National Heart and Lung Institute, Imperial College London SW3 6LR, UK; 7 Faculty of Medicine and Health Sciences, Norwich Medical School, University of East Anglia, Norwich Research Park, Norwich NR4 7UQ, UK; 8 DZHK (German Centre for Cardiovascular Research), Partner Site Hamburg/Kiel/Lübeck, Hamburg, Germany; 9 Health in Code S.L., Cardiology Department, A Coruña, Spain

**Keywords:** arrhythmia, contractile function, hypertrophy, cardiomyopathy

## Abstract

Hypertrophic cardiomyopathy (HCM) is a primary disorder of contractility in heart muscle. To gain mechanistic insight and guide pharmacological rescue, this study models HCM using isogenic pairs of human induced pluripotent stem cell-derived cardiomyocytes (hiPSC-CMs) carrying the E99K-ACTC1 cardiac actin mutation. In both 3D engineered heart tissues and 2D monolayers, arrhythmogenesis was evident in all E99K-ACTC1 hiPSC-CMs. Aberrant phenotypes were most common in hiPSC-CMs produced from the heterozygote father. Unexpectedly, pathological phenotypes were less evident in E99K-expressing hiPSC-CMs from the two sons. Mechanistic insight from Ca^2+^ handling expression studies prompted pharmacological rescue experiments, wherein dual dantroline/ranolazine treatment was most effective. Our data are consistent with E99K mutant protein being a central cause of HCM but the three-way interaction between the primary genetic lesion, background (epi)genetics, and donor patient age may influence the pathogenic phenotype. This illustrates the value of isogenic hiPSC-CMs in genotype-phenotype correlations.

## Highlights

• Arrhythmia was a hallmark phenotype in E99K hiPSC-CMs, provoked by altered [Ca^2+^]
• Monoallelic expression of E99K cardiac actin affects only half the cell population
• Severe phenotypes in father's E99K hiPSC-CMs suggest influence of age & epigenetics
• Mechanistic insight facilitated drug rescue with combined dantroline/ranolazine

## Teaser

In this article Smith, Denning and colleagues show that the E99K-ACTC1 cardiac actin mutation is a central cause of HCM, but the three-way interaction between the primary genetic lesion, background genetics, and donor patient age may influence the pathogenic phenotype. Pharmacological rescue experiments demonstrated dual dantroline/ranolazine to be an effective treatment.

## Introduction

Cardiomyopathies are defined as primary disorders of contractility in heart muscle. Most classifications of cardiomyopathy divide the disease into acquired and inherited disease due to a mutation and into hypocontractile phenotype with reduced ejection fraction or hypercontractile phenotype with preserved ejection fraction (Maron et al., 2006). Hypertrophic cardiomyopathy (HCM) is a common clinical phenotype, found in up to 1 in 500 of the general population, and symptoms usually manifest in the second or third decade of life. HCM is characterized by thickened left ventricular walls, notably the interventricular septum, and a hyperdynamic myocardium with defective relaxation, with an enhanced susceptibility to arrhythmia that can lead to sudden death in a small proportion of patients. In the longer term, these abnormalities can lead to heart failure. Histologically HCM leads to myocyte disarray and interstitial fibrosis.

HCM is overwhelmingly an inherited disease, and in most cases mutations in one of the genes coding for the contractile and structural proteins of the cardiac muscle sarcomere is responsible (Marston, 2011). Understanding phenotype-genotype relations in HCM is complex. Several other inherited diseases, including restrictive cardiomyopathy and left ventricular non-compaction (LVNC), appear to be subsets of HCM and can share the same mutations. Moreover, the penetrance of the HCM phenotype is variable, since symptoms of carriers of HCM-linked mutations can range from asymptomatic to fatal cardiac dysfunction (Sedaghat-Hamedani et al., 2018). Insight into the biophysical mechanism behind cardiomyopathies has advanced significantly. It is now clear that an enhanced myofilament Ca^2+^ sensitivity is often the primary consequence of HCM mutations leading to the hyperdynamic phenotype (Spudich, 2014) and the enhanced susceptibility to arrhythmia (Huke and Knollmann, 2010). Yet the pathway to secondary HCM phenotypes, such as myocyte hypertrophy, myocardial disarray, and interstitial fibrosis, are not easily explained by sarcomeric gene defects alone and may be dependent upon genetic background.

Actin comprises the main component of the sarcomeric thin filament, with α-cardiac actin (encoded by *ACTC1*; Genbank: NM_005159) accounting for ∼80% of actin expression in mature cardiomyocytes (Olson et al., 1998). To date, 14 *ACTC1* mutations have been identified in patients diagnosed with one or more forms of cardiomyopathy, of which 11 are associated with HCM (Mogensen et al., 1999, Olson et al., 1998, Olson et al., 2000, Morita et al., 2008, Kaski et al., 2009, Olivotto et al., 2008). The most extensively studied of these mutations is c.G301A (p.Glu101Lys). Although this mutation occurs at codon 101 of the *ACTC1* gene, the mature protein in muscle has the amino acid substitution E99K due to the removal of two N-terminal amino acids during post-translational processing (Rubenstein and Martin, 1983).

Although mutations in *ACTC1* are relatively rare, the clinical consequences have been studied unusually thoroughly. Three clinical studies have all shown the E99K-ACTC1 mutation to present varying HCM-related phenotypes (Arad et al., 2002, Olson et al., 2000), with the most extensive study by Monserrat et al. (2007). This found diverse and overlapping E99K-ACTC1 phenotypes using clinical and morphological data from 94 cases. There were clear differences in the nature and severity of the clinical expression of the disease between carriers, but none was normal. HCM and LVNC were the most frequently reported phenotypes (Monserrat et al., 2007, Song et al., 2011). Hypertrophy was reported from 62 of 76 mutation carriers. The distribution of the hypertrophy was predominantly apical. Electrocardiography (ECG) investigation showed abnormalities in 53 of 61 carriers. Atrial fibrillation or flutter was found in 7 of 53. The 22 adverse events reported included eight sudden deaths (five in a single family).

Several different E99K-ACTC1 models have been developed to investigate the mechanisms behind these disease phenotypes. These include *in vitro* motility and laser trap assays (Debold et al., 2010), and reconstitution of human E99K-ACTC1 in the thin filament of bovine cardiac muscle fibers (Bai et al., 2015). Studies in transgenic E99K-ACTC1 mice are the most comprehensive (Song et al., 2011). These models have been useful in obtaining data on the properties of E99K-ACTC1, with increased Ca^2+^ sensitivity often reported for the mutant protein (Song et al., 2011, Song et al., 2013, Bai et al., 2015), although this is sometimes not observed and adds to the debate surrounding disease mechanism (Debold et al., 2010).

Nevertheless, while the α-cardiac actin sequence is identical in human and mouse, conclusions are still limited by the predominance of the α-myosin heavy chain isoform in the ventricles of mice versus the β isoform in humans. Differing levels of mutant sarcomeric transcripts than those occurring from a single mutant allele in human expression, and intrinsic differences in cardiac function between the species, including a 10-fold higher heart rate compared with humans, are important (Arad et al., 2002, Denning et al., 2016). There is need for a fully human *in vitro* E99K-ACTC1 model, in which protein interaction data can be more readily related to the patient phenotype and used to explain varied disease penetrance.

Here, we developed a human model of HCM by harnessing human induced pluripotent stem cell (hiPSC) reprogramming and CRISPR/Cas9 genome editing technology. By generating hiPSC lines from patients carrying the E99K mutation (E99K1 and E99K2) and a healthy non-carrier relative (NC) and then using CRISPR/Cas9 technology, we created a model whereby the mutation had been corrected in two isogenic pairs and introduced in the other. This model recapitulated many disease phenotypes including abnormal contractility, Ca^2+^ sensitivity/handling, arrhythmogenesis, and hypertrophic signaling. In almost all cases the aberrant phenotypes observed in E99K-expressing hiPSC-derived cardiomyocytes (hiPSC-CMs) were greater than in non-expressing isogenic counterparts, consistent with this mutant protein having a central role in HCM.

However, there were considerable variations in severity between the lines, most notably with hiPSC-CM phenotypes from the heterozygote father being most pronounced and widespread. This included contraction force and velocity, and relaxation time; hypersensitivity to low Ca^2+^; Ca^2+^ handling (contraction and relaxation); hypertrophic brain natriuretic peptide (BNP) expression; and expression of Ca^2+^ handling machinery (*CASQ2*, *CALM1*, *CAMK2D*, *PPP3cA*). Although we had not expected this, it may be attributed to the greater age of the father relative to his sons. Nevertheless, the E99K mutation perturbed Ca^2+^ signaling pathways in some instances, which enabled hypothesis-driven drug rescue experiments. Thus the Ca^2+^ handling modifier drugs, ranolazine and dantrolene, reduced hypertrophic signaling, with dual treatment being most effective.

## Results

### Generation of Isogenic Sets of E99K-ACTC1 hiPSCs by CRISPR/Cas9 Editing

**Figure 1:**
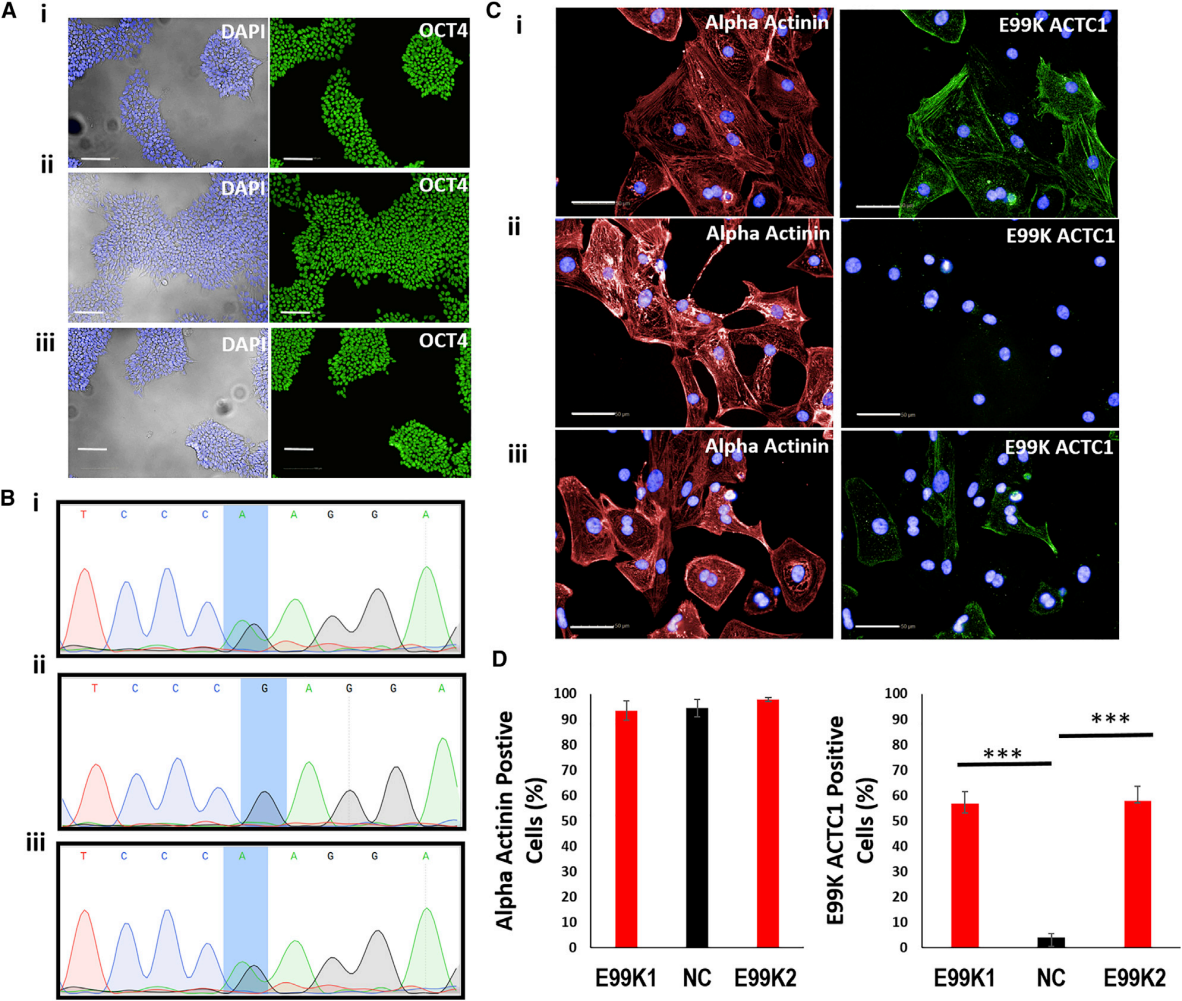
Production of hiPSC Cardiomyocytes with or without c.301G>A E99K Mutations (A) Relatives with (E99K1, E99K2) or without (NC) the mutation donated skin biopsies. Fibroblasts were reprogrammed into hiPSC using non-integrating CytoTune 2.0 Sendai virus and E6/E8 culture medium and stained for pluripotency marker, OCT4 (green; blue is DAPI). (B–D) In (B), sequencing confirmed the (C)301G>A ACTC1 mutation that causes the E99K polymorphic variant in diseased, but not healthy, lines. In (C), high-efficiency differentiation of hiPSC yielded cardiomyocyte purities of >90% (red is α-actinin staining; blue is DAPI). Counterstaining (green) with an antibody specific to the mutant E99K peptide showed that ∼50% of the diseased cardiomyocytes were positive, suggesting biallelic expression of both mutant and healthy alleles (C and D). In contrast, E99K reactivity was not detected in hiPSC cardiomyocytes from the healthy individual (C and D). n = 6. Scale bars, 100 μm (A) and 50 μm (C). Significance was determined by Student’s t test, ^∗∗∗^p < 0.001.

Skin-punch biopsies were obtained and reprogrammed to hiPSCs from three donors of the same family; a 48-year-old male patient carrying the c.*ACTC1*^G301A^ mutation (E99K1), the 14-year-old healthy c.*ACTC1*^G301G^ son (NC), and the 19-year-old son carrying the c.*ACTC1*^G301A^ mutation (E99K2) (Figure S1). The resulting hiPSC lines expressed the OCT4 pluripotency marker (Figure 1A) and had the expected heterozygote mutant (E99K1; E99K2) or wild-type (NC) *ACTC1* sequences (Figure 1B). Differentiation to high purity (>90% α-actinin^+^) cardiomyocytes (hiPSC-CMs) (Figures 1C and 1D) allowed reactivity to an antibody specific to the mutant E99K peptide to be tested. This showed positive protein expression in ∼50% of the E99K1 and E99K2 hiPSC-CMs, suggesting that only one allele of the *ACTC1* gene is active in any given cell. As expected, E99K peptide reactivity was not detected in NC hiPSC-CMs (Figures 1C and 1D).

**Figure 2:**
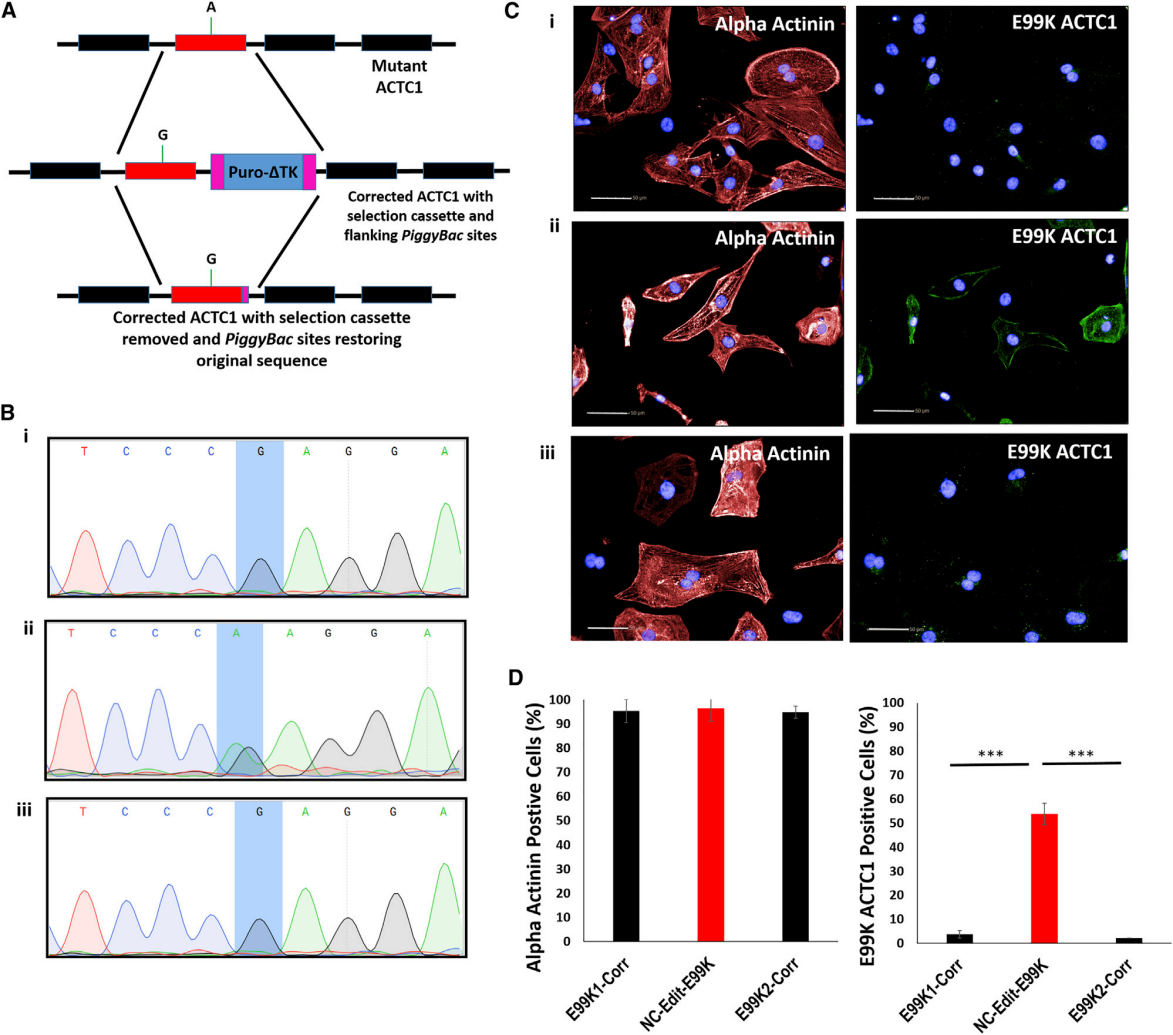
Generation of Isogenic E99K hiPSC Cardiomyocytes (A) A footprint-free *PiggyBac* targeting strategy of the ACTC1 gene was used to correct or induce E99K mutation into hiPSC lines. (B–D) In (B), sequencing confirmed the (C)301G>A ACTC1 mutation had been corrected in E99K1 and E99K2 and introduced in NC. In (C), high-efficiency differentiation of hiPSC still yielded cardiomyocyte purities of >90% after gene editing (red is α-actinin staining; blue is DAPI). Counterstaining (green) with an antibody specific to the mutant E99K peptide confirmed protein expression was lost in diseased patients (C and D). In contrast, E99K reactivity was now detected in hiPSC cardiomyocytes from the healthy individual (C and D). n = 6. Scale bars, 50 μm. Significance was determined by Student’s t test, ^∗∗∗^p < 0.001.

Isogenic controls were generated for E99K1, NC, and E99K2 hiPSCs using a footprint-free *PiggyBac* based CRISPR/Cas9strategy, coupled with PCR screening and sequence confirmation (Kondrashov et al., 2018) (Figure 2A). This identified targeted clones for E99K1 and E99K2 where the c.301G>A *ACTC1* mutation had been corrected (producing E99K1-Corr and E99K2-Corr) and introduced into one allele of NC (producing NC-Edit-E99K) (Figure 2B). Analysis by PCR of six potential mismatched guide sequences indicated that no off-target events had occurred in other genes (Figure S2).

These clonal lines still yielded high purity (>90% α-actinin^+^) hiPSC-CMs. However, E99K peptide reactivity was no longer detected in E99K1-Corr and E99K2-Corr, but was now present in ∼50% of the NC-Edit-E99K hiPSC-CMs. This confirmed successful genomic editing correlated with altered protein isoform expression, generating isogenic control lines (E99K1-Corr, E99K2-Corr, and NC-Edit-E99K) (Figures 2C and 2D). Thus, a suite of three isogenic pairs comprising six hiPSC lines was successfully created: E99K1 and E99K1-Corr; NC and NC-Edit-E99K; and E99K2 and E99K2-Corr.

### Contractility in Wild-Type and E99K EHT

**Figure 3:**
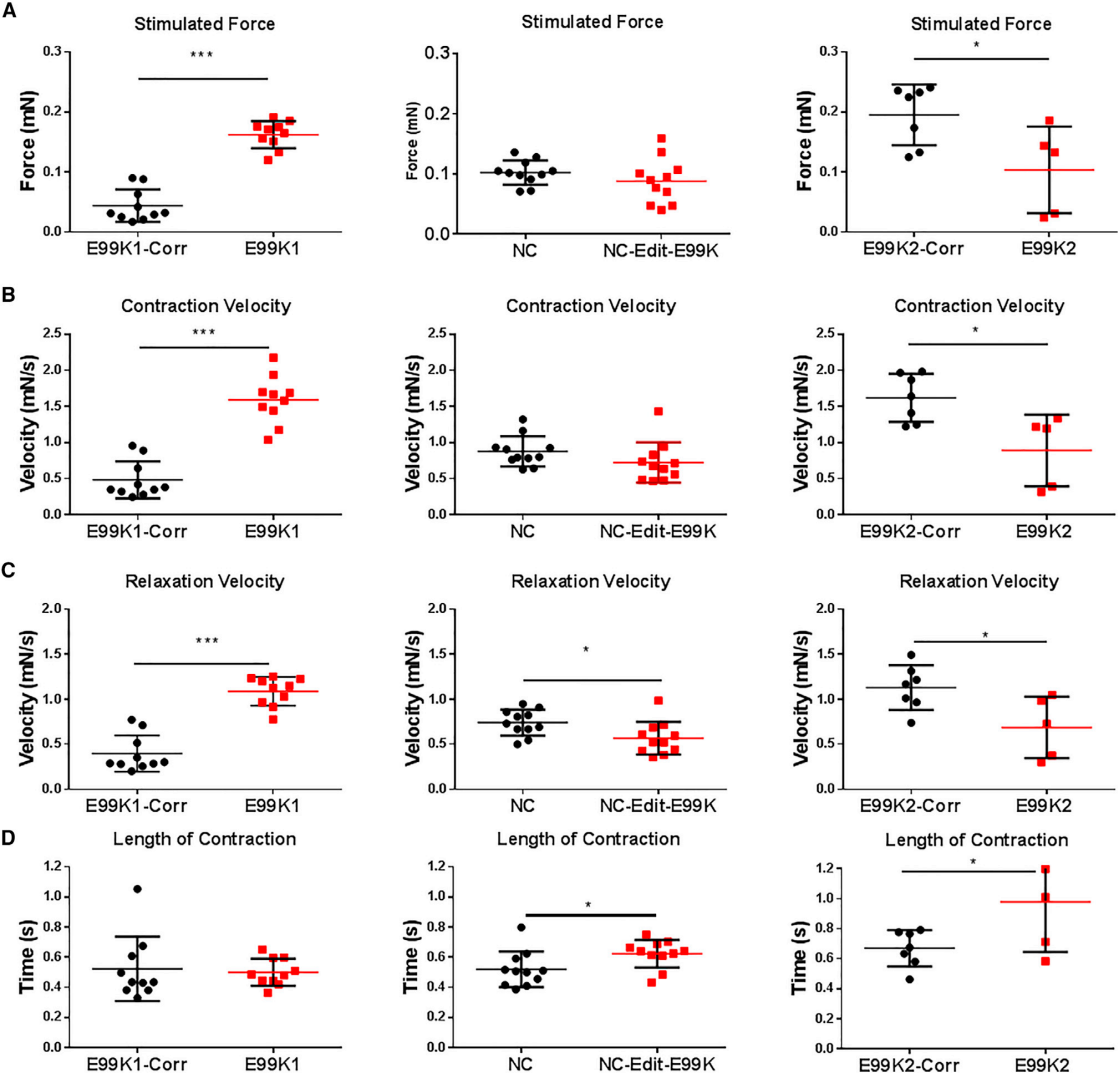
Stimulated Contraction of Isogenic Pairs of hiPSC-CM EHTs Auxotonic EHT contractions were recorded at 100 frames per second under stimulated conditions (1 Hz). Stimulated contraction force (A), contraction velocity (B), relaxation velocity (C), and length of contraction (D) are shown. All error bars represent SEM. Significance was determined by Student’s t test, ^∗^p < 0.05 and ^∗∗∗^p < 0.001. n = 10 E99K1, 10 E99K1-Corr, 11 NC-Edit-E99K, 11 NC, 5 E99K2, and 7 E99K2-Corr. Red, mutant ACTC E99K; black, wild-type. See also Figure S6.

hiPSC-CMs were successfully encapsulated into human engineered heart tissues (hEHTs), matured for 2 weeks, and stimulated at 1 Hz. We used image analysis of video microscopy for the calculation of contraction parameters as previously described (Eschenhagen et al., 2012). E99K1 had significantly stronger contractions than E99K1-Corr (Figure 3A); however; there was no significant difference between NC and NC-Edit-E99K lines, and E99K2 generated significantly less force than its corrected control.

Although the E99K1 line generated significantly more force, the overall length of contraction time was similar to E99K1-Corr. However, both NC-Edit-E99K and E99K2 lines had significantly longer contraction times than their wild-type isogenic controls (Figure 3D). When contraction and relaxation velocities were analyzed, E99K1 line had significantly faster speeds than their corrected counterpart. In contrast, NC-Edit-E99K and E99K2 were similar or significantly slower (Figures 3B and 3C). Thus, there were differences between E99K1 hEHT responses relative to NC-Edit-E99K- or E99K2-derived hEHTs.

**Figure 4:**
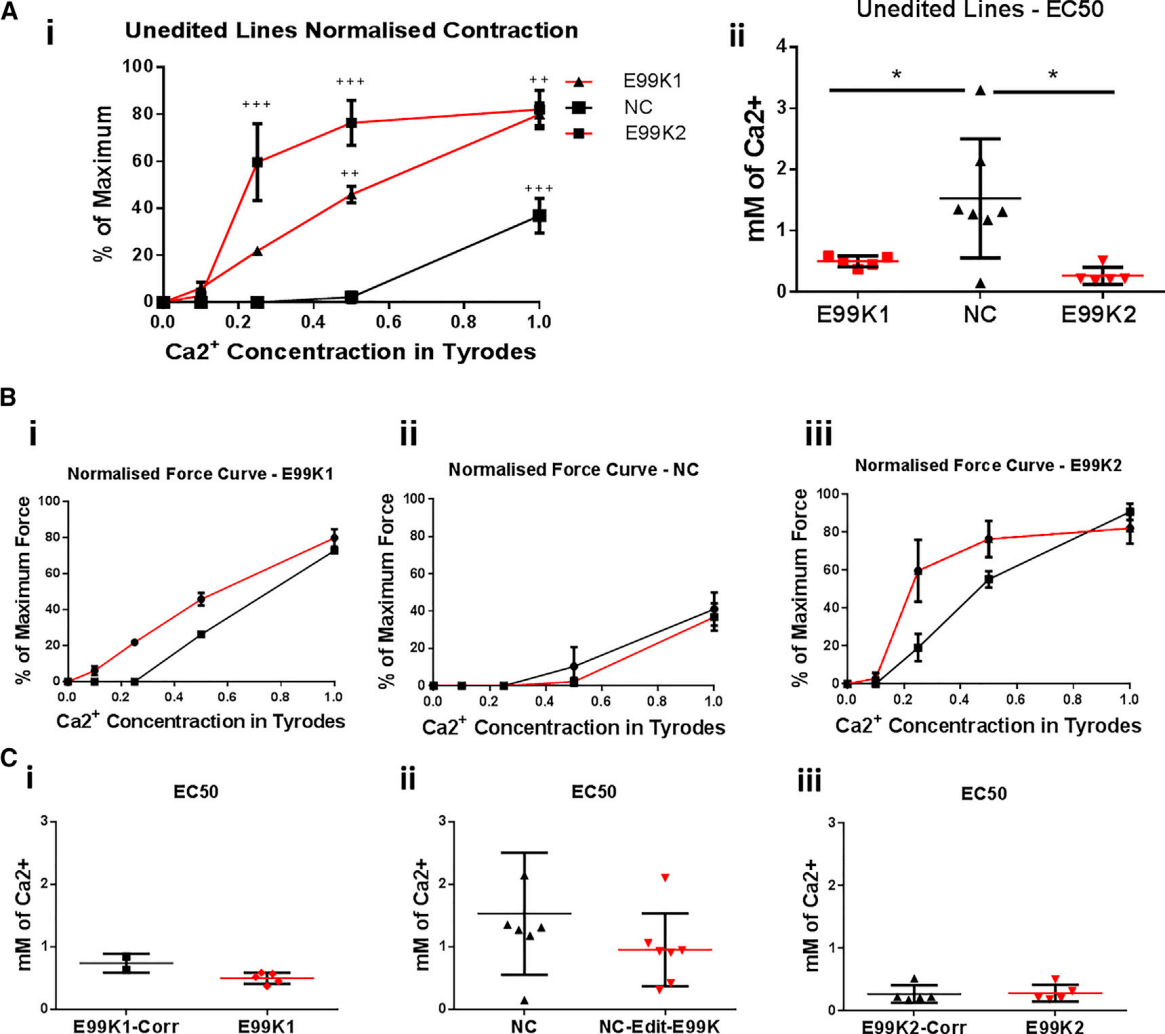
Ca^2+^ Sensitivity of Isogenic Pairs of hiPSC-CM EHTs iPSC-CM EHT were exposed to increasing concentrations of Ca^2+^ in Tyrode's solution and contractions were recorded while being stimulated at 1 Hz. In (Ai), the force of EHTs was calculated as a percentage of maximum for the unedited lines, with E99K lines shown in red and wild-type in black. EC_50_ values were calculated using absolute force and are shown for the unedited lines in (Aii). The comparison of normalized forces are shown for the isogenic pairs of E99K1 (Bi), NC (Bii), and E99K2 (Biii); the dotted line is the average with arrhythmogenic EHTs removed. In (Ci) to (Ciii), the corresponding EC_50_ values for isogenic lines were calculated using absolute force. All error bars represent SEM. Significance was determined by Student’s t test and one-way ANOVA, ^∗^p < 0.05, ^++^p < 0.01, and ^+++^p < 0.001. n = 7 E99K1, 2 E99K1-Corr, 7 NC-Edit-E99K, 7 NC, 5 E99K2, and 6 E99K2-Corr. Red, mutant ACTC E99K; black, wild-type.

In previous studies the E99K-ACTC1 hypercontractile phenotype was shown to be due to an increase in myofibrillar Ca^2+^ sensitivity (Song et al., 2011, Song et al., 2013). We therefore studied the effect of external Ca^2+^ concentration on EHT contractility (Figure 4). At 0 mM Ca^2+^ all EHTs were quiescent, whereas all were spontaneously beating at 0.5 mM Ca^2+^. Increased [Ca^2+^ ] dependence was seen when comparing unedited E99K lines (E99K1 and E99K2) against the unedited wild-type NC line in both normalized contraction amplitude at different Ca^2+^ concentrations and in their EC_50_ values (Figures 4Ai and 4Aii). However, when comparing each line with its isogenic counterpart, increased contraction at lower Ca^2+^ concentrations was not seen in any of the three pairs, and neither were changes in EC_50_ values (Figures 4Bi–4iii and 4Ci–4iii). Thus, overall we could detect increased [Ca^2+^] dependence in the unedited mutant lines compared with wild-type, but this phenotype was not knocked in or corrected in the isogenic pairs.

### E99K hiPSC-CMs Display Increased Arrhythmogenic Events

**Figure 5:**
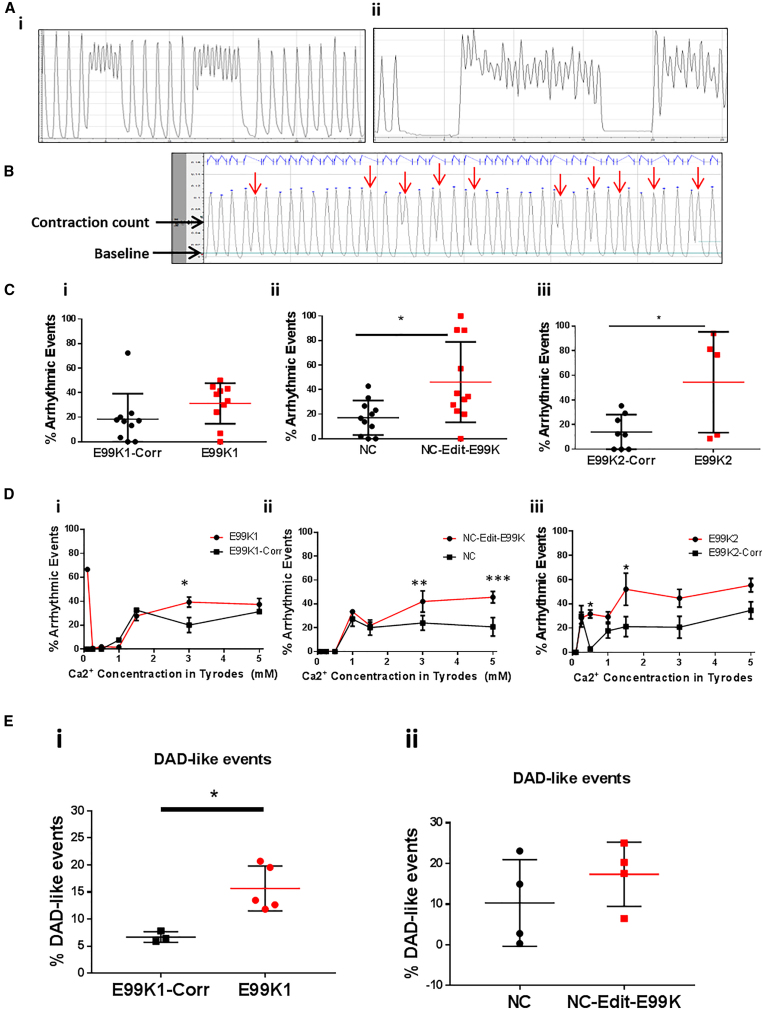
Arrhythmic Event Frequency in 3D hiPSC-CM EHTs and in 2D GECI Targeted hiPSC-CMs In (Ai) and (Aii), example traces of arrhythmic contractions are shown. In (B), analysis of an example trace in pClamp is shown with arrows pointing to abnormal contractions identified through using a baseline and contraction count line. In (C), EHTs were stimulated at 1 Hz, and arrhythmic events were counted and expressed as a percentage for E99K1 (Ai, n = 10, 10), NC (Ci, n = 11, 11), and E99K2 (Bi, n = 8, 5). In (D), arrhythmic events were counted in EHTs and expressed as a percentage during exposure to increasing Ca^2+^ concentrations, for E99K1 (Di, n = E99K1 7, E99K1-Corr 2), NC (Dii, n = 7 NC-Edit-E99K, 7 NC), and E99K2 (Diii, n = E99K2 5, E99K2-Corr 7). For 2D analysis hiPSC-CMs utilized genetically encoded expression of R-GECO1.0 or ratiometric GEM-GECO from the *AAVS1* locus. In (E), analysis of irregular Ca^2+^ transient events occurring during a 300-s line scan, with events lower than 75% mean peak amplitude, is shown for E99K1 and NC isogenic lines. E99K1-Corr n = 4, E99K1 n = 5, NC n = 4, NC-Edit-E99K n = 4. All error bars represent SEM. Significance was determined by Student’s t test, ^∗^p < 0.05, ^∗∗^p < 0.01, and ^∗∗∗^p < 0.001. Red, mutant ACTC E99K; black, wild-type. See also Figure S3B.

We next investigated whether E99K-ACTC1 mutants showed any difference in arrhythmogenic event frequency, since clinical and animal studies usually show ECG abnormalities and a varying degree of enhanced arrhythmia in mutation carriers (Arad et al., 2002, Olson et al., 2000, Monserrat et al., 2007, Song et al., 2011, Rowlands et al., 2017). Contraction traces obtained for EHTs clearly highlighted the presence of arrhythmogenic events (Figure 5A). To quantify these arrhythmogenic events in a non-biased manner, we used pClamp software as an unbiased method for analysis. First, the total twitch duration (combined contraction and relaxation time) was calculated for 211 E99K1, 218 NC, and 213 E99K2 wild-type contractions in seven EHTs. Twitch durations were averaged and found to be significantly different (p < 0.001) between the wild-type lines, at 0.51s for E99K1-Corr, 0.73s for NC, and 0.60 for E99K2 (Figure S3A).

Analysis then identified the number of abnormal contractions that failed to return to non-arrhythmogenic contraction time baseline during a normal event time (Figure 5B). For E99K1, 31% of contractions were found to be abnormal, although this was not significantly different when compared with wild-type isogenic controls (Figure 5Ci). NC-Edit-E99K and E99K2 had significantly more abnormal contractions when compared with their wild-type isogenic controls, with 54% and 46% abnormal contractions, respectively (Figures 5Cii and 5Ciii). No significant difference in the number of abnormal contractions was found between wild-type lines, with 18%, 17%, and 14% abnormal contractions for E99K1-Corr, NC, and E99K2-Corr, respectively.

We next evaluated whether Ca^2+^ concentration influenced arrhythmogenesis in EHTs fabricated from the six lines (Figure 5D). Relative to their wild-type isogenic counterparts, all E99K hEHTs showed more arrhythmogenic contractions, although significance was reached at differing Ca^2+^ levels. Thus, for E99K1 it was 3 mM Ca^2+^ for NC-Edit-E99K 0.5 mM and 1.5 mM Ca^2+^, and for E99K2 3 mM and 5 mM Ca^2+^.

To further support the 3D arrhythmogenic analysis, we implemented an alternative approach in a complementary 2D system. Nickase CRISPR/Cas9 was used to insert genetically encoded Ca^2+^ indicators (R-GECO1/GEM-GECO) into the safe locus *AAVS1* of the four parental and engineered hiPSC lines of E99K1 and NC. Correct insertion was confirmed by PCR and sequencing, while expression was validated by immunocytochemistry (Figure S4). Confocal line scans were taken during a 300-s time period to obtain traces and corresponding kymographs of spontaneous Ca^2+^ transients in hiPSC-CMs (Figures S4E and S4F). These line scans were used to calculate mean peak amplitudes and irregular delayed-after-depolarization Ca^2+^ transient events (events lower than 75% mean peak amplitude).

In 2D culture, arrhythmogenic events were shown to vary even at physiological Ca^2+^ concentration (1.8 mM), occurring at a frequency of 15.7% versus 6.7% for E99K1 versus E99K1-Corr and 17.4% versus 10.3% for NC-Edit-E99K versus NC (Figure 5E). Although there was no difference in R-GECO1 signal amplitude (F/F_0_) between E99K1 lines, NC-Edit-E99K hiPSC-CMs displayed a ∼50% lower GEM-GECO signal amplitude (F/F_0_) relative to NC controls (Figure S4G), indicating a lower systolic Ca^2+^ peak. These data demonstrated both the E99K1 and NC backgrounds to exhibit increased arrhythmogenic events when the E99K-ACTC1 mutation is present.

### E99K-Associated Abnormal Ca^2+^ Handling Is Absent when Introduced into Healthy Genetic Background

Due to its key regulatory role in excitation-contraction coupling, abnormal Ca^2+^ handling can contribute to severe disease phenotypes. HCM patients can exhibit prolonged Ca^2+^ transients (Lan et al., 2013), often attributed to the presence of sarcomeric protein mutations, such as E99K-ACTC1. Indeed, previous models have shown the E99K-ACTC1 mutation to increase Ca^2+^ sparks and Ca^2+^ transients in young transgenic mice (Rowlands et al., 2017).

**Figure 6:**
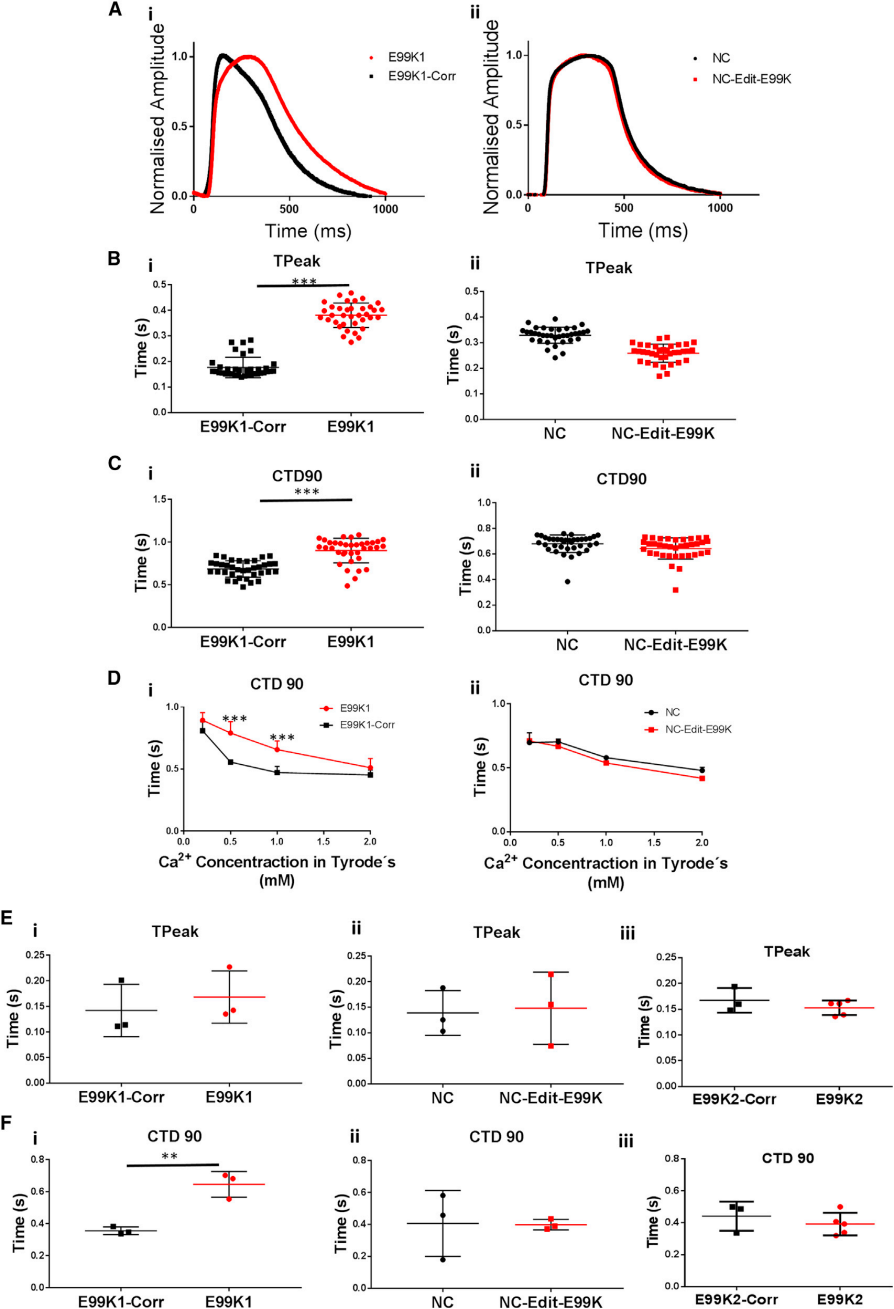
Ca^2+^ Handling Properties in 2D hiPSC-CMs and 3D EHTs hiPSC-CMs were loaded with Fluo4-AM and Ca^2+^ transients were recorded using CellOPTIQ technology (Ai and Aii). Time to peak (TPeak) was determined in each of the four lines (Bi and Bii). Technical n = 36. Ca^2+^ transient decline was measured in each of the DI and DII lines as a percentage of peak at 90% (Ci and Cii). Technical n = 36. Analysis was carried out on contractions during exposure to varied Ca^2+^ concentrations in Tyrode's solution (Di and Dii). n = 6. For 3D hiPSC-CM analysis, EHT were loaded with Fluo4-AM, stimulated at 1 Hz in Tyrode's solution at 37°C, and Ca^2+^ transients were recorded. EHT time to peak was determined in each of the six lines (Ei–Eiii). EHT Ca^2+^ transient decline was measured in each of the six lines as a percentage of peak at 90% (Fi–Fiii). n = 3 for all apart from E99K2 where n = 5. All error bars represent SEM. Significance was determined by Student’s t test, ^∗∗^p < 0.01 and ^∗∗∗^p < 0.001. Red, mutant ACTC E99K; black, wild-type.

To investigate whether abnormal Ca^2+^ handling could be detected, we used the CellOPTIQ cardiomyocyte analysis platform to assess intracellular Ca^2+^ transients in 2D cultures. Monolayers of hiPSC-CMs were loaded with Fluo4-AM, and Ca^2+^ transients were recorded using CellOPTIQ technology (Figure 6A). This 2D system showed that time to peak (TTP) was significantly altered (p < 0.001) in the E99K1 isogenic pair, with E99K1 exhibiting ∼115% longer TTP than E99K1-Corr. NC-Edit-E99K and NC did not differ (Figure 6B). Ca^2+^ transient decline was measured as time from peak to 90% return to baseline (CTD_90_). Analysis showed differences only in the E99K1 isogenic pair, with E99K1 exhibiting a 31.4% change relative to E99K1-Corr (Figure 6C).

Our earlier observations showed that contraction forces and arrhythmogenic events varied with differing extracellular Ca^2+^ concentrations (Figures 4A, 4B, and 5D). Therefore, Ca^2+^ transient analysis was carried at a range of extracellular Ca^2+^ concentrations to determine whether the abnormal transient decline was also dependent on the ability to handle extracellular Ca^2+^. Although all lines exhibited prolonged CTD_90_ at lower extracellular Ca^2+^ concentration (Figure 6D), there was no difference in response to these changes between the NC isogenic lines. The higher CTD_90_ increase in E99K1 compared with E99K1-Corr was significant at lower than physiological Ca^2+^ concentrations (0.5–1 mM).

We investigated the effect of a 3D environment architecture on mutant Ca^2+^ transients. EHTs were loaded with the Ca^2+^ indicator, Fluo4-AM, and stimulated at 1 Hz in Tyrode's solution at 37°C. Ca^2+^ transient parameters were obtained through analysis of Ca^2+^-dependent changes in fluorescence intensity within tissues. In 3D tissues there was no significant difference in TTP caused by the presence of the E99K-ACTC1 mutation (Figure 6E), but CTD_90_ data showed the same trend as the 2D model. In 3D EHTs, E99K1 CTD90 was 81.5% longer compared with E99K1-Corr, while NC-Edit-E99K and E99K2 showed no difference (Figure 6F). Collectively, both 2D and 3D data showed that the E99K-ACTC1 mutation caused abnormal Ca^2+^ handling in the genetic background of E99K1 but not in NC or E99K2.

### Targeting Ca^2+^Handling Pathways to Reduce Hypertrophic Signaling

To investigate whether the difference in phenotype severity was related to known hypertrophic signaling, we took forward isogenic pairs for E99K1 and NC. We investigated the expression of BNP because it has been reported to be >100-fold elevated in the plasma of HCM patients (Gardner, 2003). High-content imaging was used to classify cells in high, low, or negative BNP-expressing population, using predetermined empirical thresholds previously described for assessing BNP in hiPSC-CM lines (Carlson et al., 2013).

Analysis of BNP/cTnT/DAPI immunostained hiPSC-CMs (Figures S5A and S5B) showed no difference in BNP expression between NC-Edit-E99K and NC. In contrast, highly significant (p < 0.001) differences were seen in all three BNP populations for E99K1 versus E99K1-Corr, wherein E99K1 was 6.92- and 2.97-fold higher for BNP-high and -low cells, respectively, but 6.12-fold lower for BNP-negative cells. Thus, enhanced BNP expression in E99K-ACTC1 was only seen in the E99K1, but not NC genetic background, consistent with the difference in phenotype severities found for Ca^2+^ transients and contractility.

We hypothesized that the enhanced BNP expression seen in E99K1 could also be influenced by extracellular Ca^2+^, as a mechanism behind its abnormal phenotypes. Although we observed no change in BNP expression at extracellular Ca^2+^ spanning physiological levels (1.8 mM), a reduction of BNP expression was observed in 0.1 mM and 0.5 mM Ca^2+^ (Figures S5C and S5D), indicating a role of Ca^2+^ in hypertrophic signaling pathways.

**Figure 7:**
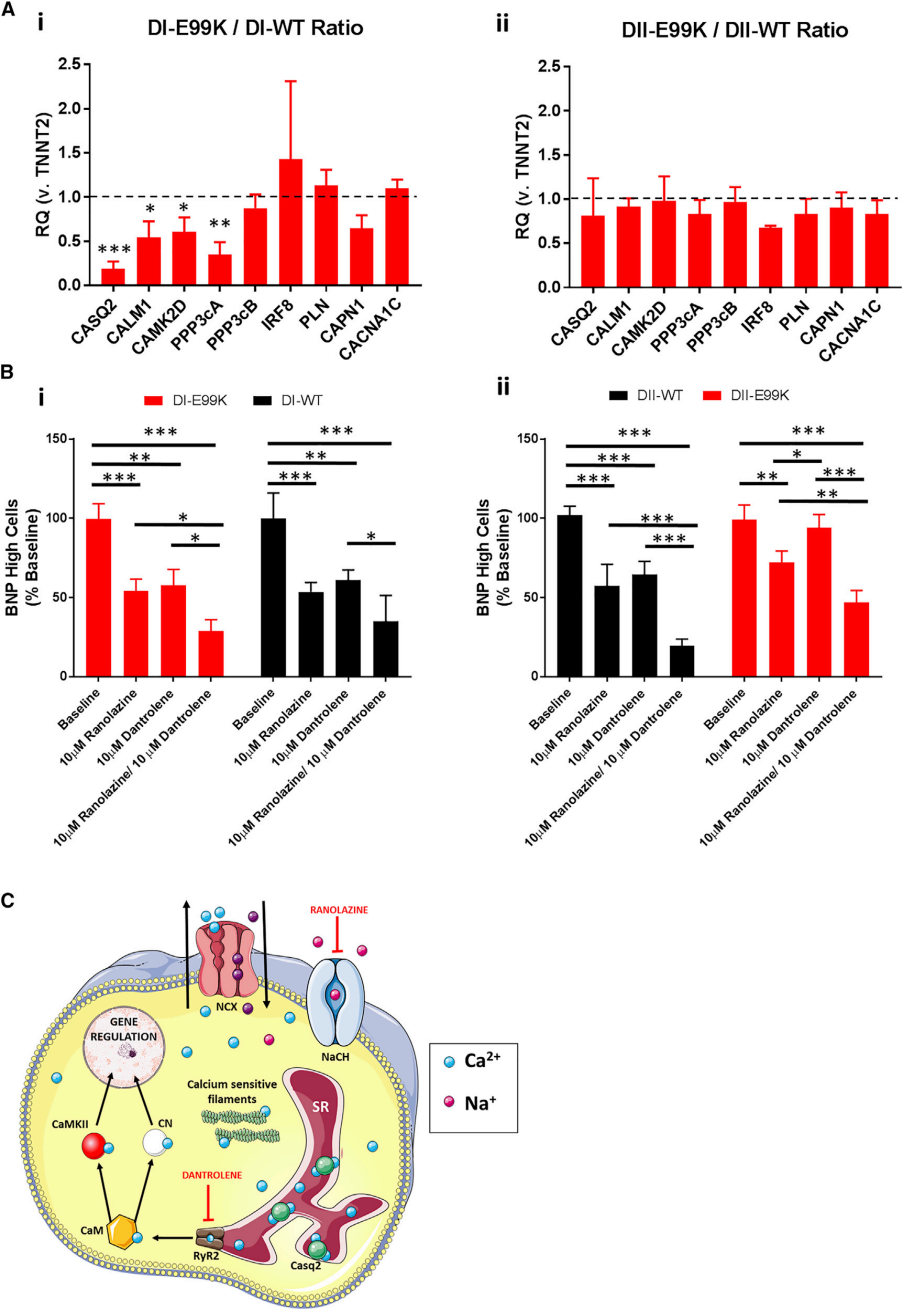
Ca^2+^ Signaling in Hypertrophic hiPSC-CMs (A) qPCR analysis of Ca^2+^ signaling gene expression ratios in E99K lines normalized to wild-type (WT) controls. (B) The effect of two intracellular Ca^2+^-altering drugs (ranolazine and dantrolene) on the percentage of highly expressing BNP hiPSC-CMs. (C) Intracellular Ca^2+^ signaling pathways and the mechanism ranolazine and dantrolene reduce cytoplasmic Ca^2+^ levels. n = 3. NCX, sodium-Ca^2+^ exchanger; SR, sarcoplasmic reticulum. Significance was determined by Student’s t test, ^∗^p < 0.05, ^∗∗^p < 0.01, and ^∗∗∗^p < 0.001. Red, mutant ACTC E99K; black, wild-type.

To investigate whether abnormal regulation of Ca^2+^ signaling pathways may be contributing to the mechanism of disease phenotypes in E99K-ACTC1 mutants, we next determined the expression levels of key Ca^2+^ signaling genes through a targeted qRT-PCR screen (Figure 7A). Analysis showed no differences between NC-Edit-E99K and NC. In contrast, E99K1 showed significantly (p < 0.05) lower expression of calmodulin (*CALM1*) and calmodulin-dependent protein kinase II delta (*CAMK2D*) than E99K1-Corr.

Although no difference was seen in expression of the Ca^2+^-binding regulatory subunit of calcineurin (*PPP3CB*), expression of the calmodulin-binding catalytic subunit of calcineurin (*PPP3CA*) was significantly (p < 0.01) lower in E99K1 than in E99K1-Corr. The greatest difference was seen in the expression of the sarcoplasmic reticulum Ca^2+^-binding calsequestrin 2 protein (*CASQ2*), with E99K1 showing 5.25-fold lower levels than E99K1-Corr. Expression of genes encoding the voltage-dependent Ca^2+^ channel (*CACNA1C*), the Ca^2+^ release inhibitor, phospholamban (*PLN*), and the Ca^2+^-sensitive cysteine protease, calpain 1 (*CAPN1*), did not differ between E99K1 and E99K1-Corr (Figure 7). This expression data associated well with the differences seen in disease phenotype between the donors and suggests a key role for Ca^2+^ signaling pathways in disease penetrance.

Altogether, these data suggested that targeting cytosolic Ca^2+^ levels, and specifically targeting sarcoplasmic reticulum Ca^2+^, release may reduce hypertrophic signaling. As such, ranolazine and dantrolene were identified as candidates with potential therapeutic benefit. Treatment with 10 μM of either drug significantly reduced BNP expression (p < 0.01) in all four lines, with the exception of dantrolene on NC-Edit-E99K (Figure 7B). Interestingly, in almost all cases, combined treatment with both drugs had a significantly greater effect (p < 0.05) than a single-drug regimen.

## Discussion

HCM is the most common inherited cardiomyopathy and has been investigated using *in vitro* systems and transgenic small animals. However, the relevance of these studies to HCM in the human heart is not known. Study of HCM in human heart has been limited due to the difficulties in obtaining fresh tissue. Therefore, HCM mutations in the context of patient-derived hiPSC-CMs are important for understanding the disease phenotype.

We investigated hiPSC derived from three members of an extended family with the E99K-ACTC1 mutation (father and son with the mutation and brother non-carrier). We used CRISPR/Cas9 to create isogenic pairs of all three in which the mutation had been corrected or introduced to develop a physiologically relevant humanized E99K-ACTC1 model. This gave us a toolset of six hiPSC-CM lines that have enabled us to distinguish direct mutant-dependent effects from secondary effects related to patient age or genetic background that may affect the phenotypic expression of HCM. The use of isogenics proved to be crucial because our original hypothesis proved to be incorrect; namely, that phenotypic aberration would be consistent between all E99K-expressing lines. While pathogenic phenotypes were often observed in E99K1 hiPSC-CMs (father), only some, and most notably arrhythmias, were evident in the E99K2 and NC-Edit-E99K lines created from his sons.

### The Cell Lines

Heterozygous hiPSC-CM from E99K1 and E99K2 showed expression of the E99K mutation, detected by mutation-specific antibody as did the CRISPR/Cas9-converted hiPSC-CM from NC. Interestingly, the expression of the mutation was segregated with 50% of cells expressing E99K and 50% of cells not expressing it. This indicates a stochastic monoallelic expression of *ACTC1* (Eckersley-Maslin and Spector, 2014). A similar monoalleleic expression of myosin heavy chain mRNA was suggested for the MYH7 R273G mutation (Kraft et al., 2016).

The significance of this is unknown but may be worth further investigation. In particular it has been speculated that a mosaic of wild-type and mutant cells having different contractile properties could disrupt cell-cell mechanical interactions and be the trigger for myocyte disarray and the subsequent interstitial fibrosis (Kraft et al., 2016). Interestingly, it is already known that in cardiac muscle the skeletal and cardiac actin isoforms are expressed is separate cells (Suurmeijer et al., 2003). The percentage of mutation expressed is proposed to be comparable with the pathological distribution of mutant to wild-type protein within the hearts of individuals affected with heterogeneous autosomal dominant cardiac disease: The 50% overall expression of the mutant protein compares with measurements of 39% (left ventricular free wall) and 19% (atrial-ventricular septum) in biopsies of two E99K patients (Song et al., 2011).

### Contractility

In the transgenic mouse model, the E99K mutation increased myofilament Ca^2+^-sensitivity of isometric force about 2-fold (Song et al., 2011). When EHT are activated maximally at 1.8 mM Ca^2+^ the maximum contraction, which in this case is auxotonic rather than isometric, ranged from 0.1 to 0.2 mN. There did not appear to be any detectable difference between native and CRISPR/Cas9 edited wild-type or E99K lines isogenic pairs. However, a large shift in [Ca^2+^] dependence was detected in unedited lines, with E99K EHTs having stronger contractions at lower levels of external Ca^2+^. The millimolar concentration of Ca^2+^ in the medium is indirectly related to the micromolar [Ca^2+^] in the sarcoplasm that activates contraction. It was observed that the threshold [Ca^2+^] to activate contraction was lower for the unedited E99K mutant samples. Similarly, the EC_50_ of E99K EHTs was lower than that of the wild-type. This is compatible with the well-documented enhanced myofibrillar Ca^2+^ sensitivity of the E99K mutation *in vitro* and in the mouse model.

NC-Edit-E99K and E99K2 both had longer contraction times and decreased velocity of contraction compared with their isogenic controls. Both these isogenic pairs had the same phenotype even when one had the mutation knocked in and one had the mutation corrected. E99K1 has a high contraction rate. Analysis of sarcomere assembly showed a trend, albeit non-significant, toward increased disarray in E99K-expressing hiPSC-CMs (Figure S7). However, there was no apparent correlation between this parameter and the contraction measurements recorded.

For comparison with E99K transgenic mice, we see about 2-fold slower relaxation times but similar contraction times in isolated myofibrils, papillary muscle, and cardiomyocytes, compatible with a longer overall twitch time (Song et al., 2013, Rowlands et al., 2017). Moreover, recombinant human E99K actin also shows decreased association, dissociation, and slower velocities of interaction with myosin than wild-type (Bookwalter and Trybus, 2006).

### Arrhythmia and Ca^2+^ Handling

ECG abnormalities are a hallmark of HCM and are commonly observed in the E99K patient cohort (Monserrat et al., 2007). The increased myofilament Ca^2+^ sensitivity caused by HCM mutations alters intracellular Ca^2+^-buffering(Robinson et al., 2018), and this has been shown to be sufficient to provoke arrhythmia and ECG abnormalities in mice (Huke and Knollmann, 2010). An enhanced probability of arrhythmia has also been observed with the E99K mutation in transgenic mice (Song et al., 2011). Moreover, an increase in Ca^2+^ sparks and Ca^2+^ transients was observed that showed variable penetrance depending on background mouse strain (Rowlands et al., 2017). This trait was apparent in our hiPSC-CM EHTs: contractile arrhythmia increased at higher [Ca^2+^] in both wild-type and E99K, but E99K EHTs showed a significantly greater frequency of arrhythmogenic events. This was further confirmed by studies using GECI Ca^2+^ sensors in 2D culture.

The difference in phenotype severity between the donors (E99K1 and NC) associates with expression of the natriuretic peptide, BNP, a known marker of hypertrophy. Expression was shown to be modulated by extracellular Ca^2+^ levels, indicating a regulatory role of Ca^2+^ in hypertrophic response. Subsequent analysis identified abnormal expression of calmodulin signaling pathway components in the severe, but not the mild, phenotype. This pathway is considered to be the primary sensor of changes in cellular free Ca^2+^ levels. As such, reduced expression of calmodulin, and the Ca^2+^sensitive enzymes it activates (CaMKII and calcineurin), is likely to reduce the regulation of Ca^2+^ handling that is crucial for normal cardiac function. This pathway has previously been implicated in hypertrophy through its ability to regulate natriuretic peptide expression (Dewenter et al., 2017), supporting the hypothesis that abnormal Ca^2+^ regulation in E99K1 is the mechanism behind the greater severity of hypertrophic phenotype.

Interestingly, the greatest expression difference between the E99K1 isogenic lines was in *CASQ2* expression. Calsequestrin 2 is the main Ca^2+^ buffering protein of the sarcoplasmic reticulum. Reduced levels of calsequestrin 2 have been shown to increase the probability of spontaneous Ca^2+^ release for any given luminal free Ca^2+^ concentration (Faggioni and Knollmann, 2012). Coupled with reduced calmodulin-driven Ca^2+^ regulation, a cellular environment with increased Ca^2+^ release from the sarcoplasmic reticulum would further exacerbate the hypertrophic phenotype seen in E99K1.

### The Origin of Phenotypic Variability

The common features of the E99K hiPSC-CM are compatible with the known role of the mutation in increasing myofilament Ca^2+^-sensitivity and disrupting Ca^2+^ handling. However, there was considerable variability between the three patient samples, with E99K1 showing the greatest array of severe phenotypes. This may reflect pathophysiology and/or age, particularly given that HCM typically becomes symptomatic in the 2^nd^ and 3^rd^ decades of life. E99K1 was 48 years old with overt symptoms of HCM and LVNC, and conduction deficit, and thus is likely to have developed secondary abnormalities (Figure S1). In contrast, E99K2 was 19 years old and did not show abnormal ECG abnormalities. NC was 14 years old and, in this case, the mutation was introduced after the sample was taken, so one would expect there to be no secondary abnormalities.

Mutation load in mitochondrial DNA is higher in hiPSCs produced from older individuals (Kang et al., 2016). An age-dependent increase in mitochondrial DNA mutations, leading to increased reactive oxygen species production and activation of hypertrophic signals, has been proposed as the mechanism behind an age-dependent development of a cardiac hypertrophic phenotype (Marian, 2001). Mouse models have previously shown age to influence E99K-ACTC1-associated phenotypes, with older E99K-ACTC1 mice developing increased end-diastolic and end-systolic volumes (Song et al., 2011).

Thus, speculatively our model recapitulates a varied phenotype severity through an age-dependent, rather than a genetic background, based mechanism.

### Limitations of the hiPSC System for This Study

We minimized technical variation by choosing directly related family members, from whom biopsies were harvested, prepared, and reprogrammed to hiPSC at the same time using the same methods; this extended to differentiation method, stage of differentiation, and cardiomyocyte purity, with cultures typically containing >90% α-actinin-positive cells (e.g., Figures 1 and 2). Assays were run on the same day wherever possible. Two clones per isogenic sets were used, wherein off-target events were not detected and genetic stability was retained, at least at the resolution of G-banding karyogram of 30 metaphase spreads. Nevertheless, any cells undergo stochastic epigenetic/genetic change relative to time in culture (Allegrucci et al., 2007, Smith and Whitney, 1980), with an additional issue for hiPSC being the retention of residual epigenetic memory that is refractory to reprogramming (Peng et al., 2011, Rowlands et al., 2017).

Differentiation was highly efficient, but the effect of the residual 1%–10% non-cardiomyocytes, which include mesodermal derivatives such as fibroblasts and smooth muscle cells (Sharma et al., 2015), can influence cell function. Deliberate mixing of hiPSC-CMs with non-cardiomyocyte populations (e.g., endothelial cells and fibroblasts) is known to alter force generation and drug responses (Ravenscroft et al., 2016). Purity of hPSC-CMs can be improved by techniques such as metabolic or genetic selection (Anderson et al., 2007, Sharma et al., 2015). However, these approaches place additional stress on the cardiomyocytes, are cumbersome, and/or require additional prolonged culture periods to genetically engineer individual hiPSC lines, which risks introducing further epigenetic/genetic change.

The ideal way to overcome these limitations is to increase the number of hPSC lines within the study and ensure each is matched with a Cas9/CRISPR isogenic control. However, there are currently no reports of high-volume hiPSC production, with multiple clones and isogenic pairing, likely due to the labor required for such an initiative.

Finally, it is well established that hiPSC-CMs immature relative to adult cardiomyocytes (see, e.g., Denning et al., 2016 for review). This includes the machinery that underpins HCM, including Ca^2+^ handling, t-tubules, sarcomeric alignment, and mitochondrial content. Despite these deficiencies, hiPSC-CMs are becoming established as a modality to evaluate successfully the impact of disease and drugs on cardiomyocyte function (Mosqueira et al., 2018, Gintant et al., 2017). The concerted international effort to improve hiPSC-CM maturity via approaches such as metabolic switching (Correia et al., 2017) and electrical pacing (Sun and Nunes, 2016) will further advance the utility of these models.

### The Use of E99K hiPSC-CM in Drug Screening

Figure 7C shows an overview of cytosolic Ca^2+^ released from the sarcoplasmic reticulum, activating calmodulin-dependent signaling pathways, that ultimately alters gene expression. Blocking sarcoplasmic reticulum Ca^2+^ release through inhibiting the ryanodine receptor-2 with dantrolene, and promoting Ca^2+^ efflux through enhancing the sodium-Ca^2+^ exchanger with ranolazine, lowers cytosolic Ca^2+^ and reduces hypertrophic signaling. We demonstrated that targeting of intracellular Ca^2+^ levels, by promoting Ca^2+^ efflux (ranolazine) and limiting sarcoplasmic reticulum Ca^2+^ release (dantrolene), reduced hypertrophic signaling.

Ranolazine was identified as a therapeutic candidate as an enhancer of the sodium-Ca^2+^ exchanger (outward mode) by blocking late sodium current and thereby indirectly promoting Ca^2+^ efflux. Contrastingly, the mode of action for dantrolene directly limits Ca^2+^ release from the sarcoplasmic reticulum during systole through its activity as a ryanodine receptor antagonist. These drugs have previously been shown to have a potential benefit in other HCM models, with ranolazine used to ameliorate diastolic function in HCM patient-derived cardiomyocytes (Coppini et al., 2012), and dantrolene applied as an antiarrhythmic agent in mouse models of HCM (Jung et al., 2012, Okuda et al., 2018). Here we show these drugs to have potential benefit in cases of E99K-ACTC1-associated HCM, and our results indicate that a dual treatment targeting both mechanisms may have enhanced therapeutic benefit. Dual treatments such as this have not been reported clinically and, as such, hiPSC-CMs provide an opportunity to trial new combinations with potential patient benefit. Moreover, the model we present now provides opportunities for high-throughput and/or high-content evaluation of the ability of drugs, singly or in combination, to rescue the phenotypes identified, particularly using hiPSC-CMs derived from the heterozygote father E99K1.

## Experimental Procedures

### hiPSC Generation

Fibroblast cultures were expanded in fibroblast growth medium and transduced using CytoTune2.0-iPS Sendai Reprogramming (Thermo Fisher #A16517). Following transduction, cultures were changed to Essential 6 medium (Life Technologies #A1516401) supplemented with 100 ng/mL basic fibroblast growth factor (Peprotech #100-18B) and medium exchanged daily until colonies appeared (5–10 days), after which the medium was changed to Essential 8 medium (Life Technologies #A1517001). hiPSC colonies were expanded and isolated by manual dissection. Two clones were used per line and used in subsequent studies.

### CRISPR/Cas9 Genome Editing of hiPSC

The strategy for correcting/introducing the E99K-ACTC1 mutation to generate isogenic control lines has been previously described in detail (Kondrashov et al., 2018). In brief, an *ACTC1* targeting vector was constructed containing a dual drug selection cassette (Puro-ΔTK) flanked by *PiggyBac* recombination sites, and the two *ACTC1* sequences (1 kb upstream and 1 kb downstream) homologous to the endogenous target locus cut site. One microgram of *ACTC1* targeting vector was transfected into 1 × 10^6^ hiPSCs, with 1 μg of hCas9 plasmid and 1 μg of guide RNA pU6 vector using an Amaxa 4D system (Lonza). Twenty-four hours after transfection, the medium was supplemented with 0.25 μg/mL puromycin (Life Technologies #A1113802) for positive selection of clones for up to 2 weeks. The puromycin-positive clones were then harvested using TrypLE (Life Technologies #12563029), cultured for 24 hr, and transfected with 3 μg of transposase plasmid transfection using Fugene HD transfection reagent (Promega #E2311). On the next day, cells were exposed to medium containing 2 μg/mL ganciclovir (Sigma #G2536) for negative selection of *PiggyBac* excision clones for up to 2 weeks.

### Monolayer Cardiac Differentiation of hiPSCs

hiPSC differentiation was performed by seeding vessels at approximately 20,000–40,000 cells/cm^2^, as described above. Fresh E8 was added the next day and the pre-conditioning step of hiPSC was performed the day after, by adding a Matrigel overlay (Matrigel diluted 1:100 in StemPro34 Serum Free Medium [SP34, Gibco #10639011]), supplemented with 1 ng/mL BMP4 (R&D Systems #314-BP-050). Approximately 16 hr later, medium was replaced by SP34 supplemented with 8 ng/mL activin A (ActA, Life Technologies #PHC9564) and 10 ng/mL bone morphogenetic protein 4 (BMP4). Forty-eight hours later, medium was changed to RPMI supplemented with B27 without insulin (−INS, Life Technologies #A1895601) and KY0211 (R&D #4731) and XAV939 (R&D #3748), both at 100 μM. These small molecules were added again 2 days later, in RPMI supplemented with B27 with insulin (+INS, Life Technologies #0080085-SA) instead. Thereafter, medium was changed every 2–3 days by fresh RPMI + B27 + INS until day 15 of differentiation, when spontaneously beating hiPSC-CM were dissociated and replated as below, and kept in RPMI + B27 + INS for approximately 10 days until phenotypic assays were performed.

### hEHT Fabrication and Maintenance

hEHTs were fabricated as previously described (Breckwoldt et al., 2017, Schaaf et al., 2014). In brief, Teflon spacers (EHT Technologies #C0002) were inserted in 2% ultra-pure agarose (Invitrogen #15510-027) solution pipetted into 24-well plates (Nunc) before gelification. Thereafter, spacers were removed and silicone racks (EHT Technologies #C0001) were placed in the aperture left by the agarose casting molds. Subsequently, freshly dissociated hiPSC-CMs were resuspended in DMEM (Biochrom, F0415), supplemented with 10% heat-inactivated fetal calf serum (Biochrom #S0615), 1% penicillin/streptomycin (PEST, Gibco), 2 mM L-glutamine, 2× DMEM (equalizing the hypotonic volume of fibrinogen plus thrombin), 10% Matrigel, 0.1% Y-27632, and 5 mg/mL fibrinogen (Sigma #F8630). The CM fibrinogen mix was then quickly mixed with 3 U of thrombin (Sigma #T7513) and pipetted into the aperture between the silicone posts. Forming hEHTs were then incubated for 2 hr at 37°C and 7% CO_2_ and subsequently moved to new 24-well plates filled with DMEM supplemented with 10% horse serum, 10 μg/mL insulin (Sigma #I9278), 33 μg/mL aprotinin (Sigma #A1153), and 1% (v/v) PEST, termed EHT medium. Each hEHT consisted of 1 million cells and was fed every other day for 2–3 weeks.

### Analysis of hEHTs

Contractile force was analyzed as previously described (Mannhardt et al., 2016). In brief, 2- to 3-week-old hEHTs were immersed in modified Tyrode's solution (120 mM NaCl, 5.4 mM KCl, 1 mM MgCl_2_, 0.4 mM NaH_2_PO_4_, 22.6 mM NaHCO_3_, 5 mM glucose, 0.05 mM Na_2_EDTA, and 25 mM HEPES) and varied Ca^2+^ concentration (0–5 mM CaCl_2_), and the 24-well plate was placed inside a transparent chamber to maintain homeostatic temperature (37°C), CO_2_ (5%), and O_2_ (40%). Automated video-optical recordings of silicone post deflection were enabled by the EHT analysis instrument (EHT Technologies #A0001) whereby a video camera placed above the chamber tracked hEHT movement and a separate computer running a customized software (CMTV GmbH) determined contractile force based on the known mechanical properties of the silicone posts. When indicated, EHTs were electrically paced (2 V, 1–2 Hz, impulse duration 4 ms) with carbon electrodes using a Grass S88X stimulator (Astro-Med). The contraction peaks were analyzed in terms of force, and contraction (T1) to peak from 20% peak height and relaxation time (T2) from peak to 80% of peak height. To quantify arrhythmogenic events without bias, we used pClamp software to identify the number of abnormal contractions compared with normal baseline events.

### Statistics

Statistical analysis was performed using GraphPad Prism (v7, GraphPad, La Jolla, CA, USA) software, evaluated by Student's t tests or unpaired one-way ANOVA, to compare isogenic pairs or between genotypes. Differences were considered significant at ^∗^p < 0.05, ^∗∗^p < 0.01, and ^∗∗∗^p < 0.001. Numbers denoted “n” indicate independent experiments unless stated otherwise.

## Author Contributions

J.G.W.S. and T.O. contributed equally to this work. J.G.W.S and T.O. took lead on performing experimental work, with J.G.W.S leading 2D experiments and T.O. leading 3D experiments. J.R.B., D.M., E.S., I.M., A.P., R.B.-V., and L.M. performed some experimental work. J.G.W.S. and T.O. wrote the manuscript with support from C.D., S.M., T.E., A.H., R.B.-V., L.M., and S.E.H. The project was conceived and supervised by C.D., S.M., T.E., A.H., and S.E.H.

## Supplemental Information

Document S1. Supplemental Experimental Procedures and Figures S1–S7

Document S2. Article plus Supplemental Information
