# Supplementary material for: Isogenic Pairs of hiPSC-CMs with Hypertrophic Cardiomyopathy/LVNC-Associated ACTC1 E99K Mutation Unveil Differential Functional Deficits
Source: Stem Cell Reports. 2018 Nov 1;11(5):1226–43. doi: 10.1016/j.stemcr.2018.10.006 (PMC6235010; doi:10.1016/j.stemcr.2018.10.006)
Supplement: Document S1. Supplemental Experimental Procedures and Figures S1–S7 [file mmc1.pdf]

Stem Cell Reports, Volume *11*

Supplemental Information

Isogenic Pairs of hiPSC-CMs with Hypertrophic Cardiomyopathy/ LVNC-Associated ACTC1 E99K Mutation Unveil Differential Functional Deficits

James G.W. Smith, Thomas Owen, Jamie R. Bhagwan, Diogo Mosqueira, Elizabeth Scott, Ingra Mannhardt, Asha Patel, Roberto Barriales-Villa, Lorenzo Monserrat, Arne Hansen, Thomas Eschenhagen, Sian E. Harding, Steve Marston, and Chris Denning

Supplementary Figures

a)

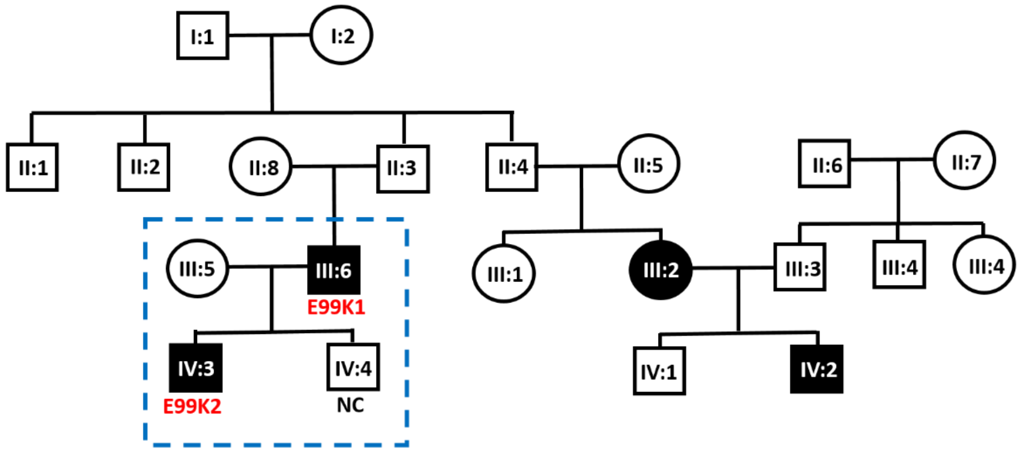

b)

Sample	Age of Diagnosis	Age of iPSC production	Maximum WT (mm)	Segment with maximum WT	Compact thickness (mm)	Compact/ non-compact	Left atrium diameter (mm)	LV diameters (end diastolic/end systolic) (mm)	Electrocardiogram	Treatment
E99K1	28	48	23	Apical	12	0.52	37	46	Abnormal Q	Bisoprolol
NC	-	14	-	-	-	-	-	-	-	-
E99K2	11	19	20	Apical	8	0.4	29	44	Normal	None

Figure S1. Details of donor samples. (a) Family tree showing the known E99K carriers and highlighting the three individuals from whom iPSC were prepared. **(b)** Clinical details of the three individuals from whom iPSC were prepared.

a)

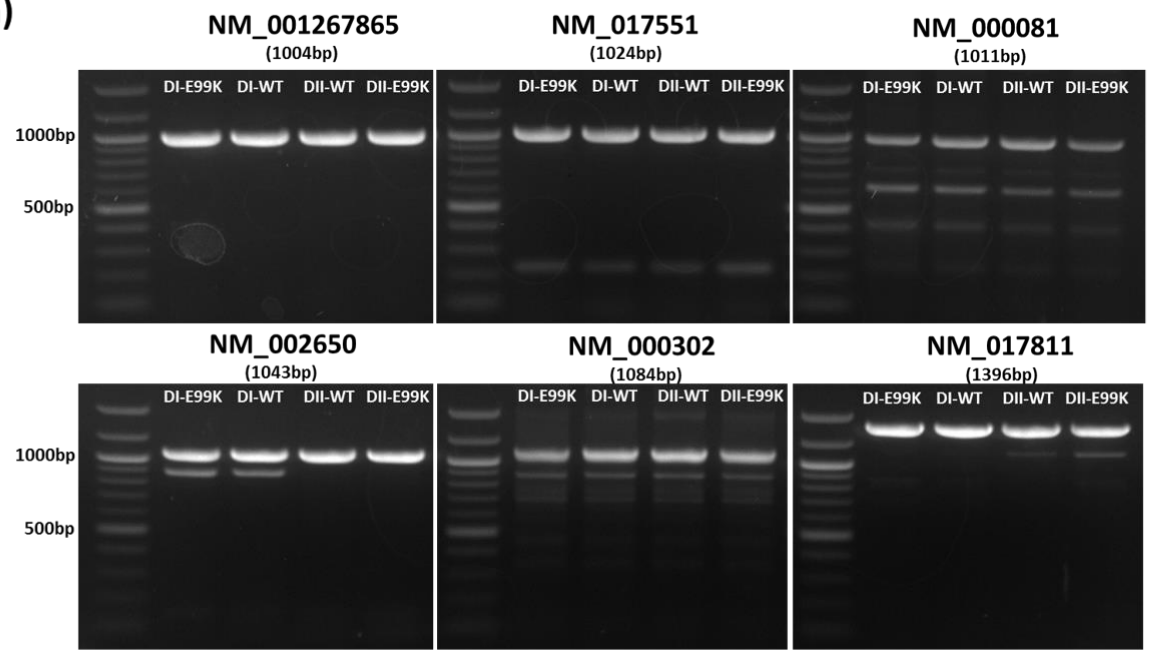

b)

Gene: Offtarget:		NM_001267865 GAGATAACACTG ATGCCCTGGAG (4MMs)	NM_017551 AGGTGAACAGTA GTCCCCTGGAG (4MMs)	NM_000081 CAATTAACAGTA GTTCCCTCTAG (4MMs)	NM_002650 GAGTTCTCAGGA GTGCCCTGCAG (3MMs)	NM_000302 GAGCAAACCGTA GTCCCCTGGAG (4MMs)	NM_017811 GAGTTGACACTA GTGCCCAGGGG (3MMs)
Isogenic pair alignment	E99K1-Corr / E99K1	100%	100%	100%	100%	100%	100%
	NC-Edit-E99K/ NC	100%	100%	100%	100%	100%	100%

Figure S2. Analysis of potential mismatched guide sequences. (a) DNA electrophoresis gels showing the PCR amplification of six genes identified as containing the most likely potential mismatched guide sequences (3 or 4 mismatched bases). **(b)** DNA sequencing of the PCR products showed complete alignment with untargeted donor lines with no INDELs present, indicating no off-target events had occurred.

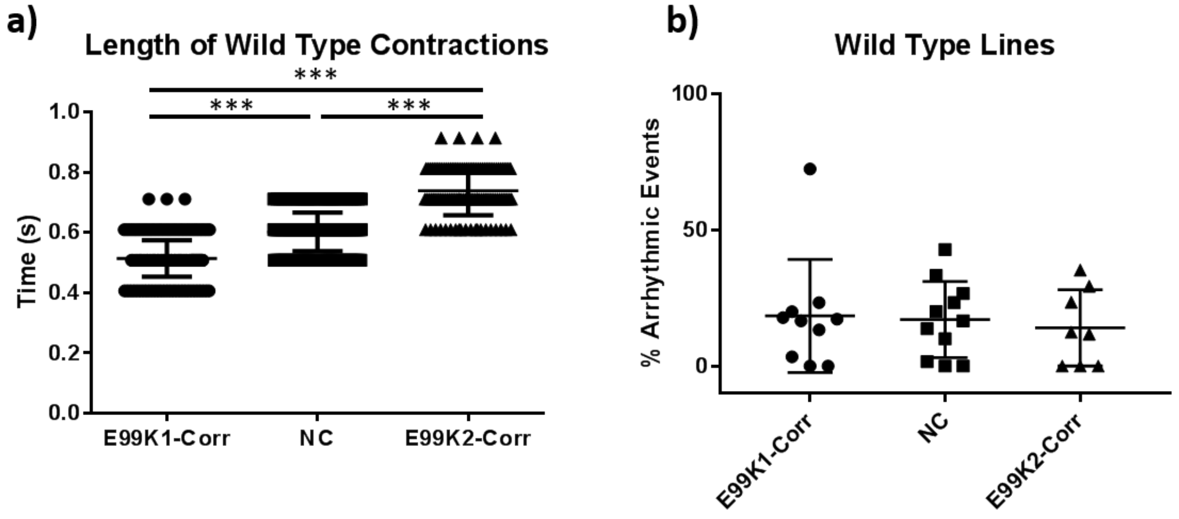

Figure S3. Wild type EHT analysis. a) Total twitch duration (combined contraction and relaxation time) was calculated calculated by pClamp software and averaged for 211 E99K1-Corr, 218 NC and 213 E99K2-Corr wild-type contractions in 7 EHTs. b) Arrhythmic event frequency in wild type 3D hiPSC-CM EHTs. Wild type EHTs were stimulated at 1Hz and arrhythmic events were counted and expressed as a percentage for E99K1 (N=10), NC (N=11), and E99K2 (N=8). All error bars represent standard error of the mean. Significance was determined by one-way ANOVA.

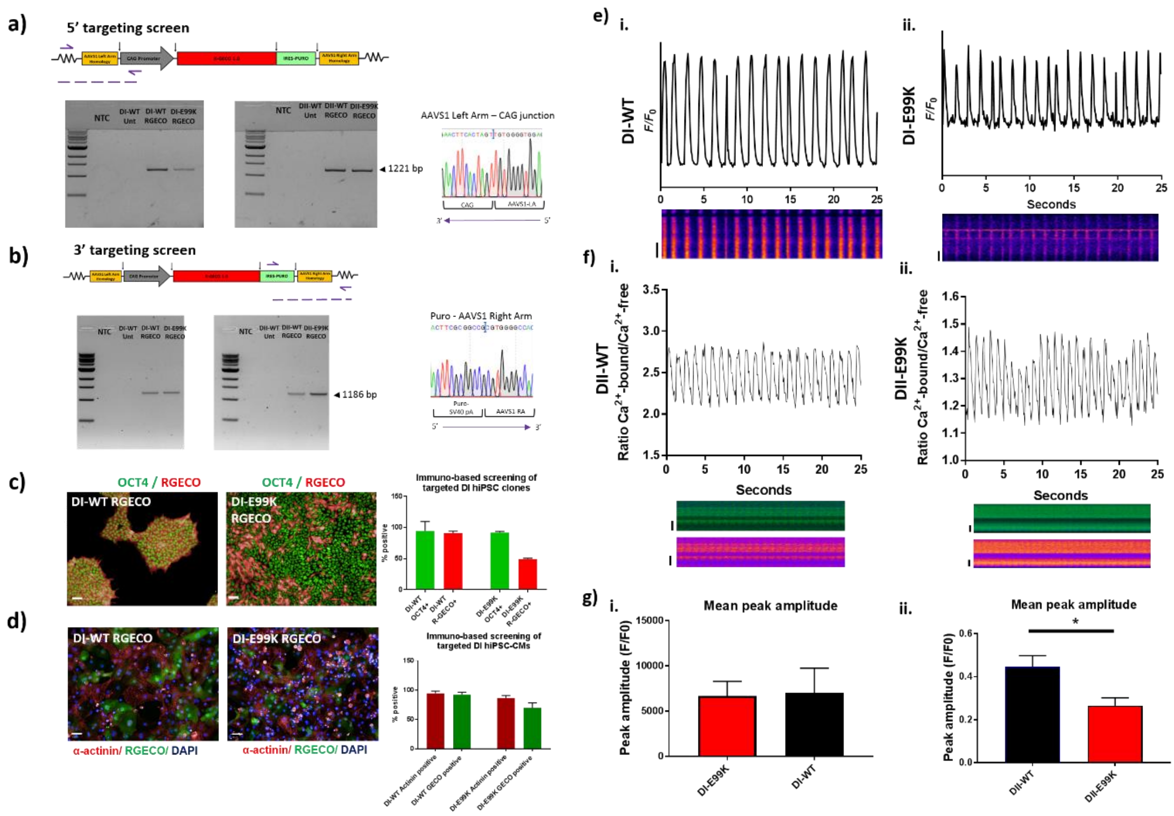

Figure S4. Characterization and arrhythmogenic event frequency of GECO-edited hiPSC lines. (a) Confirmatory 5' targeting PCR screen on gDNA isolated from E99K1 RGECO isogenic hiPSCs (left) and NC RGECO isogenic hiPSCs (right). Positive 5' targeting is indicated with a 1221bp product. These products were sequenced to show the junction between the AAVS1 left arm homology and the start of the CAG promoter. **(b)** Confirmatory 3' targeting PCR screen on gDNA isolated from from E99K1 RGECO isogenic hiPSCs (left) and NC RGECO isogenic hiPSCs (right). Positive 3' targeting is indicated with an 1186bp product. Sequencing of PCR products show the junction between the puromycin-SV40 pA sequence and the AAVS1 right arm. **(c)** E99K-Corr and E99K1 hiPSCs stained for the pluripotency marker OCT4 (green) and R-GECO (red). **(d)** E99K-Corr and E99K1 clones differentiated to hiPSC-CMs and stained for the cardiac marker α–actinin (red) and R-GECO (green). Scale bars = 50 µm. In **(e),** representative confocal line-scan traces and corresponding kymographs of spontaneous Ca^{2+} transients in E99K1 **(ei)** and E99K-Corr **(eii)** hiPSC-CMs utilising genetically encoded expression of R-GECO1.0 from the *AAVS1* locus. A line scan image was taken across a single cardiomyocyte every 75 milliseconds for 300 seconds. In **(f),** representative confocal line-scan traces and corresponding kymographs of spontaneous Ca^{2+} transients in NC **(fi)** and NC-Edit-E99K **(fii)** hiPSC-CMs utilising genetically encoded expression of ratiometric GEM-GECO from the *AAVS1* locus. **(g)** Mean peak amplitude analysis of Ca^{2+} transient events occurring during a 300 second line scan. Scale bars = 20 µm.

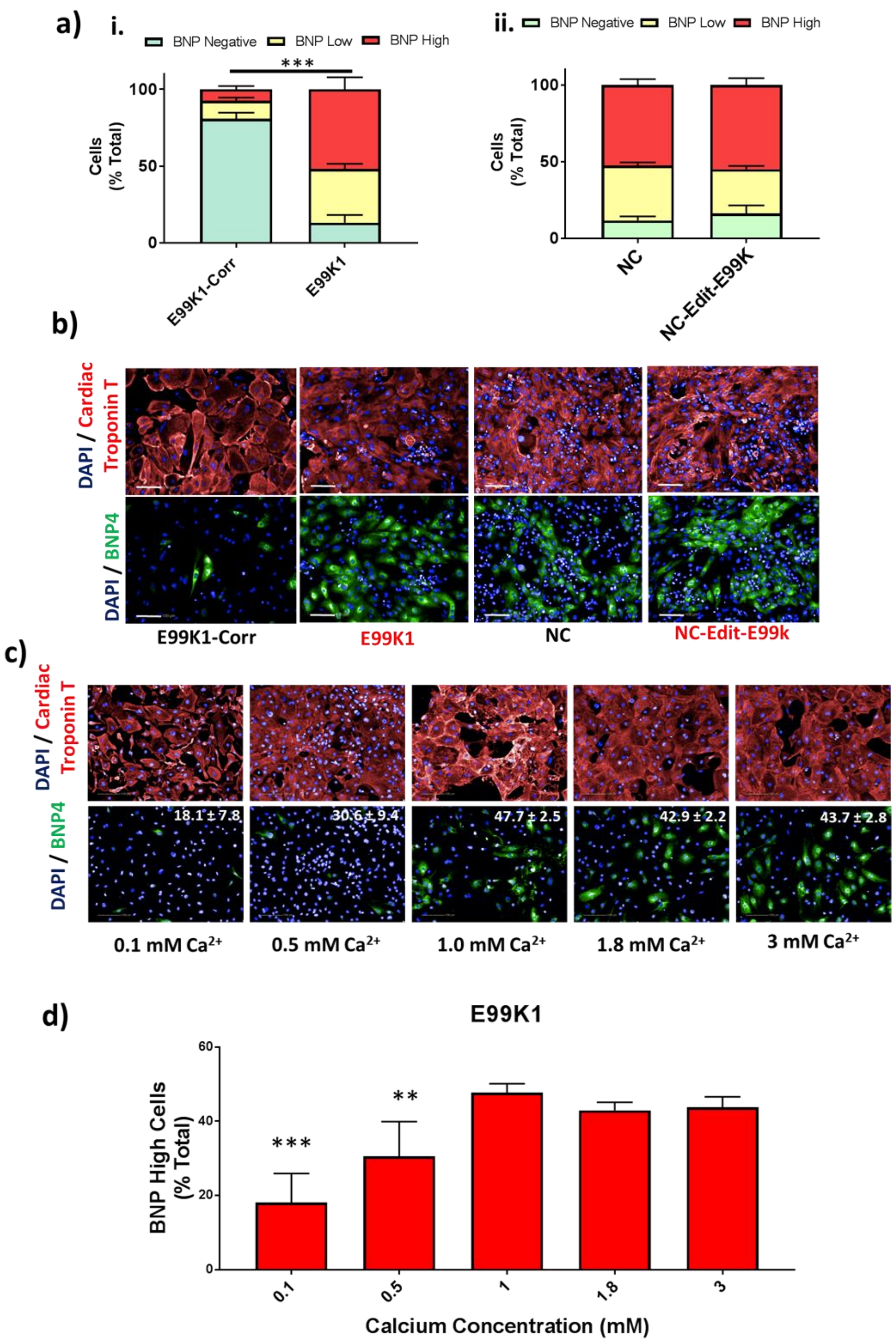

Figure S5. Hypertrophic BNP signalling in hiPSC-CMs. Quantification of BNP expression by an automated algorithm in gene-edited lines in comparison to their respective isogenic controls **(ai-ii)** from representative fluorescent micrographs **(b)** of BNP/cTnT/DAPI-immunostained hiPSC-CMs. E99K1 hiPSC-CMs were exposed to varied Ca^{2+} concentrations in Tyrode's solution for 24 hours and BNP/cTnT/DAPI-immunostained **(c)** and the percentage of highly-expressing BNP hiPSC-CMs quantified **(d)**. n = 3 E99K1, 3 E99K1-Corr, 3 NC, 3 NC-Edit-E99K. Scale bar =100 µm. n = 3. All error bars represent standard deviation. Significance was determined by students t-test, where: ** = p<0.01, *** = p<0.001.

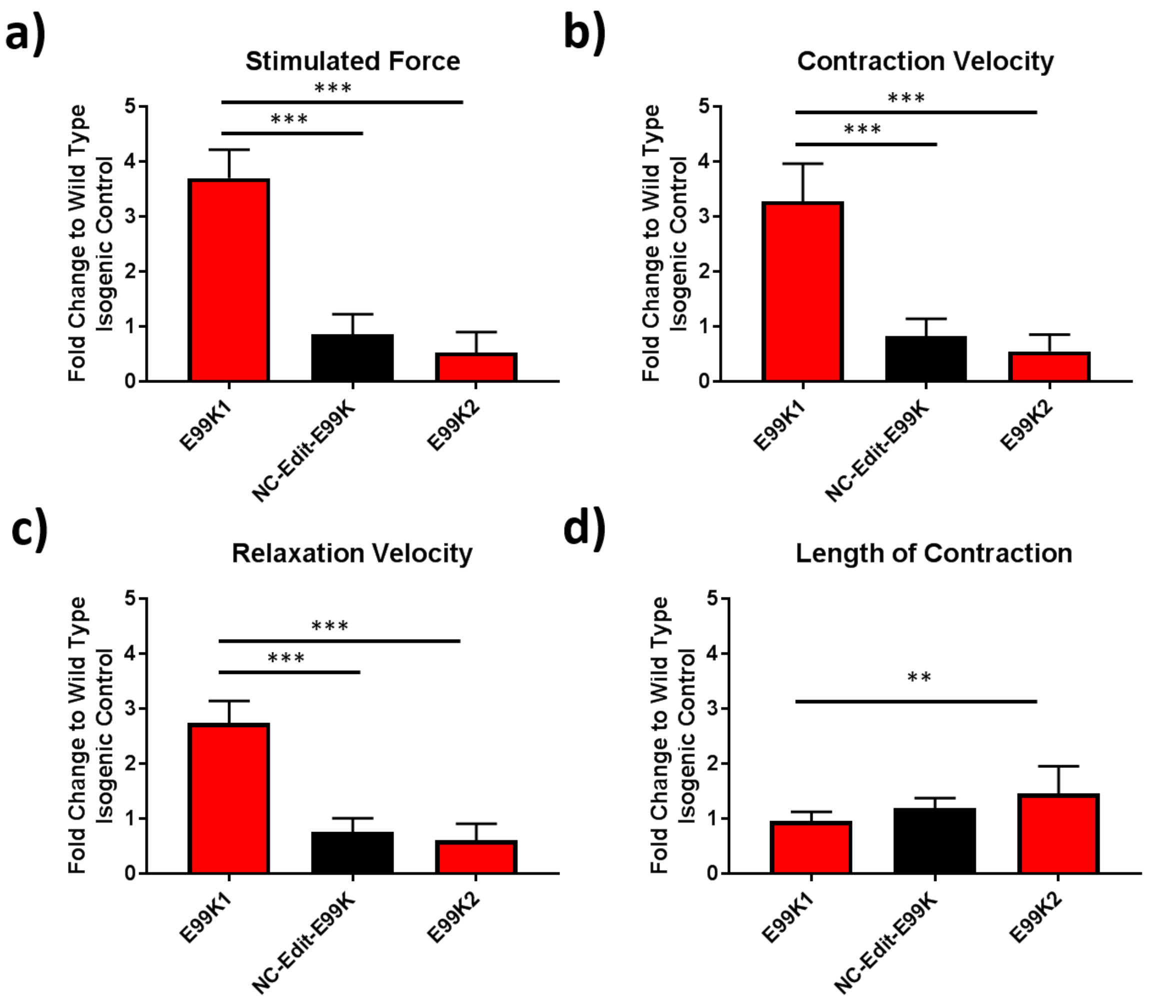

Figure S6. Normalised stimulated contraction of mutant hiPSC-CM EHTs. Auxotonic EHT contractions were recorded at 100f.p.s under stimulated conditions (1Hz). In (a) stimulated contraction force, (b) contraction velocity, (c) relaxation velocity, (d) length of contraction are shown respectively and normalised by fold change to healthy isogenic control lines. All error bars represent standard error of the mean. Significance was determined by one-way ANOVA, where: ** = $p<0.01$, and *** = $p<0.001$. n = 10 E99K1, 11 NC-Edit-E99K, 5 E99K2. Red, mutant ACTC E99K, black wild-type.

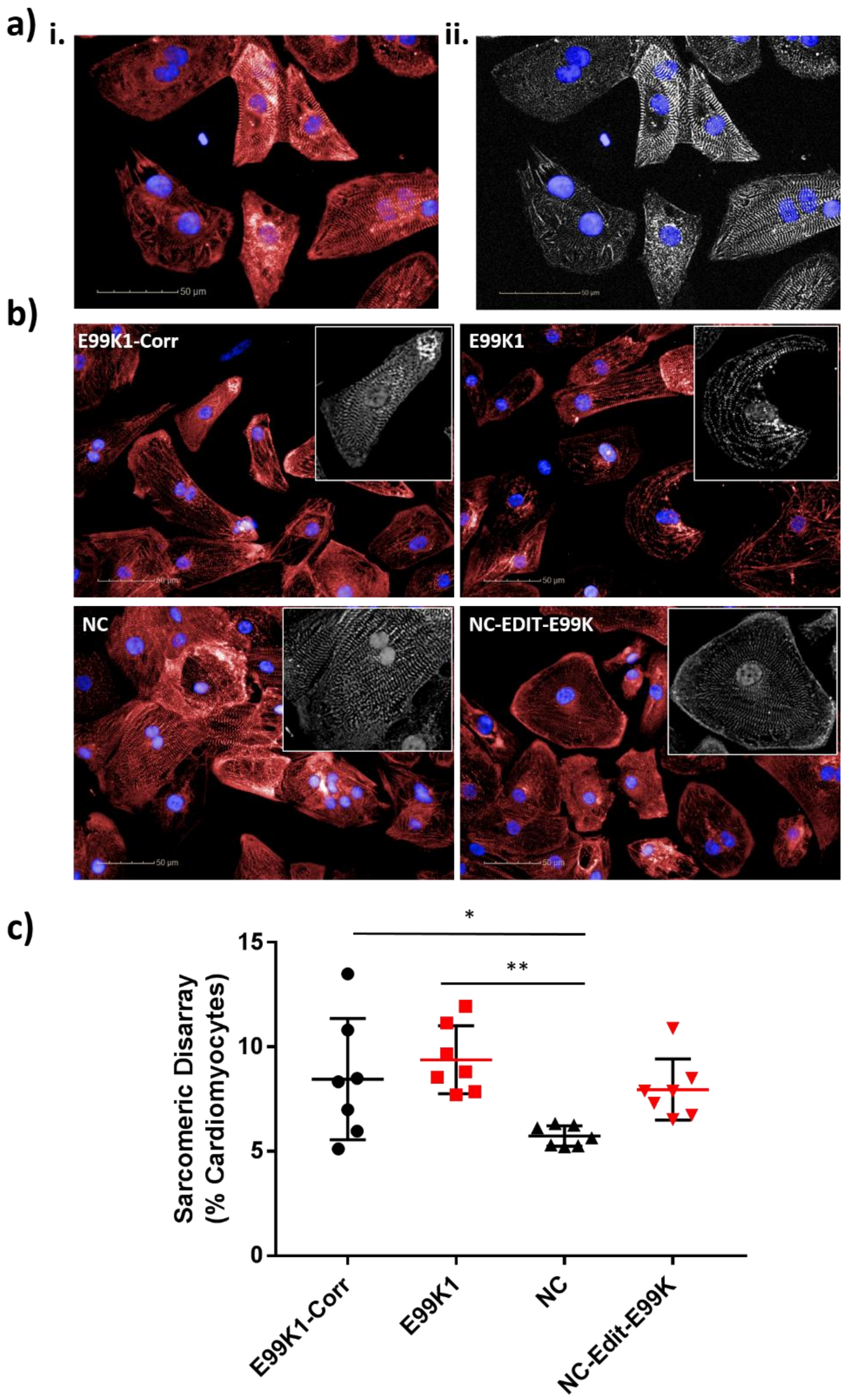

Figure S7. Sarcomeric disarray of hiPSC-cardiomyocytes. (ai) Images of human pluripotent stem cell-cardiomyocytes immunostained for sarcomeric banding (red is a-actinin staining; blue is DAPI), were sharpened using a mathematical correction (sliding parabola) to achieve higher signal resolution for alignment analysis **(aii)**. Sarcomeric disarray in isogenic pairs of hiPSC-CMs was imaged **(b)** and quantified **(c)** through analysis of morphological and texture properties in a PhenoLOGIC™ machine learning imaging algorithm (adapted from PerkinElmer). n=7. Average cells per n analyzed for E99K1 (4187), E99K1-Corr (2938), NC (3961), and NC-Edit-E99K (3072). Scale bar =50µm. Significance was determined by ANOVA, where: * = $p<0.05$, ** = $p<0.01$.

Supplementary Experimental Procedures

Fibroblast isolation

Fibroblasts were released from skin punch biopsies through manual dissection of tissue into 1mm pieces, and enzymatic digestion at 37 ^{0}C with 2.5% trypsin Trypsin-EDTA (LifeTechnologies #10462502) for 20 minutes, followed by 1 mg/ml collagenase IV (LifeTechnologies #17104019) for 90 minutes. Released fibroblasts were centrifuged at 200xg for 5 minutes, and resuspended in a fibroblast growth medium consisting of DMEM basal medium (Gibco #11965092) supplemented with 20% heat-inactivated fetal calf-serum (FCS, Biochrom #S0615), 1% Non-Essential Amino Acids (NEAA, Gibco #11140050), 1% GlutaMAX (Gibco#35050061), 100 µM β-mercaptoethanol (Sigma #63689) and 1% penicillin/ streptomycin (PEST, Gibco).

hiPSC culture

All cell culture experiments were performed in a type II Biological Safety Cabinet, and cells were incubated in a humidified incubator at 37 °C and 5% CO_2. hiPSCs were routinely maintained in E8 medium on 1:100 Matrigel (Corning #356235)-coated plastic ware (Nunc). Cells were passaged every 3 days by washing once with Ca^{2+}/ Mg^{2+}-free Phosphate Buffer Saline (PBS, Gibco #14190-094), followed by incubation with TrypLE for 6 minutes. Thereafter, hiPSC were resuspended in E8 supplemented with 10 µM Y-27632 (ROCKi, Tocris Bioscience #1254/10) and seeded into new Matrigel-coated flasks at approximately 20000 cells/ cm^2. Medium was changed every day and cell lines were used between passages 20-30.

hiPSC-CM dissociation

hiPSC-CMs generated by the monolayer differentiation method were dissociated using a Collagenase II-based protocol, as previously described (Breckwoldt et al. 2017). Briefly, cells were washed twice with Ca^{2+}/Mg^{2+}-free Hank`s Balanced Salt Solution (HBSS, LifeTechnologies #14175095). Subsequently, cardiomyocytes were incubated with 200U/ml Collagenase II (Worthington #LS004176) in Ca^{2+}/Mg^{2+}-free HBSS, supplemented with 1mM HEPES (Sigma

#H4034), 10 µM Y-27632 and 30nM N-Benzyl-p-toluenesulfonamide (BTS, TCI #B3082), for 3.5 h at 37 °C at 5% CO_2. Thereafter, dissociated cardiomyocytes were collected from the flasks and washed with RPMI supplemented with 24 µg/ml Deoxyribonuclease II (DNAseII – Sigma #D8764), followed by centrifugation at 100 xg for 15 min. Subsequently, cells were resuspended in warm RPMI and pipetted slowly through a 100 µm cell strainer (VWR # 89508-840) into a tube. Cells were then counted using an automated CEDEX HiRes counter (Roche) and centrifuged again as above. Afterwards, cardiomyocytes were resuspended in RPMI+B27+INS for seeding into Vitronectin-N (VN, Lifetech #A14700)-coated vessels.

Confocal line scans of targeted GECI hiPSC-CM Ca^{2+} transients

Targeted GECI hiPSC-CMs were generated as previously described (Mosqueira et al. 2018), cultured in RPMI+B27+INS without phenol red (Gibco #11835030) and dissociated on day 15. hiPSC-CMs were seeded at a density of 150,000 cells per well in VN-coated 35mm-diameter MatTek dishes (Nunc). CMs were assayed no earlier than day 24 and no later than day 30 of differentiation. Intracellular Ca^{2+} transient measurements were made using an LSM 880C confocal microscope (Carl Zeiss) in the line-scan mode, as previously described (Yazawa et al. 2011). Briefly, CMs were located using a 40x oil objective and a longitudinal line was drawn across a single CM. Line-scan images were taken every 75 milliseconds for a total of 4000 cycles resulting in a 5 minute scan. CMs were kept at 37 °C and 5% CO_2 throughout data acquisition. Confocal line scan images were analysed in Fiji software (National Institute of Health). The average fluorescence intensity of each line was calculated over time to generate a confocal line-scan trace. Using the multi kymograph Fiji plugin, a corresponding kymograph image was produced. In order to calculate beat rate and arrhythmic events, data was fed into pClamp software (Molecular Devices). Baselines were adjusted to account for photobleaching, and Ca^{2+} transients were counted and analysed using the 'event detection' function. In order to determine abnormal delayed after depolarization DAD-like Ca^{2+} transients, median peak height analysis was performed using Excel (Microsoft), wherein the median peak height for a line-scan image was calculated, and any Ca2+ transient events that were below 75

% of the peak height were considered ‘abnormal’, including those that did not return to baseline and gave a ‘double peak’.

BNP assay

BNP assay was performed as previously described (Carlson et al. 2013). In brief, dissociated hiPSC-CMs were seeded at 100,000 cells/ cm^2 in VN-coated 96 well plates (CellCarrier, Perkin Elmer). One week later, cells were incubated with either Tyrode’s solution with varied Ca^{2+} concentrations, or RPMI+B27+INS with or without 10 µM ranolazine (SelleckChem #1425) or dantrolene (Cayman Chemicals # 14326) for 15 h, after which 1 µg/ml Brefeldin A (Sigma #B7651) was added to the medium and incubated for another 3 h, at 37 °C and 5 % CO_2. Thereafter, cells were fixed and immunostained as described below. Image acquisition was performed as described below and BNP signal intensity was determined in the perinuclear region of cardiomyocytes, and divided into high, medium and low/negative according to pre-established empirical thresholds.

Immunocytochemistry (ICC) and image acquisition

Dissociated hiPSC-CMs or hiPSC were cultured in VN- or Matrigel-coated 96-well plates (CellCarrier, Perkin Elmer #6005550) respectively, at approximately 50,000 cells/cm^2 as described above. Cells were washed with PBS and fixed in 4% Paraformaldehyde (PFA, Sigma) at RT for 15 min. Afterwards, cells were washed in 0.1% Tween-20 (Fisher Scientific) in PBS, permeabilized with 0.1% Triton-X (Sigma) in PBS for 30 min at RT, and incubated with 4% goat serum (Sigma) in PBS (blocking solution) for 1h at RT, to prevent unspecific antibody binding. Subsequently, primary antibody incubation was performed overnight at 4 °C in blocking solution, at the following dilutions: anti-OCT4-1:200 (Santa cruz biotech #sc-5279), anti-α-actinin-1:800 (Sigma #A7811), anti-TroponinT-1:500 (Abcam #45932), anti-E99K-ACTC1: 1:800 (OriGene Technologies #AP54763PU-N), (Rowlands et al. 2017), anti-ProBNP4-1:500 (Abcam #13115). Thereafter, samples were washed 3 times with 0.1% Tween20 in PBS and incubated with Alexa Fluor secondary antibodies (Life Technologies) 1:400 in blocking solution for 1h at RT. Afterwards, cells were washed with 0.1% Tween-20 in PBS for (3x 5min), followed by nuclei counterstaining with 0.5

µg/ml DAPI (Sigma #D9542) in PBS, respectively, for 15 min at RT. Samples were subsequently washed and stored at 4 °C in PBS until automated image acquisition was performed in the Operetta high-content imaging system (Perkin Elmer) and analysed using Harmony high-content imaging analysis software.

CellOPTIQ analysis

The CellOPTIQ platform (Hortigon-Vinagre et al. 2016) was used to record optical-based Ca^{2+} transients from hiPSC-CMs. Cells were seeded into Matrigel-coated 96-well plates at a density of 50,000 cells per well. These hiPSC-CMs were then incubated at 37 °C and 5% CO_2 for 48 h to allow cells to recover. To image the Ca^{2+} handling properties of the hiPSC-CMs, they were loaded with Fluo4-AM (LifeTechnologies #F14201). Cells were incubated in RPMI media containing 10 µM Fluo4-AM at 37°C and 5% CO_2 for 30 min. After the 30-min incubation, the medium was removed and the hiPSC-CMs washed once before 100 µL of fresh medium was added to wells. These plates were then incubated at 37 °C and 5% CO_2 for 15 min to allow the hiPSC-CMs to equilibrate before recording traces. Data were analysed using CellOPTIQ proprietary software of Clyde Biosciences and were normalized to a maximum amplitude of 1 and minimum of 0 to standardize height for comparison of traces created in Origin software package.

Real-time qPCR

RNA was extracted using the RNeasy mini kit (Qiagen), and cDNA synthesis performed using Superscript III (Life Technologies) following manufacturer's instructions. Real-time qPCR reactions were performed using TaqMan® Gene Expression Assays (Applied Biosystems) following manufacturer's instructions. Briefly, reactions were performed in MicroAmp Fast 96 well plates (#4346907) and contained 2x Taqman® gene expression mastermix (#4369016) and the relevant TaqMan® gene expression assay (CASQ2-Hs00154286_m1, CALM1-Hs00300085_s1, CAMK2D-Hs00943538_m1, PPP3cA-Hs00174223_m1, PPP3cB-Hs00236113_m1, IRF8-Hs00175238_m1, PLN-Hs01848144_s1, CAPN1-Hs00559804_m1, CACNA1C-Hs00167681_m1). Amplification was performed in ABI 7500 Real-Time PCR system (Applied Biosystems). Normalisation was performed

using the cardiac gene TNNT2 and 18S as reference genes, as previously described (Burkart et al. 2016), and the WT isogenic cardiomyocytes lines were used to calculate relative expression using the 2-(ΔΔCT) method (Schmittgen and Livak 2008).
