# Supplementary material for: Isogenic Pairs of hiPSC-CMs with Hypertrophic Cardiomyopathy/LVNC-Associated ACTC1 E99K Mutation Unveil Differential Functional Deficits
Source: Stem Cell Reports. 2018 Nov 1;11(5):1226–43. doi: 10.1016/j.stemcr.2018.10.006 (PMC6235010; doi:10.1016/j.stemcr.2018.10.006)
Supplement: Document S2. Article plus Supplemental Information [file mmc2.pdf]

Stem Cell Reports
Article

OPEN ACCESS

Isogenic Pairs of hiPSC-CMs with Hypertrophic Cardiomyopathy/LVNC-Associated ACTC1 E99K Mutation Unveil Differential Functional Deficits

James G.W. Smith,[1,7,10,*] Thomas Owen,[2,10] Jamie R. Bhagwan,[1] Diogo Mosqueira,[1] Elizabeth Scott,[1] Ingra Mannhardt,[3,8] Asha Patel,[1,6] Roberto Barriales-Villa,[4,5] Lorenzo Monserrat,[5,9] Arne Hansen,[3,8] Thomas Eschenhagen,[3,8] Sian E. Harding,[2] Steve Marston,[2] and Chris Denning[1,*]

[1]Wolfson Centre for Stem Cells, Tissue Engineering and Modelling, Centre for Biomolecular Sciences, University of Nottingham, University Park, Nottingham NG7 2RD, UK
[2]National Heart and Lung Institute, Imperial College, London W12 0NN, UK
[3]Institute of Experimental Pharmacology and Toxicology, University Medical Centre, Hamburg-Eppendorf, Hamburg, Germany
[4]Inherited Cardiovascular Diseases Unit, Cardiology Service, Complexo Hospitalario Universitario A Coruña, Servizo Galego de Saúde (SERGAS), Universidade da Coruña, A Coruña, Spain
[5]Instituto de Investigación Biomédica de A Coruña (INIBIC), A Coruña, Spain
[6]Department of Gene Therapy, National Heart and Lung Institute, Imperial College London SW3 6LR, UK
[7]Faculty of Medicine and Health Sciences, Norwich Medical School, University of East Anglia, Norwich Research Park, Norwich NR4 7UQ, UK
[8]DZHK (German Centre for Cardiovascular Research), Partner Site Hamburg/Kiel/Lübeck, Hamburg, Germany
[9]Health in Code S.L., Cardiology Department, A Coruña, Spain
[10]Co-first author
*Correspondence: james.smith@nottingham.ac.uk (J.G.W.S.), chris.denning@nottingham.ac.uk (C.D.)
https://doi.org/10.1016/j.stemcr.2018.10.006

SUMMARY

Hypertrophic cardiomyopathy (HCM) is a primary disorder of contractility in heart muscle. To gain mechanistic insight and guide pharmacological rescue, this study models HCM using isogenic pairs of human induced pluripotent stem cell-derived cardiomyocytes (hiPSC-CMs) carrying the E99K-ACTC1 cardiac actin mutation. In both 3D engineered heart tissues and 2D monolayers, arrhythmogenesis was evident in all E99K-ACTC1 hiPSC-CMs. Aberrant phenotypes were most common in hiPSC-CMs produced from the heterozygote father. Unexpectedly, pathological phenotypes were less evident in E99K-expressing hiPSC-CMs from the two sons. Mechanistic insight from Ca^{2+} handling expression studies prompted pharmacological rescue experiments, wherein dual dantroline/ranolazine treatment was most effective. Our data are consistent with E99K mutant protein being a central cause of HCM but the three-way interaction between the primary genetic lesion, background (epi)genetics, and donor patient age may influence the pathogenic phenotype. This illustrates the value of isogenic hiPSC-CMs in genotype-phenotype correlations.

INTRODUCTION

Cardiomyopathies are defined as primary disorders of contractility in heart muscle. Most classifications of cardiomyopathy divide the disease into acquired and inherited disease due to a mutation and into hypocontractile phenotype with reduced ejection fraction or hypercontractile phenotype with preserved ejection fraction (Maron et al., 2006). Hypertrophic cardiomyopathy (HCM) is a common clinical phenotype, found in up to 1 in 500 of the general population, and symptoms usually manifest in the second or third decade of life. HCM is characterized by thickened left ventricular walls, notably the interventricular septum, and a hyperdynamic myocardium with defective relaxation, with an enhanced susceptibility to arrhythmia that can lead to sudden death in a small proportion of patients. In the longer term, these abnormalities can lead to heart failure. Histologically HCM leads to myocyte disarray and interstitial fibrosis.

HCM is overwhelmingly an inherited disease, and in most cases mutations in one of the genes coding for the contractile and structural proteins of the cardiac muscle sarcomere is responsible (Marston, 2011). Understanding phenotype-genotype relations in HCM is complex. Several other inherited diseases, including restrictive cardiomyopathy and left ventricular non-compaction (LVNC), appear to be subsets of HCM and can share the same mutations. Moreover, the penetrance of the HCM phenotype is variable, since symptoms of carriers of HCM-linked mutations can range from asymptomatic to fatal cardiac dysfunction (Sedaghat-Hamedani et al., 2018). Insight into the biophysical mechanism behind cardiomyopathies has advanced significantly. It is now clear that an enhanced myofilament Ca^{2+} sensitivity is often the primary consequence of HCM mutations leading to the hyperdynamic phenotype (Spudich, 2014) and the enhanced susceptibility to arrhythmia (Huke and Knollmann, 2010). Yet the pathway to secondary HCM phenotypes, such as myocyte hypertrophy, myocardial disarray, and interstitial fibrosis, are not easily explained by sarcomeric gene defects alone and may be dependent upon genetic background.

Actin comprises the main component of the sarcomeric thin filament, with α-cardiac actin (encoded by *ACTC1*;

This is an open access article under the CC BY license (http://creativecommons.org/licenses/by/4.0/).

Genbank: NM_005159) accounting for ~80% of actin expression in mature cardiomyocytes (Olson et al., 1998). To date, 14 *ACTC1* mutations have been identified in patients diagnosed with one or more forms of cardiomyopathy, of which 11 are associated with HCM (Mogensen et al., 1999; Olson et al., 1998, 2000; Morita et al., 2008; Kaski et al., 2009; Olivotto et al., 2008). The most extensively studied of these mutations is c.G301A (p.Glu101Lys). Although this mutation occurs at codon 101 of the *ACTC1* gene, the mature protein in muscle has the amino acid substitution E99K due to the removal of two N-terminal amino acids during post-translational processing (Rubenstein and Martin, 1983).

Although mutations in *ACTC1* are relatively rare, the clinical consequences have been studied unusually thoroughly. Three clinical studies have all shown the E99K-ACTC1 mutation to present varying HCM-related phenotypes (Arad et al., 2002; Olson et al., 2000), with the most extensive study by Monserrat et al. (2007). This found diverse and overlapping E99K-ACTC1 phenotypes using clinical and morphological data from 94 cases. There were clear differences in the nature and severity of the clinical expression of the disease between carriers, but none was normal. HCM and LVNC were the most frequently reported phenotypes (Monserrat et al., 2007; Song et al., 2011). Hypertrophy was reported from 62 of 76 mutation carriers. The distribution of the hypertrophy was predominantly apical. Electrocardiography (ECG) investigation showed abnormalities in 53 of 61 carriers. Atrial fibrillation or flutter was found in 7 of 53. The 22 adverse events reported included eight sudden deaths (five in a single family).

Several different E99K-ACTC1 models have been developed to investigate the mechanisms behind these disease phenotypes. These include *in vitro* motility and laser trap assays (Debold et al., 2010), and reconstitution of human E99K-ACTC1 in the thin filament of bovine cardiac muscle fibers (Bai et al., 2015). Studies in transgenic E99K-ACTC1 mice are the most comprehensive (Song et al., 2011). These models have been useful in obtaining data on the properties of E99K-ACTC1, with increased Ca^{2+} sensitivity often reported for the mutant protein (Song et al., 2011, 2013; Bai et al., 2015), although this is sometimes not observed and adds to the debate surrounding disease mechanism (Debold et al., 2010).

Nevertheless, while the α-cardiac actin sequence is identical in human and mouse, conclusions are still limited by the predominance of the α-myosin heavy chain isoform in the ventricles of mice versus the β isoform in humans. Differing levels of mutant sarcomeric transcripts than those occurring from a single mutant allele in human expression, and intrinsic differences in cardiac function between the species, including a 10-fold higher heart rate compared with humans, are important (Arad et al., 2002; Denning et al., 2016). There is need for a fully human *in vitro* E99K-ACTC1 model, in which protein interaction data can be more readily related to the patient phenotype and used to explain varied disease penetrance.

Here, we developed a human model of HCM by harnessing human induced pluripotent stem cell (hiPSC) reprogramming and CRISPR/Cas9 genome editing technology. By generating hiPSC lines from patients carrying the E99K mutation (E99K1 and E99K2) and a healthy non-carrier relative (NC) and then using CRISPR/Cas9 technology, we created a model whereby the mutation had been corrected in two isogenic pairs and introduced in the other. This model recapitulated many disease phenotypes including abnormal contractility, Ca^{2+} sensitivity/handling, arrhythmogenesis, and hypertrophic signaling. In almost all cases the aberrant phenotypes observed in E99K-expressing hiPSC-derived cardiomyocytes (hiPSC-CMs) were greater than in non-expressing isogenic counterparts, consistent with this mutant protein having a central role in HCM.

However, there were considerable variations in severity between the lines, most notably with hiPSC-CM phenotypes from the heterozygote father being most pronounced and widespread. This included contraction force and velocity, and relaxation time; hypersensitivity to low Ca^{2+}; Ca^{2+} handling (contraction and relaxation); hypertrophic brain natriuretic peptide (BNP) expression; and expression of Ca^{2+} handling machinery (*CASQ2*, *CALM1*, *CAMK2D*, *PPP3cA*). Although we had not expected this, it may be attributed to the greater age of the father relative to his sons. Nevertheless, the E99K mutation perturbed Ca^{2+} signaling pathways in some instances, which enabled hypothesis-driven drug rescue experiments. Thus the Ca^{2+} handling modifier drugs, ranolazine and dantrolene, reduced hypertrophic signaling, with dual treatment being most effective.

RESULTS

Generation of Isogenic Sets of E99K-ACTC1 hiPSCs by CRISPR/Cas9 Editing

Skin-punch biopsies were obtained and reprogrammed to hiPSCs from three donors of the same family; a 48-year-old male patient carrying the c.$ACTC1^{G301A}$ mutation (E99K1), the 14-year-old healthy c.$ACTC1^{G301G}$ son (NC), and the 19-year-old son carrying the c.$ACTC1^{G301A}$ mutation (E99K2) (Figure S1). The resulting hiPSC lines expressed the OCT4 pluripotency marker (Figure 1A) and had the expected heterozygote mutant (E99K1; E99K2) or wild-type (NC) *ACTC1* sequences (Figure 1B).

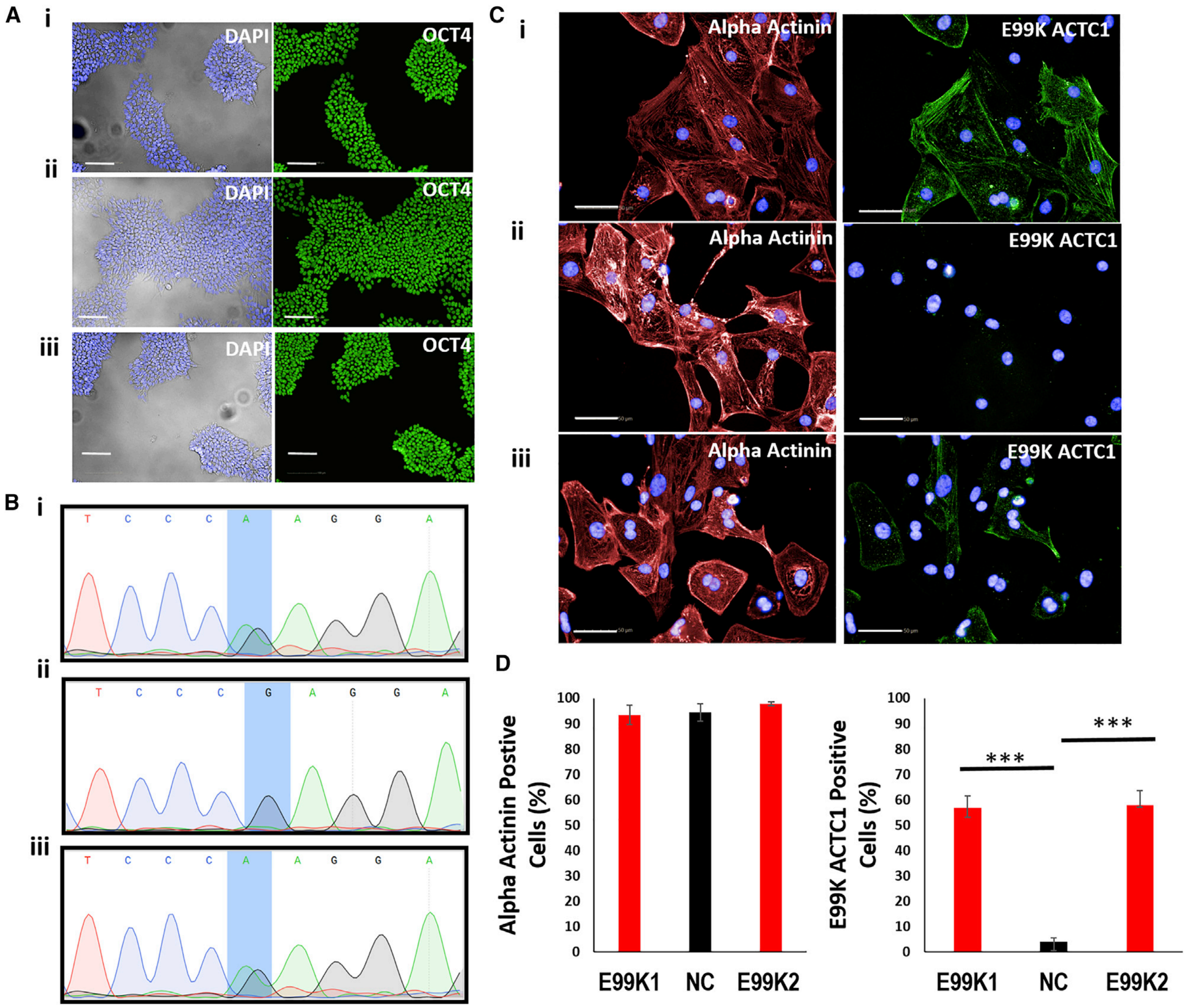

Figure 1. Production of hiPSC Cardiomyocytes with or without c.301G>A E99K Mutations

(A) Relatives with (E99K1, E99K2) or without (NC) the mutation donated skin biopsies. Fibroblasts were reprogrammed into hiPSC using non-integrating CytoTune 2.0 Sendai virus and E6/E8 culture medium and stained for pluripotency marker, OCT4 (green; blue is DAPI).
(B–D) In (B), sequencing confirmed the (C)301G>A ACTC1 mutation that causes the E99K polymorphic variant in diseased, but not healthy, lines. In (C), high-efficiency differentiation of hiPSC yielded cardiomyocyte purities of >90% (red is α-actinin staining; blue is DAPI). Counterstaining (green) with an antibody specific to the mutant E99K peptide showed that ~50% of the diseased cardiomyocytes were positive, suggesting biallelic expression of both mutant and healthy alleles (C and D). In contrast, E99K reactivity was not detected in hiPSC cardiomyocytes from the healthy individual (C and D). n = 6.
Scale bars, 100 μm (A) and 50 μm (C). Significance was determined by Student's t test, ***p < 0.001.

Differentiation to high purity (>90% α-actinin$^+$) cardiomyocytes (hiPSC-CMs) (Figures 1C and 1D) allowed reactivity to an antibody specific to the mutant E99K peptide to be tested. This showed positive protein expression in ~50% of the E99K1 and E99K2 hiPSC-CMs, suggesting that only one allele of the *ACTC1* gene is active in any given cell. As expected, E99K peptide reactivity was not detected in NC hiPSC-CMs (Figures 1C and 1D).

Isogenic controls were generated for E99K1, NC, and E99K2 hiPSCs using a footprint-free *PiggyBac* based CRISPR/Cas9strategy, coupled with PCR screening and sequence confirmation (Kondrashov et al., 2018) (Figure 2A). This identified targeted clones for E99K1 and E99K2 where the c.301G>A *ACTC1* mutation had been corrected (producing E99K1-Corr and E99K2-Corr) and introduced into one allele of NC (producing NC-Edit-E99K)

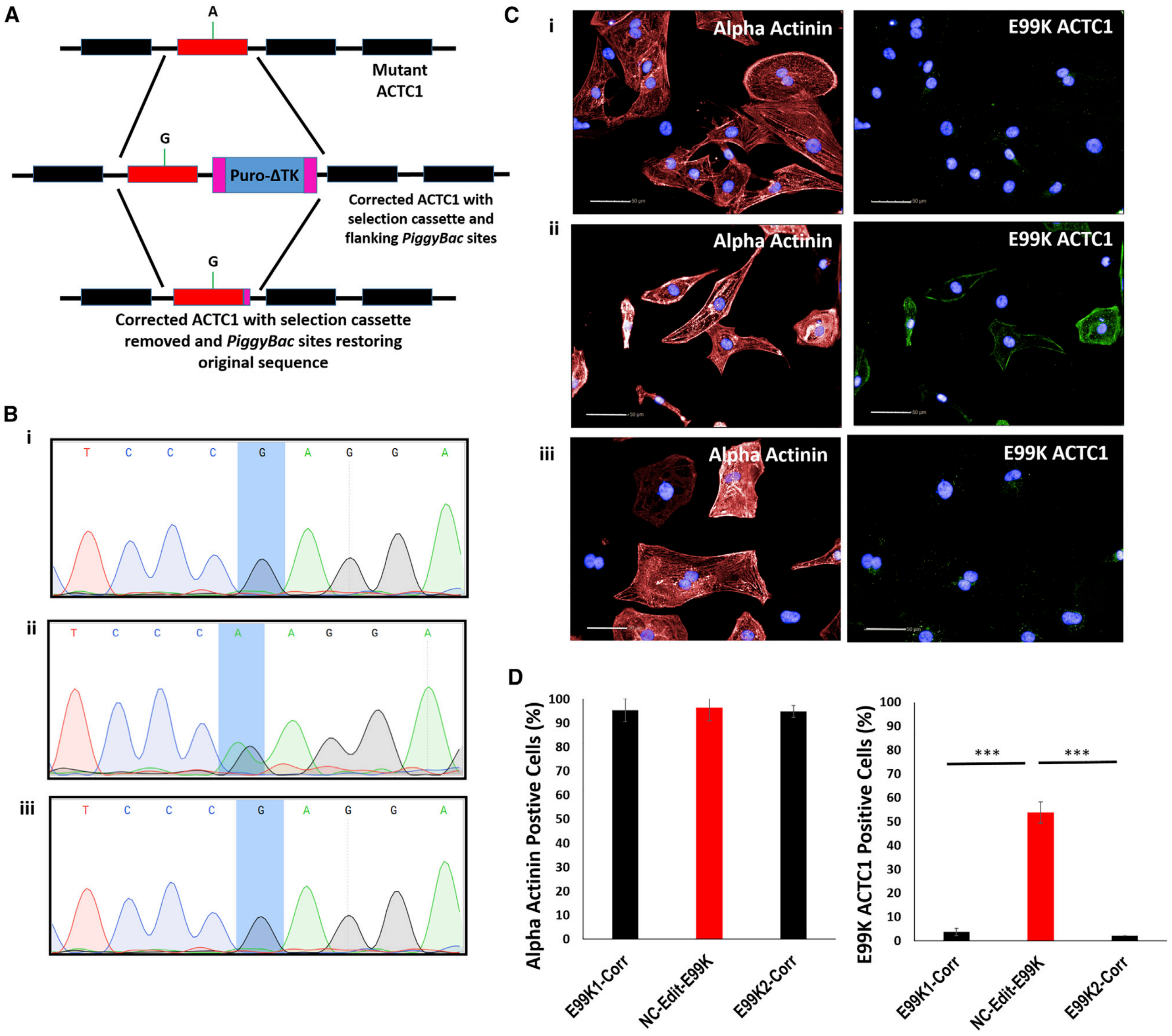

Figure 2. Generation of Isogenic E99K hiPSC Cardiomyocytes

(A) A footprint-free *PiggyBac* targeting strategy of the ACTC1 gene was used to correct or induce E99K mutation into hiPSC lines.

(B–D) In (B), sequencing confirmed the (C)301G>A ACTC1 mutation had been corrected in E99K1 and E99K2 and introduced in NC. In (C), high-efficiency differentiation of hiPSC still yielded cardiomyocyte purities of >90% after gene editing (red is α-actinin staining; blue is DAPI). Counterstaining (green) with an antibody specific to the mutant E99K peptide confirmed protein expression was lost in diseased patients (C and D). In contrast, E99K reactivity was now detected in hiPSC cardiomyocytes from the healthy individual (C and D). n = 6. Scale bars, 50 μm.

Significance was determined by Student's t test, ***$p < 0.001$.

(Figure 2B). Analysis by PCR of six potential mismatched guide sequences indicated that no off-target events had occurred in other genes (Figure S2).

These clonal lines still yielded high purity (>90% α-actinin$^+$) hiPSC-CMs. However, E99K peptide reactivity was no longer detected in E99K1-Corr and E99K2-Corr, but was now present in ~50% of the NC-Edit-E99K hiPSC-CMs. This confirmed successful genomic editing correlated with altered protein isoform expression, generating isogenic control lines (E99K1-Corr, E99K2-Corr, and NC-Edit-E99K) (Figures 2C and 2D). Thus, a suite of three isogenic pairs comprising six hiPSC lines was successfully created: E99K1 and E99K1-Corr; NC and NC-Edit-E99K; and E99K2 and E99K2-Corr.

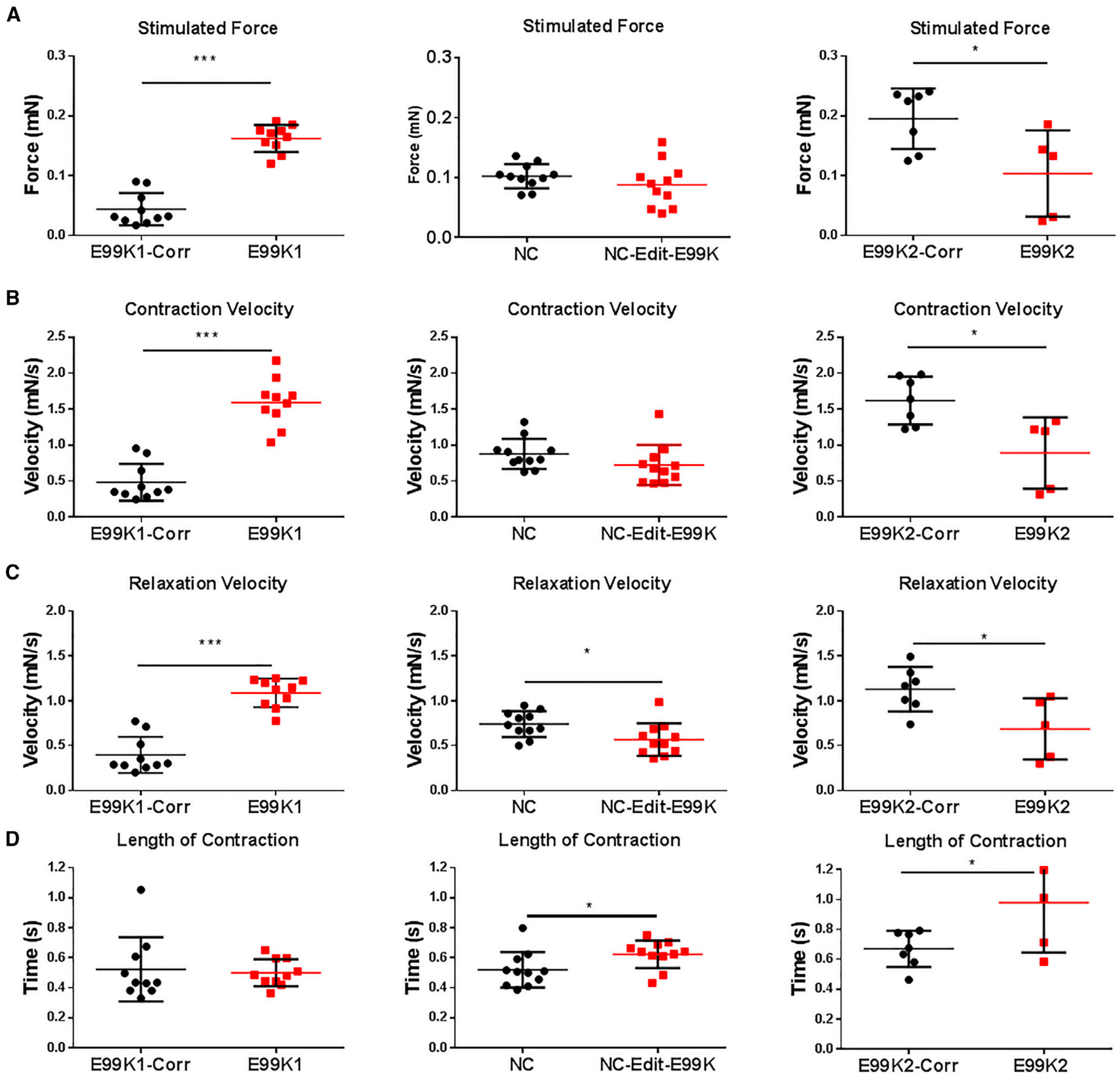

Figure 3. Stimulated Contraction of Isogenic Pairs of hiPSC-CM EHTs
Auxotonic EHT contractions were recorded at 100 frames per second under stimulated conditions (1 Hz). Stimulated contraction force (A), contraction velocity (B), relaxation velocity (C), and length of contraction (D) are shown. All error bars represent SEM. Significance was determined by Student's t test, *$p < 0.05$ and ***$p < 0.001$. n = 10 E99K1, 10 E99K1-Corr, 11 NC-Edit-E99K, 11 NC, 5 E99K2, and 7 E99K2-Corr. Red, mutant ACTC E99K; black, wild-type. See also Figure S6.

Contractility in Wild-Type and E99K EHT

hiPSC-CMs were successfully encapsulated into human engineered heart tissues (hEHTs), matured for 2 weeks, and stimulated at 1 Hz. We used image analysis of video microscopy for the calculation of contraction parameters as previously described (Eschenhagen et al., 2012). E99K1 had significantly stronger contractions than E99K1-Corr (Figure 3A); however; there was no significant difference between NC and NC-Edit-E99K lines, and E99K2 generated significantly less force than its corrected control.

Although the E99K1 line generated significantly more force, the overall length of contraction time was similar to E99K1-Corr. However, both NC-Edit-E99K and E99K2 lines had significantly longer contraction times than their

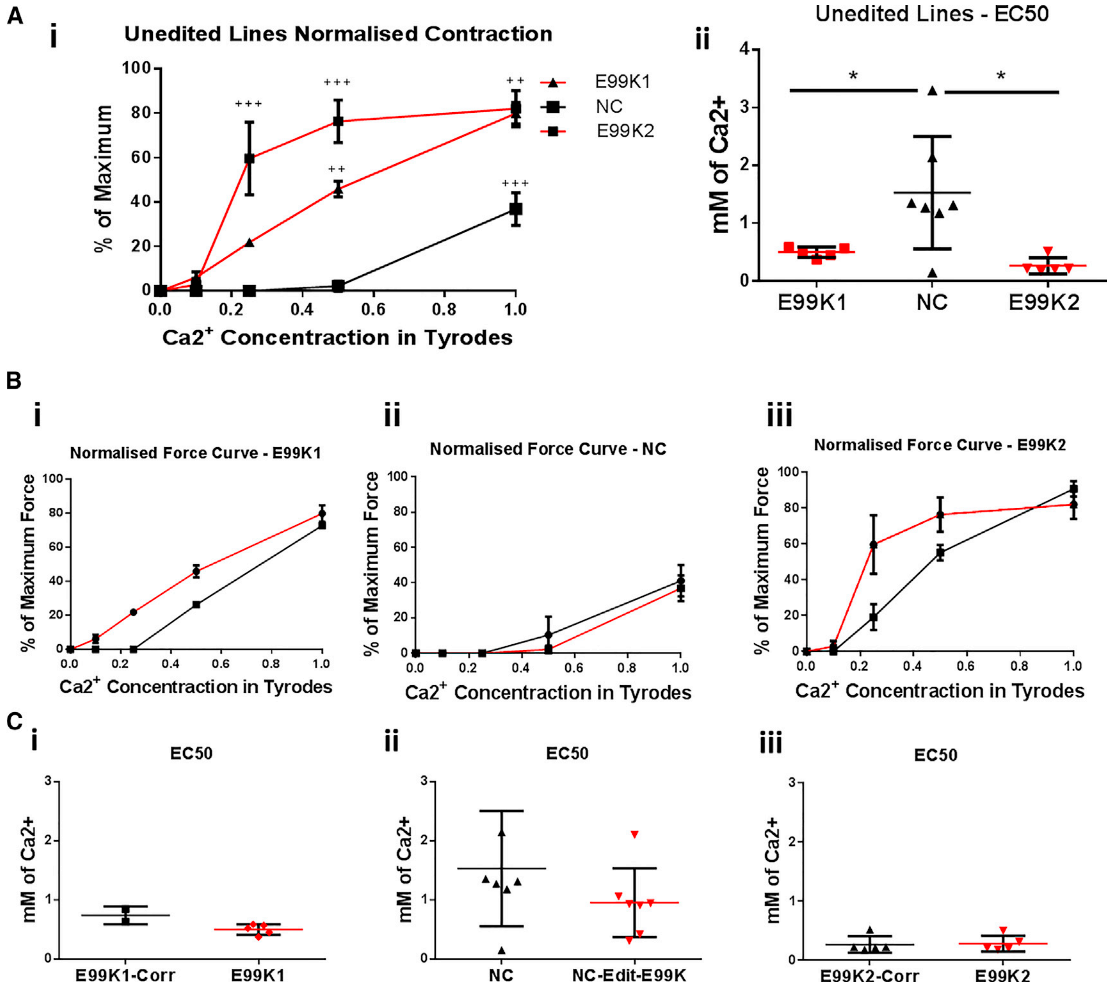

Figure 4. Ca^{2+} Sensitivity of Isogenic Pairs of hiPSC-CM EHTs

iPSC-CM EHT were exposed to increasing concentrations of Ca^{2+} in Tyrode's solution and contractions were recorded while being stimulated at 1 Hz. In (Ai), the force of EHTs was calculated as a percentage of maximum for the unedited lines, with E99K lines shown in red and wild-type in black. EC_{50} values were calculated using absolute force and are shown for the unedited lines in (Aii). The comparison of normalized forces are shown for the isogenic pairs of E99K1 (Bi), NC (Bii), and E99K2 (Biii); the dotted line is the average with arrhythmogenic EHTs removed. In (Ci) to (Ciii), the corresponding EC_{50} values for isogenic lines were calculated using absolute force.

All error bars represent SEM. Significance was determined by Student's t test and one-way ANOVA, $^{*}p < 0.05$, $^{++}p < 0.01$, and $^{+++}p < 0.001$. n = 7 E99K1, 2 E99K1-Corr, 7 NC-Edit-E99K, 7 NC, 5 E99K2, and 6 E99K2-Corr. Red, mutant ACTC E99K; black, wild-type.

wild-type isogenic controls (Figure 3D). When contraction and relaxation velocities were analyzed, E99K1 line had significantly faster speeds than their corrected counterpart. In contrast, NC-Edit-E99K and E99K2 were similar or significantly slower (Figures 3B and 3C). Thus, there were differences between E99K1 hEHT responses relative to NC-Edit-E99K- or E99K2-derived hEHTs.

In previous studies the E99K-ACTC1 hypercontractile phenotype was shown to be due to an increase in myofibrillar Ca^{2+} sensitivity (Song et al., 2011, 2013). We therefore studied the effect of external Ca^{2+} concentration on EHT contractility (Figure 4). At 0 mM Ca^{2+} all EHTs were quiescent, whereas all were spontaneously beating at 0.5 mM Ca^{2+}. Increased $[Ca^{2+}]$ dependence was seen when comparing unedited E99K lines (E99K1 and E99K2) against the unedited wild-type NC line in both normalized contraction amplitude at different Ca^{2+} concentrations and in their EC_{50} values (Figures 4Ai and 4Aii). However, when comparing each line with its isogenic counterpart, increased contraction at lower Ca^{2+} concentrations was not seen in any of the three pairs, and neither were changes in EC_{50} values (Figures 4Bi–4iii and 4Ci–4iii). Thus, overall we could detect increased $[Ca^{2+}]$ dependence in the unedited mutant lines compared with wild-type, but this phenotype was not knocked in or corrected in the isogenic pairs.

E99K hiPSC-CMs Display Increased Arrhythmogenic Events

We next investigated whether E99K-ACTC1 mutants showed any difference in arrhythmogenic event frequency, since clinical and animal studies usually show ECG abnormalities and a varying degree of enhanced arrhythmia in mutation carriers (Arad et al., 2002; Olson et al., 2000; Monserrat et al., 2007; Song et al., 2011; Rowlands et al., 2017). Contraction traces obtained for EHTs clearly highlighted the presence of arrhythmogenic events (Figure 5A). To quantify these arrhythmogenic events in a non-biased manner, we used pClamp software as an unbiased method for analysis. First, the total twitch duration (combined contraction and relaxation time) was calculated for 211 E99K1, 218 NC, and 213 E99K2 wild-type contractions in seven EHTs. Twitch durations were averaged and found to be significantly different ($p < 0.001$) between the wild-type lines, at 0.51s for E99K1-Corr, 0.73s for NC, and 0.60 for E99K2 (Figure S3A).

Analysis then identified the number of abnormal contractions that failed to return to non-arrhythmogenic contraction time baseline during a normal event time (Figure 5B). For E99K1, 31% of contractions were found to be abnormal, although this was not significantly different when compared with wild-type isogenic controls (Figure 5Ci). NC-Edit-E99K and E99K2 had significantly more abnormal contractions when compared with their wild-type isogenic controls, with 54% and 46% abnormal contractions, respectively (Figures 5Cii and 5Ciii). No significant difference in the number of abnormal contractions was found between wild-type lines, with 18%, 17%, and 14% abnormal contractions for E99K1-Corr, NC, and E99K2-Corr, respectively.

We next evaluated whether Ca^{2+} concentration influenced arrhythmogenesis in EHTs fabricated from the six lines (Figure 5D). Relative to their wild-type isogenic counterparts, all E99K hEHTs showed more arrhythmogenic contractions, although significance was reached at differing Ca^{2+} levels. Thus, for E99K1 it was 3 mM Ca^{2+} for NC-Edit-E99K 0.5 mM and 1.5 mM Ca^{2+}, and for E99K2 3 mM and 5 mM Ca^{2+}.

To further support the 3D arrhythmogenic analysis, we implemented an alternative approach in a complementary 2D system. Nickase CRISPR/Cas9 was used to insert genetically encoded Ca^{2+} indicators (R-GECO1/GEM-GECO) into the safe locus *AAVS1* of the four parental and engineered hiPSC lines of E99K1 and NC. Correct insertion was confirmed by PCR and sequencing, while expression was validated by immunocytochemistry (Figure S4). Confocal line scans were taken during a 300-s time period to obtain traces and corresponding kymographs of spontaneous Ca^{2+} transients in hiPSC-CMs (Figures S4E and S4F). These line scans were used to calculate mean peak amplitudes and irregular delayed-after-depolarization Ca^{2+} transient events (events lower than 75% mean peak amplitude).

In 2D culture, arrhythmogenic events were shown to vary even at physiological Ca^{2+} concentration (1.8 mM), occurring at a frequency of 15.7% versus 6.7% for E99K1 versus E99K1-Corr and 17.4% versus 10.3% for NC-Edit-E99K versus NC (Figure 5E). Although there was no difference in R-GECO1 signal amplitude (F/F_0) between E99K1 lines, NC-Edit-E99K hiPSC-CMs displayed a ~50% lower GEM-GECO signal amplitude (F/F_0) relative to NC controls (Figure S4G), indicating a lower systolic Ca^{2+} peak. These data demonstrated both the E99K1 and NC backgrounds to exhibit increased arrhythmogenic events when the E99K-ACTC1 mutation is present.

E99K-Associated Abnormal Ca^{2+} Handling Is Absent when Introduced into Healthy Genetic Background

Due to its key regulatory role in excitation-contraction coupling, abnormal Ca^{2+} handling can contribute to severe disease phenotypes. HCM patients can exhibit prolonged Ca^{2+} transients (Lan et al., 2013), often attributed to the presence of sarcomeric protein mutations, such as E99K-ACTC1. Indeed, previous models have shown the E99K-ACTC1 mutation to increase Ca^{2+} sparks and Ca^{2+} transients in young transgenic mice (Rowlands et al., 2017).

To investigate whether abnormal Ca^{2+} handling could be detected, we used the CellOPTIQ cardiomyocyte analysis platform to assess intracellular Ca^{2+} transients in 2D cultures. Monolayers of hiPSC-CMs were loaded with Fluo4-AM, and Ca^{2+} transients were recorded using CellOPTIQ technology (Figure 6A). This 2D system showed that time to peak (TTP) was significantly altered ($p < 0.001$) in the E99K1 isogenic pair, with E99K1 exhibiting ~115% longer TTP than E99K1-Corr. NC-Edit-E99K and NC did not differ (Figure 6B). Ca^{2+} transient decline was measured as time from peak to 90% return to baseline (CTD_{90}). Analysis showed differences only in the E99K1 isogenic pair, with E99K1 exhibiting a 31.4% change relative to E99K1-Corr (Figure 6C).

Our earlier observations showed that contraction forces and arrhythmogenic events varied with differing extracellular Ca^{2+} concentrations (Figures 4A, 4B, and 5D). Therefore, Ca^{2+} transient analysis was carried at a range of extracellular Ca^{2+} concentrations to determine whether the abnormal transient decline was also dependent on the ability to handle extracellular Ca^{2+}. Although all lines exhibited prolonged CTD_{90} at lower extracellular Ca^{2+} concentration (Figure 6D), there was no difference in response to these changes between the NC isogenic lines. The higher CTD_{90} increase in E99K1 compared with E99K1-Corr was significant at lower than physiological Ca^{2+} concentrations (0.5–1 mM).

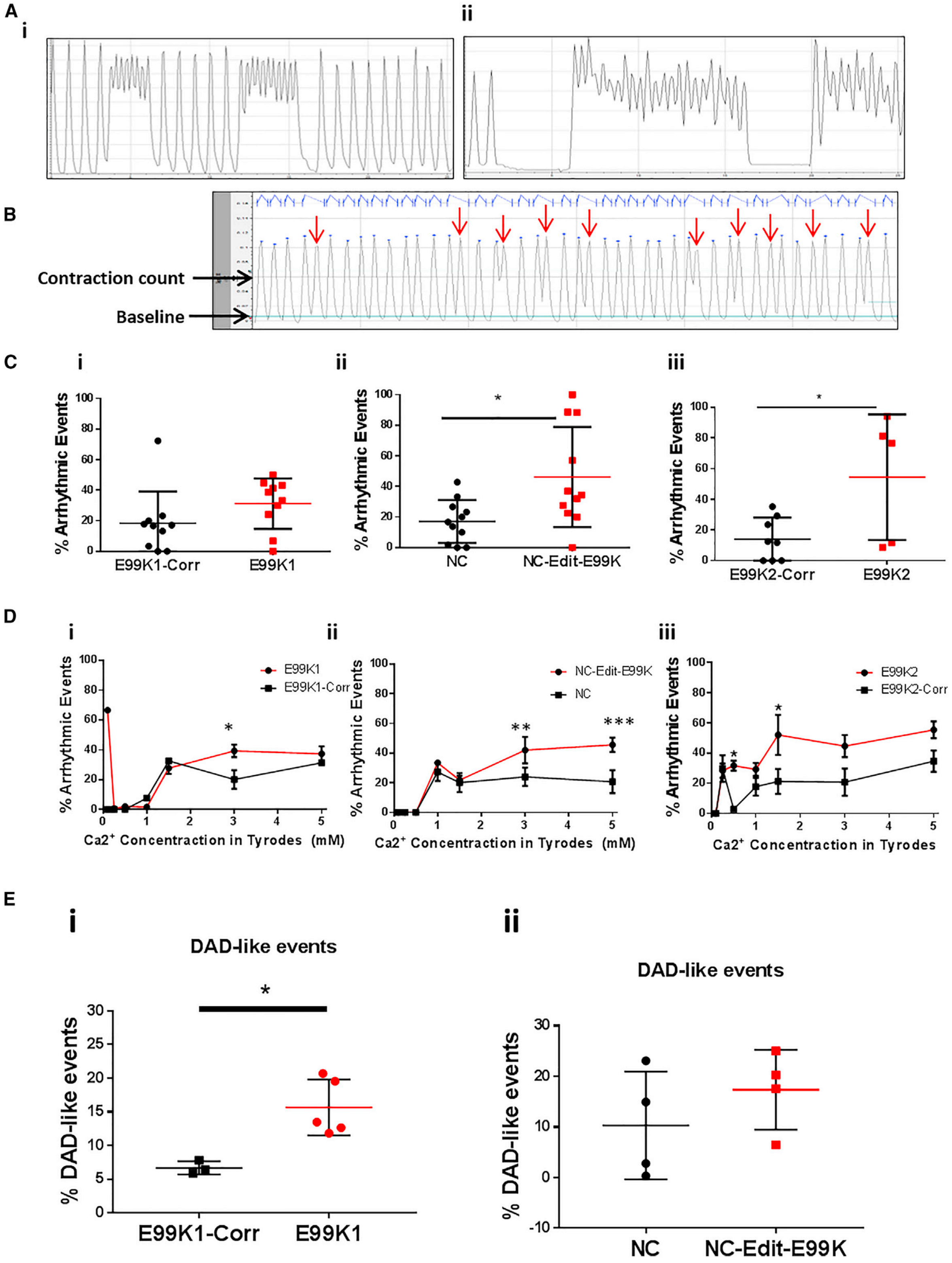

Figure 5. Arrhythmic Event Frequency in 3D hiPSC-CM EHTs and in 2D GECI Targeted hiPSC-CMs

In (Ai) and (Aii), example traces of arrhythmic contractions are shown. In (B), analysis of an example trace in pClamp is shown with arrows pointing to abnormal contractions identified through using a baseline and contraction count line. In (C), EHTs were stimulated at 1 Hz,

(legend continued on next page)

We investigated the effect of a 3D environment architecture on mutant Ca^{2+} transients. EHTs were loaded with the Ca^{2+} indicator, Fluo4-AM, and stimulated at 1 Hz in Tyrode's solution at 37°C. Ca^{2+} transient parameters were obtained through analysis of Ca^{2+}-dependent changes in fluorescence intensity within tissues. In 3D tissues there was no significant difference in TTP caused by the presence of the E99K-ACTC1 mutation (Figure 6E), but CTD_{90} data showed the same trend as the 2D model. In 3D EHTs, E99K1 CTD90 was 81.5% longer compared with E99K1-Corr, while NC-Edit-E99K and E99K2 showed no difference (Figure 6F). Collectively, both 2D and 3D data showed that the E99K-ACTC1 mutation caused abnormal Ca^{2+} handling in the genetic background of E99K1 but not in NC or E99K2.

Targeting Ca^{2+}Handling Pathways to Reduce Hypertrophic Signaling

To investigate whether the difference in phenotype severity was related to known hypertrophic signaling, we took forward isogenic pairs for E99K1 and NC. We investigated the expression of BNP because it has been reported to be >100-fold elevated in the plasma of HCM patients (Gardner, 2003). High-content imaging was used to classify cells in high, low, or negative BNP-expressing population, using predetermined empirical thresholds previously described for assessing BNP in hiPSC-CM lines (Carlson et al., 2013).

Analysis of BNP/cTnT/DAPI immunostained hiPSC-CMs (Figures S5A and S5B) showed no difference in BNP expression between NC-Edit-E99K and NC. In contrast, highly significant ($p < 0.001$) differences were seen in all three BNP populations for E99K1 versus E99K1-Corr, wherein E99K1 was 6.92- and 2.97-fold higher for BNP-high and -low cells, respectively, but 6.12-fold lower for BNP-negative cells. Thus, enhanced BNP expression in E99K-ACTC1 was only seen in the E99K1, but not NC genetic background, consistent with the difference in phenotype severities found for Ca^{2+} transients and contractility.

We hypothesized that the enhanced BNP expression seen in E99K1 could also be influenced by extracellular Ca^{2+}, as a mechanism behind its abnormal phenotypes. Although we observed no change in BNP expression at extracellular Ca^{2+} spanning physiological levels (1.8 mM), a reduction of BNP expression was observed in 0.1 mM and 0.5 mM Ca^{2+} (Figures S5C and S5D), indicating a role of Ca^{2+} in hypertrophic signaling pathways.

To investigate whether abnormal regulation of Ca^{2+} signaling pathways may be contributing to the mechanism of disease phenotypes in E99K-ACTC1 mutants, we next determined the expression levels of key Ca^{2+} signaling genes through a targeted qRT-PCR screen (Figure 7A). Analysis showed no differences between NC-Edit-E99K and NC. In contrast, E99K1 showed significantly ($p < 0.05$) lower expression of calmodulin (*CALM1*) and calmodulin-dependent protein kinase II delta (*CAMK2D*) than E99K1-Corr.

Although no difference was seen in expression of the Ca^{2+}-binding regulatory subunit of calcineurin (*PPP3CB*), expression of the calmodulin-binding catalytic subunit of calcineurin (*PPP3CA*) was significantly ($p < 0.01$) lower in E99K1 than in E99K1-Corr. The greatest difference was seen in the expression of the sarcoplasmic reticulum Ca^{2+}-binding calsequestrin 2 protein (*CASQ2*), with E99K1 showing 5.25-fold lower levels than E99K1-Corr. Expression of genes encoding the voltage-dependent Ca^{2+} channel (*CACNA1C*), the Ca^{2+} release inhibitor, phospholamban (*PLN*), and the Ca^{2+}-sensitive cysteine protease, calpain 1 (*CAPN1*), did not differ between E99K1 and E99K1-Corr (Figure 7). This expression data associated well with the differences seen in disease phenotype between the donors and suggests a key role for Ca^{2+} signaling pathways in disease penetrance.

Altogether, these data suggested that targeting cytosolic Ca^{2+} levels, and specifically targeting sarcoplasmic reticulum Ca^{2+}, release may reduce hypertrophic signaling. As such, ranolazine and dantrolene were identified as candidates with potential therapeutic benefit. Treatment with 10 μM of either drug significantly reduced BNP expression ($p < 0.01$) in all four lines, with the exception of dantrolene on NC-Edit-E99K (Figure 7B). Interestingly, in almost all cases, combined treatment with both drugs had a significantly greater effect ($p < 0.05$) than a single-drug regimen.

DISCUSSION

HCM is the most common inherited cardiomyopathy and has been investigated using *in vitro* systems and transgenic small animals. However, the relevance of these studies to

and arrhythmic events were counted and expressed as a percentage for E99K1 (Ai, n = 10, 10), NC (Ci, n = 11, 11), and E99K2 (Bi, n = 8, 5). In (D), arrhythmic events were counted in EHTs and expressed as a percentage during exposure to increasing Ca^{2+} concentrations, for E99K1 (Di, n = E99K1 7, E99K1-Corr 2), NC (Dii, n = 7 NC-Edit-E99K, 7 NC), and E99K2 (Diii, n = E99K2 5, E99K2-Corr 7). For 2D analysis hiPSC-CMs utilized genetically encoded expression of R-GECO1.0 or ratiometric GEM-GECO from the *AAVS1* locus. In (E), analysis of irregular Ca^{2+} transient events occurring during a 300-s line scan, with events lower than 75% mean peak amplitude, is shown for E99K1 and NC isogenic lines. E99K1-Corr n = 4, E99K1 n = 5, NC n = 4, NC-Edit-E99K n = 4.
All error bars represent SEM. Significance was determined by Student's t test, *$p < 0.05$, **$p < 0.01$, and ***$p < 0.001$. Red, mutant ACTC E99K; black, wild-type. See also Figure S3B.

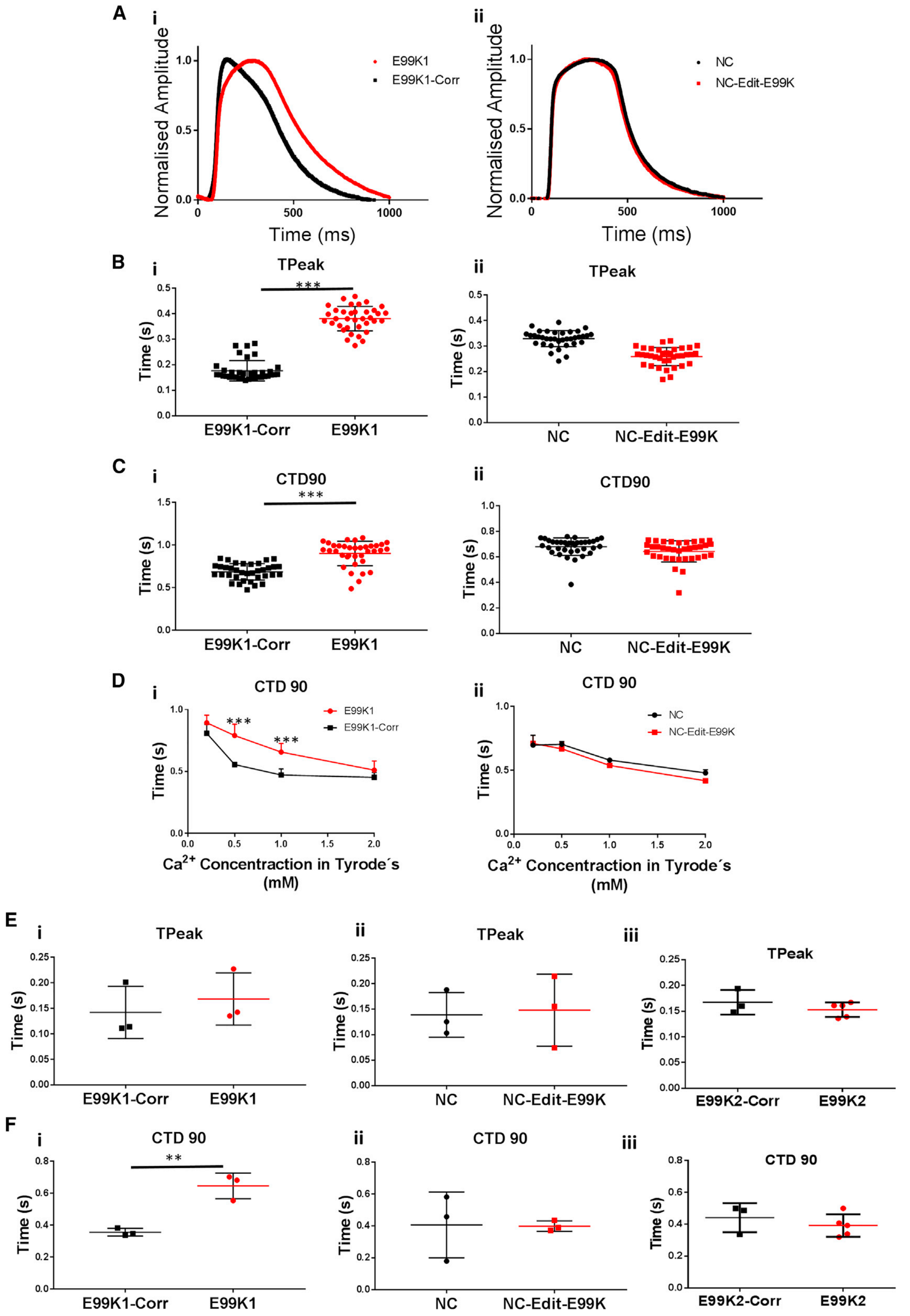

(legend on next page)

HCM in the human heart is not known. Study of HCM in human heart has been limited due to the difficulties in obtaining fresh tissue. Therefore, HCM mutations in the context of patient-derived hiPSC-CMs are important for understanding the disease phenotype.

We investigated hiPSC derived from three members of an extended family with the E99K-ACTC1 mutation (father and son with the mutation and brother non-carrier). We used CRISPR/Cas9 to create isogenic pairs of all three in which the mutation had been corrected or introduced to develop a physiologically relevant humanized E99K-ACTC1 model. This gave us a toolset of six hiPSC-CM lines that have enabled us to distinguish direct mutant-dependent effects from secondary effects related to patient age or genetic background that may affect the phenotypic expression of HCM. The use of isogenics proved to be crucial because our original hypothesis proved to be incorrect; namely, that phenotypic aberration would be consistent between all E99K-expressing lines. While pathogenic phenotypes were often observed in E99K1 hiPSC-CMs (father), only some, and most notably arrhythmias, were evident in the E99K2 and NC-Edit-E99K lines created from his sons.

The Cell Lines

Heterozygous hiPSC-CM from E99K1 and E99K2 showed expression of the E99K mutation, detected by mutation-specific antibody as did the CRISPR/Cas9-converted hiPSC-CM from NC. Interestingly, the expression of the mutation was segregated with 50% of cells expressing E99K and 50% of cells not expressing it. This indicates a stochastic monoallelic expression of *ACTC1* (Eckersley-Maslin and Spector, 2014). A similar monoalleleic expression of myosin heavy chain mRNA was suggested for the MYH7 R273G mutation (Kraft et al., 2016).

The significance of this is unknown but may be worth further investigation. In particular it has been speculated that a mosaic of wild-type and mutant cells having different contractile properties could disrupt cell-cell mechanical interactions and be the trigger for myocyte disarray and the subsequent interstitial fibrosis (Kraft et al., 2016). Interestingly, it is already known that in cardiac muscle the skeletal and cardiac actin isoforms are expressed is separate cells (Suurmeijer et al., 2003). The percentage of mutation expressed is proposed to be comparable with the pathological distribution of mutant to wild-type protein within the hearts of individuals affected with heterogeneous autosomal dominant cardiac disease: The 50% overall expression of the mutant protein compares with measurements of 39% (left ventricular free wall) and 19% (atrial-ventricular septum) in biopsies of two E99K patients (Song et al., 2011).

Contractility

In the transgenic mouse model, the E99K mutation increased myofilament Ca^{2+}-sensitivity of isometric force about 2-fold (Song et al., 2011). When EHT are activated maximally at 1.8 mM Ca^{2+} the maximum contraction, which in this case is auxotonic rather than isometric, ranged from 0.1 to 0.2 mN. There did not appear to be any detectable difference between native and CRISPR/Cas9 edited wild-type or E99K lines isogenic pairs. However, a large shift in $[Ca^{2+}]$ dependence was detected in unedited lines, with E99K EHTs having stronger contractions at lower levels of external Ca^{2+}. The millimolar concentration of Ca^{2+} in the medium is indirectly related to the micromolar $[Ca^{2+}]$ in the sarcoplasm that activates contraction. It was observed that the threshold $[Ca^{2+}]$ to activate contraction was lower for the unedited E99K mutant samples. Similarly, the EC_{50} of E99K EHTs was lower than that of the wild-type. This is compatible with the well-documented enhanced myofibrillar Ca^{2+} sensitivity of the E99K mutation *in vitro* and in the mouse model.

NC-Edit-E99K and E99K2 both had longer contraction times and decreased velocity of contraction compared with their isogenic controls. Both these isogenic pairs had the same phenotype even when one had the mutation knocked in and one had the mutation corrected. E99K1 has a high contraction rate. Analysis of sarcomere assembly showed a trend, albeit non-significant, toward increased disarray in E99K-expressing hiPSC-CMs (Figure S7). However, there was no apparent correlation between this parameter and the contraction measurements recorded.

For comparison with E99K transgenic mice, we see about 2-fold slower relaxation times but similar contraction times in isolated myofibrils, papillary muscle, and

Figure 6. Ca^{2+} Handling Properties in 2D hiPSC-CMs and 3D EHTs

hiPSC-CMs were loaded with Fluo4-AM and Ca^{2+} transients were recorded using CellOPTIQ technology (Ai and Aii). Time to peak (TPeak) was determined in each of the four lines (Bi and Bii). Technical n = 36. Ca^{2+} transient decline was measured in each of the DI and DII lines as a percentage of peak at 90% (Ci and Cii). Technical n = 36. Analysis was carried out on contractions during exposure to varied Ca^{2+} concentrations in Tyrode's solution (Di and Dii). n = 6. For 3D hiPSC-CM analysis, EHT were loaded with Fluo4-AM, stimulated at 1 Hz in Tyrode's solution at 37°C, and Ca^{2+} transients were recorded. EHT time to peak was determined in each of the six lines (Ei–Eiii). EHT Ca^{2+} transient decline was measured in each of the six lines as a percentage of peak at 90% (Fi–Fiii). n = 3 for all apart from E99K2 where n = 5.
All error bars represent SEM. Significance was determined by Student's t test, **p < 0.01 and ***p < 0.001. Red, mutant ACTC E99K; black, wild-type.

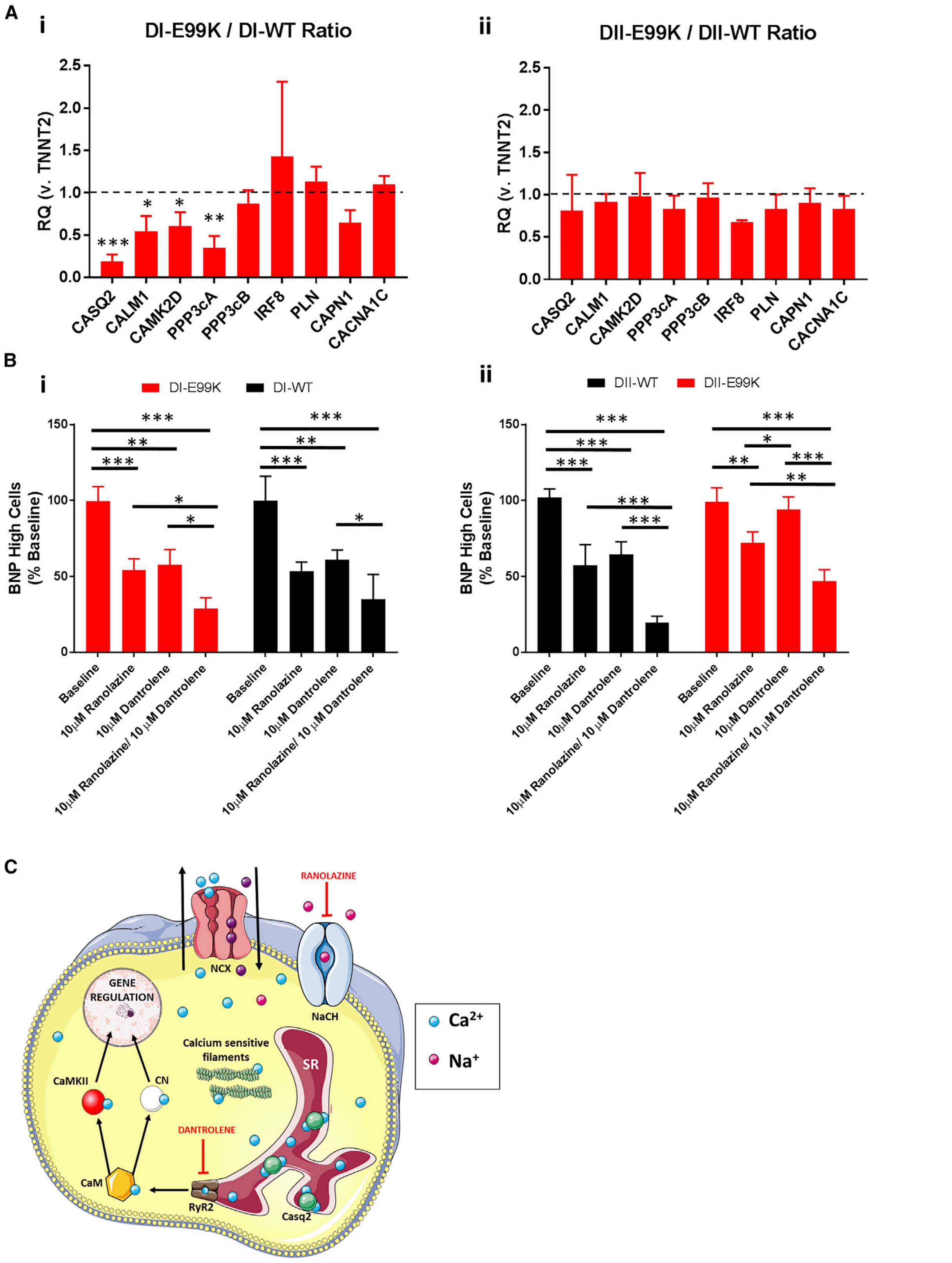

(legend on next page)

cardiomyocytes, compatible with a longer overall twitch time (Song et al., 2013; Rowlands et al., 2017). Moreover, recombinant human E99K actin also shows decreased association, dissociation, and slower velocities of interaction with myosin than wild-type (Bookwalter and Trybus, 2006).

Arrhythmia and Ca^{2+} Handling

ECG abnormalities are a hallmark of HCM and are commonly observed in the E99K patient cohort (Monserrat et al., 2007). The increased myofilament Ca^{2+} sensitivity caused by HCM mutations alters intracellular Ca^{2+}-buffering(Robinson et al., 2018), and this has been shown to be sufficient to provoke arrhythmia and ECG abnormalities in mice (Huke and Knollmann, 2010). An enhanced probability of arrhythmia has also been observed with the E99K mutation in transgenic mice (Song et al., 2011). Moreover, an increase in Ca^{2+} sparks and Ca^{2+} transients was observed that showed variable penetrance depending on background mouse strain (Rowlands et al., 2017). This trait was apparent in our hiPSC-CM EHTs: contractile arrhythmia increased at higher [Ca^{2+}] in both wild-type and E99K, but E99K EHTs showed a significantly greater frequency of arrhythmogenic events. This was further confirmed by studies using GECI Ca^{2+} sensors in 2D culture.

The difference in phenotype severity between the donors (E99K1 and NC) associates with expression of the natriuretic peptide, BNP, a known marker of hypertrophy. Expression was shown to be modulated by extracellular Ca^{2+} levels, indicating a regulatory role of Ca^{2+} in hypertrophic response. Subsequent analysis identified abnormal expression of calmodulin signaling pathway components in the severe, but not the mild, phenotype. This pathway is considered to be the primary sensor of changes in cellular free Ca^{2+} levels. As such, reduced expression of calmodulin, and the Ca^{2+}sensitive enzymes it activates (CaMKII and calcineurin), is likely to reduce the regulation of Ca^{2+} handling that is crucial for normal cardiac function. This pathway has previously been implicated in hypertrophy through its ability to regulate natriuretic peptide expression (Dewenter et al., 2017), supporting the hypothesis that abnormal Ca^{2+} regulation in E99K1 is the mechanism behind the greater severity of hypertrophic phenotype.

Interestingly, the greatest expression difference between the E99K1 isogenic lines was in *CASQ2* expression. Calsequestrin 2 is the main Ca^{2+} buffering protein of the sarcoplasmic reticulum. Reduced levels of calsequestrin 2 have been shown to increase the probability of spontaneous Ca^{2+} release for any given luminal free Ca^{2+} concentration (Faggioni and Knollmann, 2012). Coupled with reduced calmodulin-driven Ca^{2+} regulation, a cellular environment with increased Ca^{2+} release from the sarcoplasmic reticulum would further exacerbate the hypertrophic phenotype seen in E99K1.

The Origin of Phenotypic Variability

The common features of the E99K hiPSC-CM are compatible with the known role of the mutation in increasing myofilament Ca^{2+}-sensitivity and disrupting Ca^{2+} handling. However, there was considerable variability between the three patient samples, with E99K1 showing the greatest array of severe phenotypes. This may reflect pathophysiology and/or age, particularly given that HCM typically becomes symptomatic in the 2nd and 3rd decades of life. E99K1 was 48 years old with overt symptoms of HCM and LVNC, and conduction deficit, and thus is likely to have developed secondary abnormalities (Figure S1). In contrast, E99K2 was 19 years old and did not show abnormal ECG abnormalities. NC was 14 years old and, in this case, the mutation was introduced after the sample was taken, so one would expect there to be no secondary abnormalities.

Mutation load in mitochondrial DNA is higher in hiPSCs produced from older individuals (Kang et al., 2016). An age-dependent increase in mitochondrial DNA mutations, leading to increased reactive oxygen species production and activation of hypertrophic signals, has been proposed as the mechanism behind an age-dependent development of a cardiac hypertrophic phenotype (Marian, 2001). Mouse models have previously shown age to influence E99K-ACTC1-associated phenotypes, with older E99K-ACTC1 mice developing increased end-diastolic and end-systolic volumes (Song et al., 2011).

Thus, speculatively our model recapitulates a varied phenotype severity through an age-dependent, rather than a genetic background, based mechanism.

Limitations of the hiPSC System for This Study

We minimized technical variation by choosing directly related family members, from whom biopsies were harvested, prepared, and reprogrammed to hiPSC at the

Figure 7. Ca^{2+} Signaling in Hypertrophic hiPSC-CMs
(A) qPCR analysis of Ca^{2+} signaling gene expression ratios in E99K lines normalized to wild-type (WT) controls.
(B) The effect of two intracellular Ca^{2+}-altering drugs (ranolazine and dantrolene) on the percentage of highly expressing BNP hiPSC-CMs.
(C) Intracellular Ca^{2+} signaling pathways and the mechanism ranolazine and dantrolene reduce cytoplasmic Ca^{2+} levels. n = 3. NCX, sodium-Ca^{2+} exchanger; SR, sarcoplasmic reticulum.
Significance was determined by Student's t test, *$p < 0.05$, **$p < 0.01$, and ***$p < 0.001$. Red, mutant ACTC E99K; black, wild-type.

same time using the same methods; this extended to differentiation method, stage of differentiation, and cardiomyocyte purity, with cultures typically containing >90% α-actinin-positive cells (e.g., Figures 1 and 2). Assays were run on the same day wherever possible. Two clones per isogenic sets were used, wherein off-target events were not detected and genetic stability was retained, at least at the resolution of G-banding karyogram of 30 metaphase spreads. Nevertheless, any cells undergo stochastic epigenetic/genetic change relative to time in culture (Allegrucci et al., 2007; Smith and Whitney, 1980), with an additional issue for hiPSC being the retention of residual epigenetic memory that is refractory to reprogramming (Peng et al., 2011; Rowlands et al., 2017).

Differentiation was highly efficient, but the effect of the residual 1%–10% non-cardiomyocytes, which include mesodermal derivatives such as fibroblasts and smooth muscle cells (Sharma et al., 2015), can influence cell function. Deliberate mixing of hiPSC-CMs with non-cardiomyocyte populations (e.g., endothelial cells and fibroblasts) is known to alter force generation and drug responses (Ravenscroft et al., 2016). Purity of hPSC-CMs can be improved by techniques such as metabolic or genetic selection (Anderson et al., 2007; Sharma et al., 2015). However, these approaches place additional stress on the cardiomyocytes, are cumbersome, and/or require additional prolonged culture periods to genetically engineer individual hiPSC lines, which risks introducing further epigenetic/genetic change.

The ideal way to overcome these limitations is to increase the number of hPSC lines within the study and ensure each is matched with a Cas9/CRISPR isogenic control. However, there are currently no reports of high-volume hiPSC production, with multiple clones and isogenic pairing, likely due to the labor required for such an initiative.

Finally, it is well established that hiPSC-CMs immature relative to adult cardiomyocytes (see, e.g., Denning et al., 2016 for review). This includes the machinery that underpins HCM, including Ca^{2+} handling, t-tubules, sarcomeric alignment, and mitochondrial content. Despite these deficiencies, hiPSC-CMs are becoming established as a modality to evaluate successfully the impact of disease and drugs on cardiomyocyte function (Mosqueira et al., 2018; Gintant et al., 2017). The concerted international effort to improve hiPSC-CM maturity via approaches such as metabolic switching (Correia et al., 2017) and electrical pacing (Sun and Nunes, 2016) will further advance the utility of these models.

The Use of E99K hiPSC-CM in Drug Screening

Figure 7C shows an overview of cytosolic Ca^{2+} released from the sarcoplasmic reticulum, activating calmodulin-dependent signaling pathways, that ultimately alters gene expression. Blocking sarcoplasmic reticulum Ca^{2+} release through inhibiting the ryanodine receptor-2 with dantrolene, and promoting Ca^{2+} efflux through enhancing the sodium-Ca^{2+} exchanger with ranolazine, lowers cytosolic Ca^{2+} and reduces hypertrophic signaling. We demonstrated that targeting of intracellular Ca^{2+} levels, by promoting Ca^{2+} efflux (ranolazine) and limiting sarcoplasmic reticulum Ca^{2+} release (dantrolene), reduced hypertrophic signaling.

Ranolazine was identified as a therapeutic candidate as an enhancer of the sodium-Ca^{2+} exchanger (outward mode) by blocking late sodium current and thereby indirectly promoting Ca^{2+} efflux. Contrastingly, the mode of action for dantrolene directly limits Ca^{2+} release from the sarcoplasmic reticulum during systole through its activity as a ryanodine receptor antagonist. These drugs have previously been shown to have a potential benefit in other HCM models, with ranolazine used to ameliorate diastolic function in HCM patient-derived cardiomyocytes (Coppini et al., 2012), and dantrolene applied as an antiarrhythmic agent in mouse models of HCM (Jung et al., 2012; Okuda et al., 2018). Here we show these drugs to have potential benefit in cases of E99K-ACTC1-associated HCM, and our results indicate that a dual treatment targeting both mechanisms may have enhanced therapeutic benefit. Dual treatments such as this have not been reported clinically and, as such, hiPSC-CMs provide an opportunity to trial new combinations with potential patient benefit. Moreover, the model we present now provides opportunities for high-throughput and/or high-content evaluation of the ability of drugs, singly or in combination, to rescue the phenotypes identified, particularly using hiPSC-CMs derived from the heterozygote father E99K1.

EXPERIMENTAL PROCEDURES

hiPSC Generation

Fibroblast cultures were expanded in fibroblast growth medium and transduced using CytoTune2.0-iPS Sendai Reprogramming (Thermo Fisher #A16517). Following transduction, cultures were changed to Essential 6 medium (Life Technologies #A1516401) supplemented with 100 ng/mL basic fibroblast growth factor (Peprotech #100-18B) and medium exchanged daily until colonies appeared (5–10 days), after which the medium was changed to Essential 8 medium (Life Technologies #A1517001). hiPSC colonies were expanded and isolated by manual dissection. Two clones were used per line and used in subsequent studies.

CRISPR/Cas9 Genome Editing of hiPSC

The strategy for correcting/introducing the E99K-ACTC1 mutation to generate isogenic control lines has been previously described in detail (Kondrashov et al., 2018). In brief, an *ACTC1* targeting vector was constructed containing a dual drug selection cassette (Puro-ΔTK) flanked by *PiggyBac* recombination sites, and the two *ACTC1*

sequences (1 kb upstream and 1 kb downstream) homologous to the endogenous target locus cut site. One microgram of *ACTC1* targeting vector was transfected into 1 × 10^6 hiPSCs, with 1 μg of hCas9 plasmid and 1 μg of guide RNA pU6 vector using an Amaxa 4D system (Lonza). Twenty-four hours after transfection, the medium was supplemented with 0.25 μg/mL puromycin (Life Technologies #A1113802) for positive selection of clones for up to 2 weeks. The puromycin-positive clones were then harvested using TrypLE (Life Technologies #12563029), cultured for 24 hr, and transfected with 3 μg of transposase plasmid transfection using Fugene HD transfection reagent (Promega #E2311). On the next day, cells were exposed to medium containing 2 μg/mL ganciclovir (Sigma #G2536) for negative selection of *PiggyBac* excision clones for up to 2 weeks.

Monolayer Cardiac Differentiation of hiPSCs

hiPSC differentiation was performed by seeding vessels at approximately 20,000–40,000 cells/cm^2, as described above. Fresh E8 was added the next day and the pre-conditioning step of hiPSC was performed the day after, by adding a Matrigel overlay (Matrigel diluted 1:100 in StemPro34 Serum Free Medium [SP34, Gibco #10639011]), supplemented with 1 ng/mL BMP4 (R&D Systems #314-BP-050). Approximately 16 hr later, medium was replaced by SP34 supplemented with 8 ng/mL activin A (ActA, Life Technologies #PHC9564) and 10 ng/mL bone morphogenetic protein 4 (BMP4). Forty-eight hours later, medium was changed to RPMI supplemented with B27 without insulin (−INS, Life Technologies #A1895601) and KY0211 (R&D #4731) and XAV939 (R&D #3748), both at 100 μM. These small molecules were added again 2 days later, in RPMI supplemented with B27 with insulin (+INS, Life Technologies #0080085-SA) instead. Thereafter, medium was changed every 2–3 days by fresh RPMI + B27 + INS until day 15 of differentiation, when spontaneously beating hiPSC-CM were dissociated and replated as below, and kept in RPMI + B27 + INS for approximately 10 days until phenotypic assays were performed.

hEHT Fabrication and Maintenance

hEHTs were fabricated as previously described (Breckwoldt et al., 2017; Schaaf et al., 2014). In brief, Teflon spacers (EHT Technologies #C0002) were inserted in 2% ultra-pure agarose (Invitrogen #15510-027) solution pipetted into 24-well plates (Nunc) before gelification. Thereafter, spacers were removed and silicone racks (EHT Technologies #C0001) were placed in the aperture left by the agarose casting molds. Subsequently, freshly dissociated hiPSC-CMs were resuspended in DMEM (Biochrom, F0415), supplemented with 10% heat-inactivated fetal calf serum (Biochrom #S0615), 1% penicillin/streptomycin (PEST, Gibco), 2 mM L-glutamine, 2× DMEM (equalizing the hypotonic volume of fibrinogen plus thrombin), 10% Matrigel, 0.1% Y-27632, and 5 mg/mL fibrinogen (Sigma #F8630). The CM fibrinogen mix was then quickly mixed with 3 U of thrombin (Sigma #T7513) and pipetted into the aperture between the silicone posts. Forming hEHTs were then incubated for 2 hr at 37°C and 7% CO_2 and subsequently moved to new 24-well plates filled with DMEM supplemented with 10% horse serum, 10 μg/mL insulin (Sigma #I9278), 33 μg/mL aprotinin (Sigma #A1153), and 1% (v/v) PEST, termed EHT medium. Each hEHT consisted of 1 million cells and was fed every other day for 2–3 weeks.

Analysis of hEHTs

Contractile force was analyzed as previously described (Mannhardt et al., 2016). In brief, 2- to 3-week-old hEHTs were immersed in modified Tyrode's solution (120 mM NaCl, 5.4 mM KCl, 1 mM $MgCl_2$, 0.4 mM NaH_2PO_4, 22.6 mM $NaHCO_3$, 5 mM glucose, 0.05 mM Na_2EDTA, and 25 mM HEPES) and varied Ca^{2+} concentration (0–5 mM $CaCl_2$), and the 24-well plate was placed inside a transparent chamber to maintain homeostatic temperature (37°C), CO_2 (5%), and O_2 (40%). Automated video-optical recordings of silicone post deflection were enabled by the EHT analysis instrument (EHT Technologies #A0001) whereby a video camera placed above the chamber tracked hEHT movement and a separate computer running a customized software (CMTV GmbH) determined contractile force based on the known mechanical properties of the silicone posts. When indicated, EHTs were electrically paced (2 V, 1–2 Hz, impulse duration 4 ms) with carbon electrodes using a Grass S88X stimulator (Astro-Med). The contraction peaks were analyzed in terms of force, and contraction (T1) to peak from 20% peak height and relaxation time (T2) from peak to 80% of peak height. To quantify arrhythmogenic events without bias, we used pClamp software to identify the number of abnormal contractions compared with normal baseline events.

Statistics

Statistical analysis was performed using GraphPad Prism (v7, GraphPad, La Jolla, CA, USA) software, evaluated by Student's t tests or unpaired one-way ANOVA, to compare isogenic pairs or between genotypes. Differences were considered significant at $^{*}p < 0.05$, $^{**}p < 0.01$, and $^{***}p < 0.001$. Numbers denoted "n" indicate independent experiments unless stated otherwise.

SUPPLEMENTAL INFORMATION

Supplemental Information includes Supplemental Experimental Procedures and seven figures and can be found with this article online at https://doi.org/10.1016/j.stemcr.2018.10.006.

AUTHOR CONTRIBUTIONS

J.G.W.S. and T.O. contributed equally to this work. J.G.W.S and T.O. took lead on performing experimental work, with J.G.W.S leading 2D experiments and T.O. leading 3D experiments. J.R.B., D.M., E.S., I.M., A.P., R.B.-V., and L.M. performed some experimental work. J.G.W.S. and T.O. wrote the manuscript with support from C.D., S.M., T.E., A.H., R.B.-V., L.M., and S.E.H. The project was conceived and supervised by C.D., S.M., T.E., A.H., and S.E.H.

ACKNOWLEDGMENTS

This work was supported 609 by the British Heart Foundation (grant numbers SP/15/9/31605, RG/15/6/31436, PG/14/59/31000, RG/14/1/30588, RM/13/30157, P47352/CRM); the National Centre for the Replacement, Refinement & Reduction of Animals in Research (grant numbers CRACK-IT:35911-259146, NC/K000225/1); and the Medical Research Council (grant number MR/M017354/1) through the Development of Metrics and Quality

Standards for Scale up of Human Pluripotent Stem Cells; the Britain Israel Research and Academic Exchange Partnership (04BX14CDLG); Centro de Investigación Biomédica en Red (CIBERCV), "Instituto de Salud Carlos III" (grant number CB16/11/00425 617), FEDER "Unión Europea, Una forma de hacer Europa"; the German Research Foundation (DFG-Es-88/12-1, HA3423/5-1); European Research Council (ERC-AG-IndivuHeart); European Commission (FP7-Biodesign); German Centre for Cardiovascular Research (DZHK) and the German Ministry of Education and Research, the Freie und Hansestadt Hamburg.

Received: September 27, 2018
Revised: October 4, 2018
Accepted: October 5, 2018
Published: November 1, 2018

REFERENCES

Allegrucci, C., Wu, Y.Z., Thurston, A., Denning, C.N., Priddle, H., Mummery, C.L., Ward-van Oostwaard, D., Andrews, P.W., Stojkovic, M., Smith, N., et al. (2007). Restriction landmark genome scanning identifies culture-induced DNA methylation instability in the human embryonic stem cell epigenome. Hum. Mol. Genet. *16*, 1253–1268.

Anderson, D., Self, T., Mellor, I.R., Goh, G., Hill, S.J., and Denning, C. (2007). Transgenic enrichment of cardiomyocytes from human embryonic stem cells. Mol. Ther. *15*, 2027–2036.

Arad, M., Seidman, J.G., and Seidman, C.E. (2002). Phenotypic diversity in hypertrophic cardiomyopathy. Hum. Mol. Genet. *11*, 2499–2506.

Bai, F., Caster, H.M., Dawson, J.F., and Kawai, M. (2015). The immediate effect of HCM causing actin mutants E99K and A230V on actin-Tm-myosin interaction in thin-filament reconstituted myocardium. J. Mol. Cell. Cardiol. *79*, 123–132.

Bookwalter, C.S., and Trybus, K.M. (2006). Functional consequences of a mutation in an expressed human alpha-cardiac actin at a site implicated in familial hypertrophic cardiomyopathy. J. Biol. Chem. *281*, 16777–16784.

Breckwoldt, K., Letuffe-Breniere, D., Mannhardt, I., Schulze, T., Ulmer, B., Werner, T., Benzin, A., Klampe, B., Reinsch, M.C., Laufer, S., et al. (2017). Differentiation of cardiomyocytes and generation of human engineered heart tissue. Nat. Protoc. *12*, 1177–1197.

Carlson, C., Koonce, C., Aoyama, N., Einhorn, S., Fiene, S., Thompson, A., Swanson, B., Anson, B., and Kattman, S. (2013). Phenotypic screening with human iPS cell-derived cardiomyocytes: HTS-compatible assays for interrogating cardiac hypertrophy. J. Biomol. Screen *18*, 1203–1211.

Coppini, R., Ferrantini, C., Yao, L., Fan, P., Del Lungo, M., Stillitano, F., Sartiani, L., Tosi, B., Suffredini, S., Tesi, C., et al. (2012). Late sodium current inhibition reverses electro-mechanical dysfunction in human hypertrophic cardiomyopathy. Circulation *127*, 575–584.

Correia, C., Koshkin, A., Duarte, P., Hu, D., Teixeira, A., Domian, I., Serra, M., and Alves, P.M. (2017). Distinct carbon sources affect structural and functional maturation of cardiomyocytes derived from human pluripotent stem cells. Sci. Rep. *7*, 8590.

Debold, E.P., Saber, W., Cheema, Y., Bookwalter, C.S., Trybus, K.M., Warshaw, D.M., and Vanburen, P. (2010). Human actin mutations associated with hypertrophic and dilated cardiomyopathies demonstrate distinct thin filament regulatory properties in vitro. J. Mol. Cell. Cardiol. *48*, 286–292.

Denning, C., Borgdorff, V., Crutchley, J., Firth, K.S., George, V., Kalra, S., Kondrashov, A., Hoang, M.D., Mosqueira, D., Patel, A., et al. (2016). Cardiomyocytes from human pluripotent stem cells: from laboratory curiosity to industrial biomedical platform. Biochim. Biophys. Acta *1863*, 1728–1748.

Dewenter, M., von der Lieth, A., Katus, H.A., and Backs, J. (2017). Calcium signaling and transcriptional regulation in cardiomyocytes. Circ. Res. *121*, 1000–1020.

Eckersley-Maslin, M.A., and Spector, D.L. (2014). Random monoallelic expression: regulating gene expression one allele at a time. Trends Genet. *30*, 237–244.

Eschenhagen, T., Eder, A., Vollert, I., and Hansen, A. (2012). Physiological aspects of cardiac tissue engineering. Am. J. Physiol. Heart Circ. Physiol. *303*, H133–H143.

Faggioni, M., and Knollmann, B.C. (2012). Calsequestrin 2 and arrhythmias. Am. J. Physiol. Heart Circ. Physiol. *302*, H1250–H1260.

Gardner, D.G. (2003). Natriuretic peptides: markers or modulators of cardiac hypertrophy? Trends Endocrinol. Metab. *14*, 411–416.

Gintant, G., Fermini, B., Stockbridge, N., and Strauss, D. (2017). The evolving roles of human iPSC-derived cardiomyocytes in drug safety and discovery. Cell Stem Cell *21*, 14–17.

Huke, S., and Knollmann, B.C. (2010). 'Increased myofilament Ca^{2+}-sensitivity and arrhythmia susceptibility'. J. Mol. Cell. Cardiol. *48*, 824–833.

Jung, C.B., Moretti, A., Mederos y Schnitzler, M., Iop, L., Storch, U., Bellin, M., Dorn, T., Ruppenthal, S., Pfeiffer, S., Goedel, A., et al. (2012). Dantrolene rescues arrhythmogenic RYR2 defect in a patient-specific stem cell model of catecholaminergic polymorphic ventricular tachycardia. EMBO Mol. Med. *4*, 180–191.

Kang, E., Wang, X., Tippner-Hedges, R., Ma, H., Folmes, C.D., Gutierrez, N.M., Lee, Y., Van Dyken, C., Ahmed, R., Li, Y., et al. (2016). Age-related accumulation of somatic mitochondrial DNA mutations in adult-derived human iPSCs. Cell Stem Cell *18*, 625–636.

Kaski, J.P., Syrris, P., Esteban, M.T., Jenkins, S., Pantazis, A., Deanfield, J.E., McKenna, W.J., and Elliott, P.M. (2009). Prevalence of sarcomere protein gene mutations in preadolescent children with hypertrophic cardiomyopathy. Circ. Cardiovasc. Genet. *2*, 436–441.

Kondrashov, A., Duc Hoang, M., Smith, J.G.W., Bhagwan, J.R., Duncan, G., Mosqueira, D., Munoz, M.B., Vo, N.T.N., and Denning, C. (2018). Simplified footprint-free Cas9/CRISPR editing of cardiac-associated genes in human pluripotent stem cells. Stem Cells Dev. *27*, 391–404.

Kraft, T., Montag, J., Radocaj, A., and Brenner, B. (2016). Hypertrophic cardiomyopathy: cell-to-cell imbalance in gene expression and contraction force as trigger for disease phenotype development. Circ. Res. *119*, 992–995.

Lan, F., Lee, A.S., Liang, P., Sanchez-Freire, V., Nguyen, P.K., Wang, L., Han, L., Yen, M., Wang, Y., Sun, N., et al. (2013). Abnormal calcium handling properties underlie familial hypertrophic

cardiomyopathy pathology in patient-specific induced pluripotent stem cells. Cell Stem Cell *12*, 101–113.

Mannhardt, I., Breckwoldt, K., Letuffe-Breniere, D., Schaaf, S., Schulz, H., Neuber, C., Benzin, A., Werner, T., Eder, A., Schulze, T., et al. (2016). Human engineered heart tissue: analysis of contractile force. Stem Cell Rep. *7*, 29–42.

Marian, A.J. (2001). On genetic and phenotypic variability of hypertrophic cardiomyopathy: nature versus nurture. J. Am. Coll. Cardiol. *38*, 331–334.

Maron, B.J., Maron, B.J., Towbin, J.A., Thiene, G., Antzelevitch, C., Corrado, D., Arnett, D., Moss, A.J., Seidman, C.E., Young, J.B., et al. (2006). Contemporary definitions and classification of the cardiomyopathies: an American Heart Association scientific statement from the Council on Clinical Cardiology, Heart Failure and Transplantation Committee; quality of care and outcomes research and functional genomics and translational biology interdisciplinary working groups; and Council on Epidemiology and Prevention. Circulation *113*, 1807–1816.

Marston, S.B. (2011). How do mutations in contractile proteins cause the primary familial cardiomyopathies? J. Cardiovasc. Transl. Res. *4*, 245–255.

Mogensen, J., Klausen, I.C., Pedersen, A.K., Egeblad, H., Bross, P., Kruse, T.A., Gregersen, N., Hansen, P.S., Baandrup, U., and Borglum, A.D. (1999). Alpha-cardiac actin is a novel disease gene in familial hypertrophic cardiomyopathy. J. Clin. Invest. *103*, R39–R43.

Monserrat, L., Hermida-Prieto, M., Fernandez, X., Rodriguez, I., Dumont, C., Cazon, L., Cuesta, M.G., Gonzalez-Juanatey, C., Peteiro, J., Alvarez, N., et al. (2007). Mutation in the alpha-cardiac actin gene associated with apical hypertrophic cardiomyopathy, left ventricular non-compaction, and septal defects. Eur. Heart J. *28*, 1953–1961.

Morita, H., Rehm, H.L., Menesses, A., McDonough, B., Roberts, A.E., Kucherlapati, R., Towbin, J.A., Seidman, J.G., and Seidman, C.E. (2008). Shared genetic causes of cardiac hypertrophy in children and adults. N. Engl. J. Med. *358*, 1899–1908.

Mosqueira, D., Mannhardt, I., Bhagwan, J.R., Lis-Slimak, K., Katili, P., Scott, E., Hassan, M., Prondzynski, M., Harmer, S.C., Tinker, A., et al. (2018). CRISPR/Cas9 editing in human pluripotent stem cell-cardiomyocytes highlights arrhythmias, hypocontractility, and energy depletion as potential therapeutic targets for hypertrophic cardiomyopathy. Eur. Heart J. https://doi.org/10.1093/eurheartj/ehy249.

Okuda, S., Sufu-Shimizu, Y., Kato, T., Fukuda, M., Nishimura, S., Oda, T., Kobayashi, S., Yamamoto, T., Morimoto, S., and Yano, M. (2018). CaMKII-mediated phosphorylation of RyR2 plays a crucial role in aberrant Ca(2+) release as an arrhythmogenic substrate in cardiac troponin T-related familial hypertrophic cardiomyopathy. Biochem. Biophys. Res. Commun. *496*, 1250–1256.

Olivotto, I., Girolami, F., Ackerman, M.J., Nistri, S., Bos, J.M., Zachara, E., Ommen, S.R., Theis, J.L., Vaubel, R.A., Re, F., et al. (2008). Myofilament protein gene mutation screening and outcome of patients with hypertrophic cardiomyopathy. Mayo Clin. Proc. *83*, 630–638.

Olson, T.M., Michels, V.V., Thibodeau, S.N., Tai, Y.S., and Keating, M.T. (1998). Actin mutations in dilated cardiomyopathy, a heritable form of heart failure. Science *280*, 750–752.

Olson, T.M., Doan, T.P., Kishimoto, N.Y., Whitby, F.G., Ackerman, M.J., and Fananapazir, L. (2000). Inherited and de novo mutations in the cardiac actin gene cause hypertrophic cardiomyopathy. J. Mol. Cell. Cardiol. *32*, 1687–1694.

Peng, H., Yang, X.P., Carretero, O.A., Nakagawa, P., D'Ambrosio, M., Leung, P., Xu, J., Peterson, E.L., Gonzalez, G.E., Harding, P., and Rhaleb, N.E. (2011). Angiotensin II-induced dilated cardiomyopathy in Balb/c but not C57BL/6J mice. Exp. Physiol. *96*, 756–764.

Ravenscroft, S.M., Pointon, A., Williams, A.W., Cross, M.J., and Sidaway, J.E. (2016). Cardiac non-myocyte cells show enhanced pharmacological function suggestive of contractile maturity in stem cell derived cardiomyocyte microtissues. Toxicol. Sci. *152*, 99–112.

Robinson, P., Liu, X., Sparrow, A., Patel, S., Zhang, Y.H., Casadei, B., Watkins, H., and Redwood, C. (2018). Hypertrophic cardiomyopathy mutations increase myofilament Ca^{2+} buffering, alter intracellular Ca^{2+} handling and stimulate Ca^{2+} dependent signalling. J. Biol. Chem. *293*, 10487–10499.

Rowlands, C., Owen, T., Lawal, S., Cao, S., Pandey, S., Yang, H.Y., Song, W., Wilkinson, R., Alvarez-Laviada, A., Gehmlich, K., et al. (2017). Age and strain related aberrant Ca(2+) release is associated with sudden cardiac death in the ACTC E99K mouse model of hypertrophic cardiomyopathy. Am. J. Physiol. Heart Circ. Physiol. *313*, H1213–H1226.

Rubenstein, P.A., and Martin, D.J. (1983). NH2-terminal processing of *Drosophila melanogaster* actin. Sequential removal of two amino acids. J. Biol. Chem. *258*, 11354–11360.

Schaaf, S., Eder, A., Vollert, I., Stohr, A., Hansen, A., and Eschenhagen, T. (2014). Generation of strip-format fibrin-based engineered heart tissue (EHT). Methods Mol. Biol. *1181*, 121–129.

Sedaghat-Hamedani, F., Kayvanpour, E., Tugrul, O.F., Lai, A., Amr, A., Haas, J., Proctor, T., Ehlermann, P., Jensen, K., Katus, H.A., and Meder, B. (2018). Clinical outcomes associated with sarcomere mutations in hypertrophic cardiomyopathy: a meta-analysis on 7675 individuals. Clin. Res. Cardiol. *107*, 30–41.

Sharma, A., Li, G., Rajarajan, K., Hamaguchi, R., Burridge, P.W., and Wu, S.M. (2015). Derivation of highly purified cardiomyocytes from human induced pluripotent stem cells using small molecule-modulated differentiation and subsequent glucose starvation. J. Vis. Exp. https://doi.org/10.3791/52628.

Smith, J.R., and Whitney, R.G. (1980). Intraclonal variation in proliferative potential of human diploid fibroblasts: stochastic mechanism for cellular aging. Science *207*, 82–84.

Song, W., Dyer, E., Stuckey, D., Copeland, O., Leung, M., Bayliss, C., Messer, A.E., Wilkinson, R., Tremoleda, J., Schneider, M., et al. (2011). Molecular mechanism of the Glu99lys mutation in cardiac actin (ACTC gene) that causes apical hypertrophy in man and mouse. J. Biol. Chem. *286*, 27582–27593.

Song, W., Vikhorev, P.G., Kashyap, M.N., Rowlands, C., Ferenczi, M.A., Woledge, R.C., MacLeod, K., Marston, S., and

Curtin, N.A. (2013). Mechanical and energetic properties of papillary muscle from ACTC E99K transgenic mouse models of hypertrophic cardiomyopathy. Am. J. Physiol. Heart Circ. Physiol. *304*, H1513–H1524.

Spudich, J.A. (2014). Hypertrophic and dilated cardiomyopathy: four decades of basic research on muscle lead to potential therapeutic approaches to these devastating genetic diseases. Biophys. J. *106*, 1236–1249.

Sun, X., and Nunes, S.S. (2016). Biowire platform for maturation of human pluripotent stem cell-derived cardiomyocytes. Methods *101*, 21–26.

Suurmeijer, A.J., Clement, S., Francesconi, A., Bocchi, L., Angelini, A., Van Veldhuisen, D.J., Spagnoli, L.G., Gabbiani, G., and Orlandi, A. (2003). Alpha-actin isoform distribution in normal and failing human heart: a morphological, morphometric, and biochemical study. J. Pathol. *199*, 387–397.

Stem Cell Reports, Volume *11*

Supplemental Information

Isogenic Pairs of hiPSC-CMs with Hypertrophic Cardiomyopathy/ LVNC-Associated ACTC1 E99K Mutation Unveil Differential Functional Deficits

James G.W. Smith, Thomas Owen, Jamie R. Bhagwan, Diogo Mosqueira, Elizabeth Scott, Ingra Mannhardt, Asha Patel, Roberto Barriales-Villa, Lorenzo Monserrat, Arne Hansen, Thomas Eschenhagen, Sian E. Harding, Steve Marston, and Chris Denning

Supplementary Figures

a)

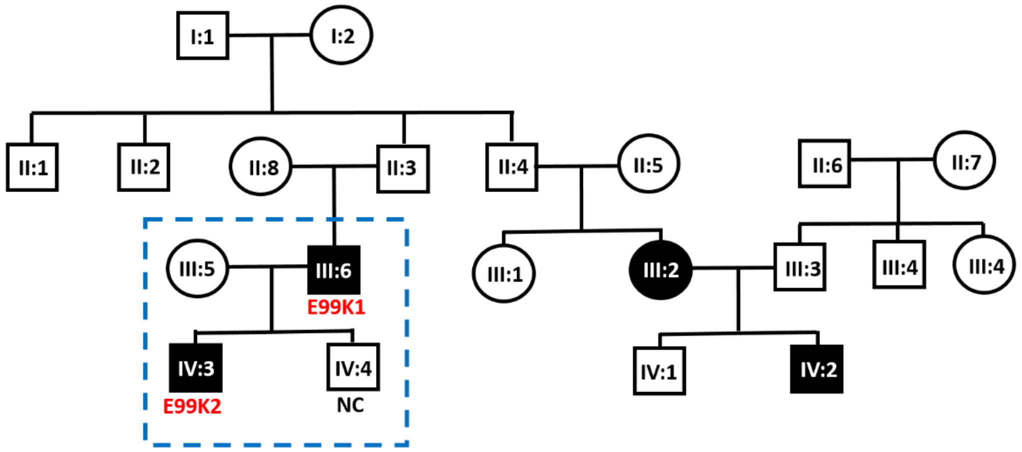

b)

Sample	Age of Diagnosis	Age of iPSC production	Maximum WT (mm)	Segment with maximum WT	Compact thickness (mm)	Compact/ non-compact	Left atrium diameter (mm)	LV diameters (end diastolic/end systolic) (mm)	Electrocardiogram	Treatment
E99K1	28	48	23	Apical	12	0.52	37	46	Abnormal Q	Bisoprolol
NC	-	14	-	-	-	-	-	-	-	-
E99K2	11	19	20	Apical	8	0.4	29	44	Normal	None

Figure S1. Details of donor samples. (a) Family tree showing the known E99K carriers and highlighting the three individuals from whom iPSC were prepared. **(b)** Clinical details of the three individuals from whom iPSC were prepared.

a)

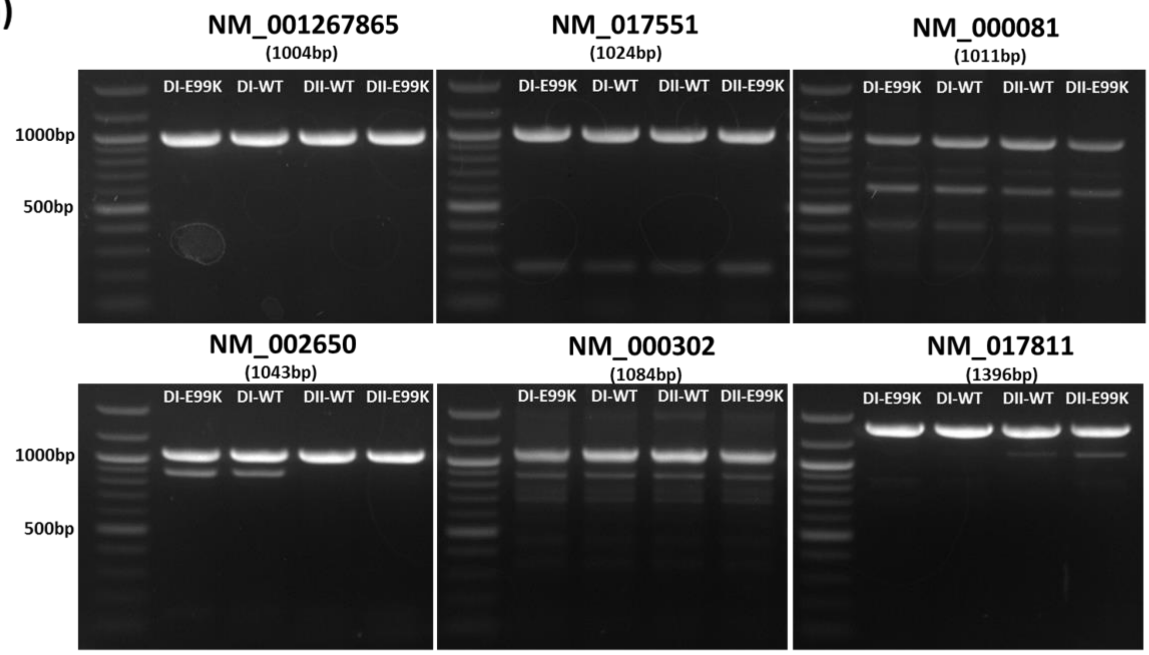

b)

Gene: Offtarget:		NM_001267865 GAGATAACACTG ATGCCCTGGAG (4MMs)	NM_017551 AGGTGAACAGTA GTCCCCTGGAG (4MMs)	NM_000081 CAATTAACAGTA GTTCCCTCTAG (4MMs)	NM_002650 GAGTTCTCAGGA GTGCCCTGCAG (3MMs)	NM_000302 GAGCAAACCGTA GTCCCCTGGAG (4MMs)	NM_017811 GAGTTGACACTA GTGCCCAGGGG (3MMs)
Isogenic pair alignment	E99K1-Corr / E99K1	100%	100%	100%	100%	100%	100%
	NC-Edit-E99K/ NC	100%	100%	100%	100%	100%	100%

Figure S2. Analysis of potential mismatched guide sequences. (a) DNA electrophoresis gels showing the PCR amplification of six genes identified as containing the most likely potential mismatched guide sequences (3 or 4 mismatched bases). **(b)** DNA sequencing of the PCR products showed complete alignment with untargeted donor lines with no INDELs present, indicating no off-target events had occurred.

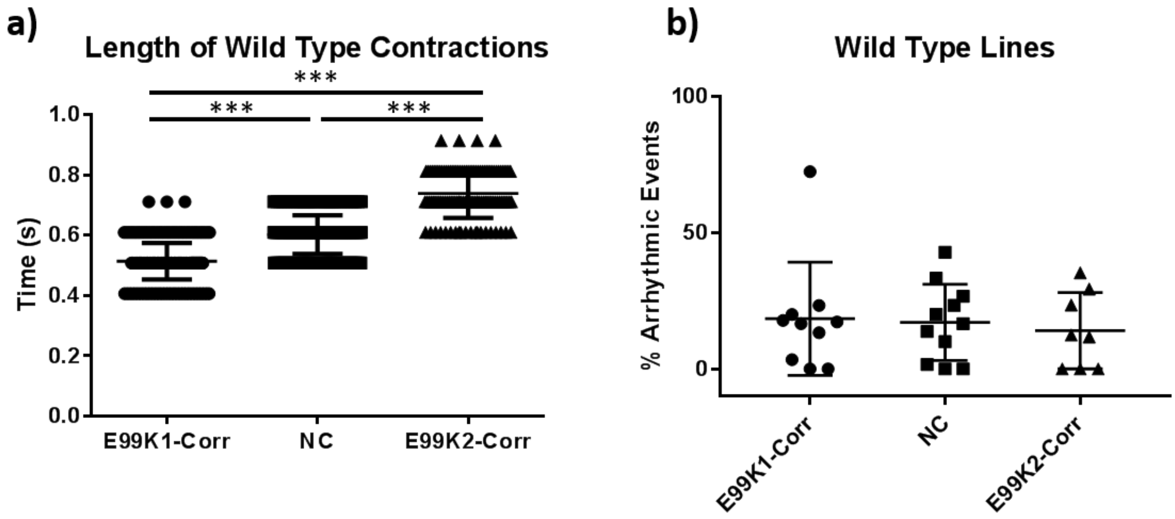

Figure S3. Wild type EHT analysis. a) Total twitch duration (combined contraction and relaxation time) was calculated calculated by pClamp software and averaged for 211 E99K1-Corr, 218 NC and 213 E99K2-Corr wild-type contractions in 7 EHTs. b) Arrhythmic event frequency in wild type 3D hiPSC-CM EHTs. Wild type EHTs were stimulated at 1Hz and arrhythmic events were counted and expressed as a percentage for E99K1 (N=10), NC (N=11), and E99K2 (N=8). All error bars represent standard error of the mean. Significance was determined by one-way ANOVA.

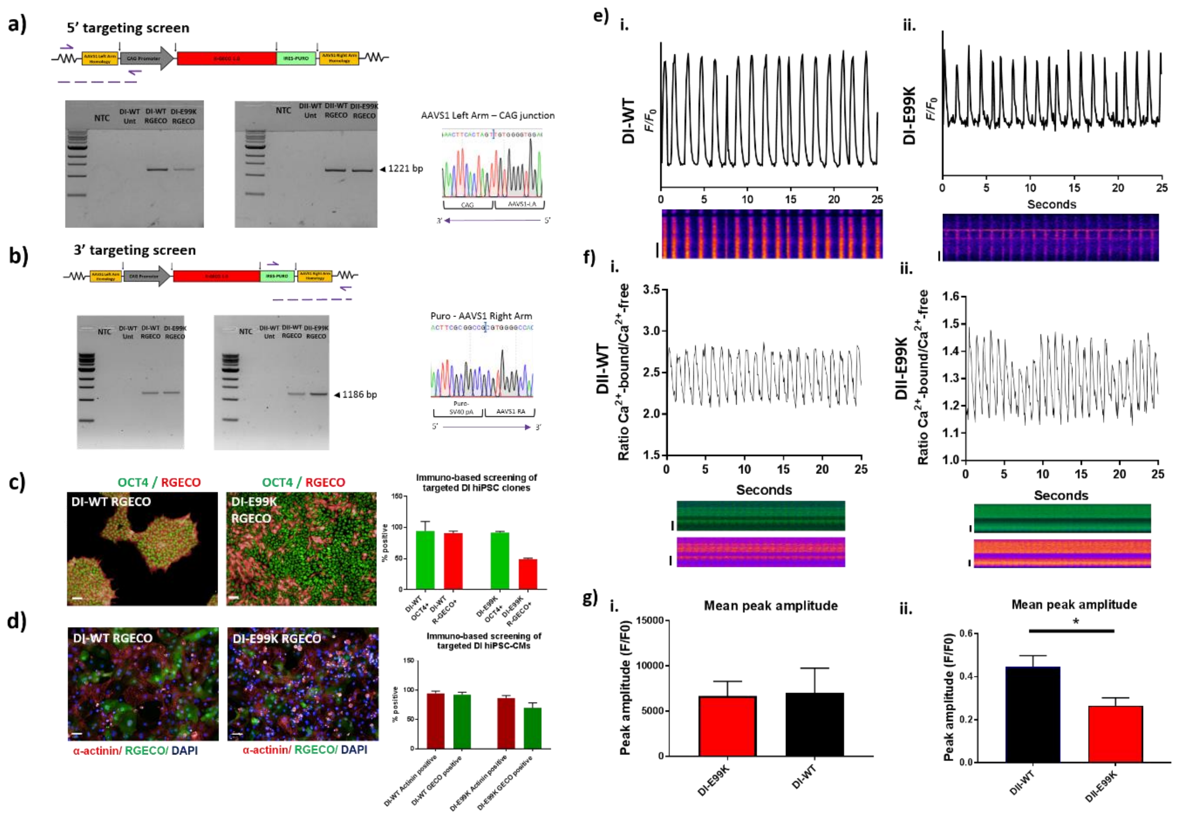

Figure S4. Characterization and arrhythmogenic event frequency of GECO-edited hiPSC lines. (a) Confirmatory 5' targeting PCR screen on gDNA isolated from E99K1 RGECO isogenic hiPSCs (left) and NC RGECO isogenic hiPSCs (right). Positive 5' targeting is indicated with a 1221bp product. These products were sequenced to show the junction between the AAVS1 left arm homology and the start of the CAG promoter. **(b)** Confirmatory 3' targeting PCR screen on gDNA isolated from from E99K1 RGECO isogenic hiPSCs (left) and NC RGECO isogenic hiPSCs (right). Positive 3' targeting is indicated with an 1186bp product. Sequencing of PCR products show the junction between the puromycin-SV40 pA sequence and the AAVS1 right arm. **(c)** E99K-Corr and E99K1 hiPSCs stained for the pluripotency marker OCT4 (green) and R-GECO (red). **(d)** E99K-Corr and E99K1 clones differentiated to hiPSC-CMs and stained for the cardiac marker α–actinin (red) and R-GECO (green). Scale bars = 50 µm. In **(e),** representative confocal line-scan traces and corresponding kymographs of spontaneous Ca^{2+} transients in E99K1 **(ei)** and E99K-Corr **(eii)** hiPSC-CMs utilising genetically encoded expression of R-GECO1.0 from the *AAVS1* locus. A line scan image was taken across a single cardiomyocyte every 75 milliseconds for 300 seconds. In **(f),** representative confocal line-scan traces and corresponding kymographs of spontaneous Ca^{2+} transients in NC **(fi)** and NC-Edit-E99K **(fii)** hiPSC-CMs utilising genetically encoded expression of ratiometric GEM-GECO from the *AAVS1* locus. **(g)** Mean peak amplitude analysis of Ca^{2+} transient events occurring during a 300 second line scan. Scale bars = 20 µm.

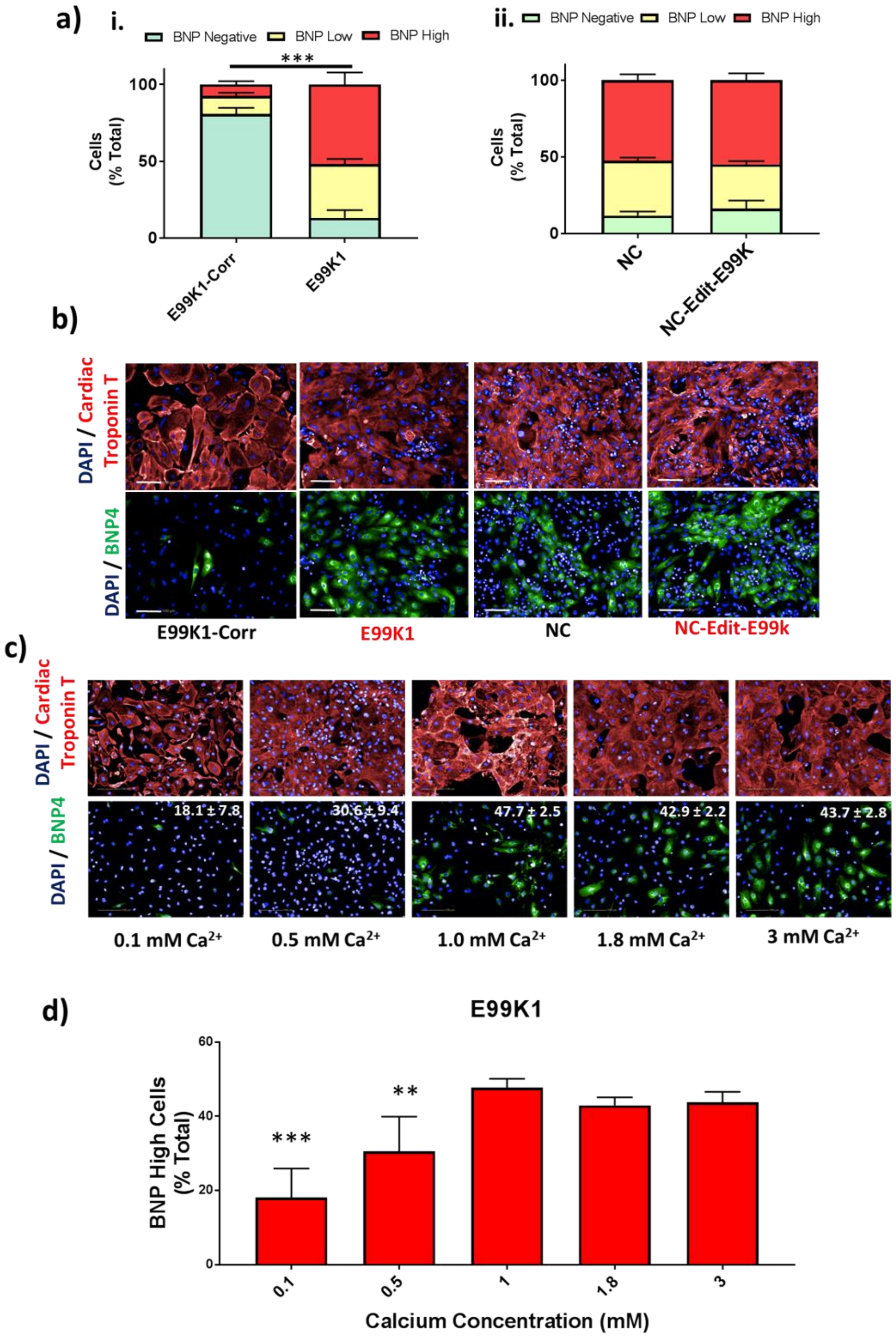

Figure S5. Hypertrophic BNP signalling in hiPSC-CMs. Quantification of BNP expression by an automated algorithm in gene-edited lines in comparison to their respective isogenic controls **(ai-ii)** from representative fluorescent micrographs **(b)** of BNP/cTnT/DAPI-immunostained hiPSC-CMs. E99K1 hiPSC-CMs were exposed to varied Ca^{2+} concentrations in Tyrode's solution for 24 hours and BNP/cTnT/DAPI-immunostained **(c)** and the percentage of highly-expressing BNP hiPSC-CMs quantified **(d)**. n = 3 E99K1, 3 E99K1-Corr, 3 NC, 3 NC-Edit-E99K. Scale bar =100 µm. n = 3. All error bars represent standard deviation. Significance was determined by students t-test, where: ** = p<0.01, *** = p<0.001.

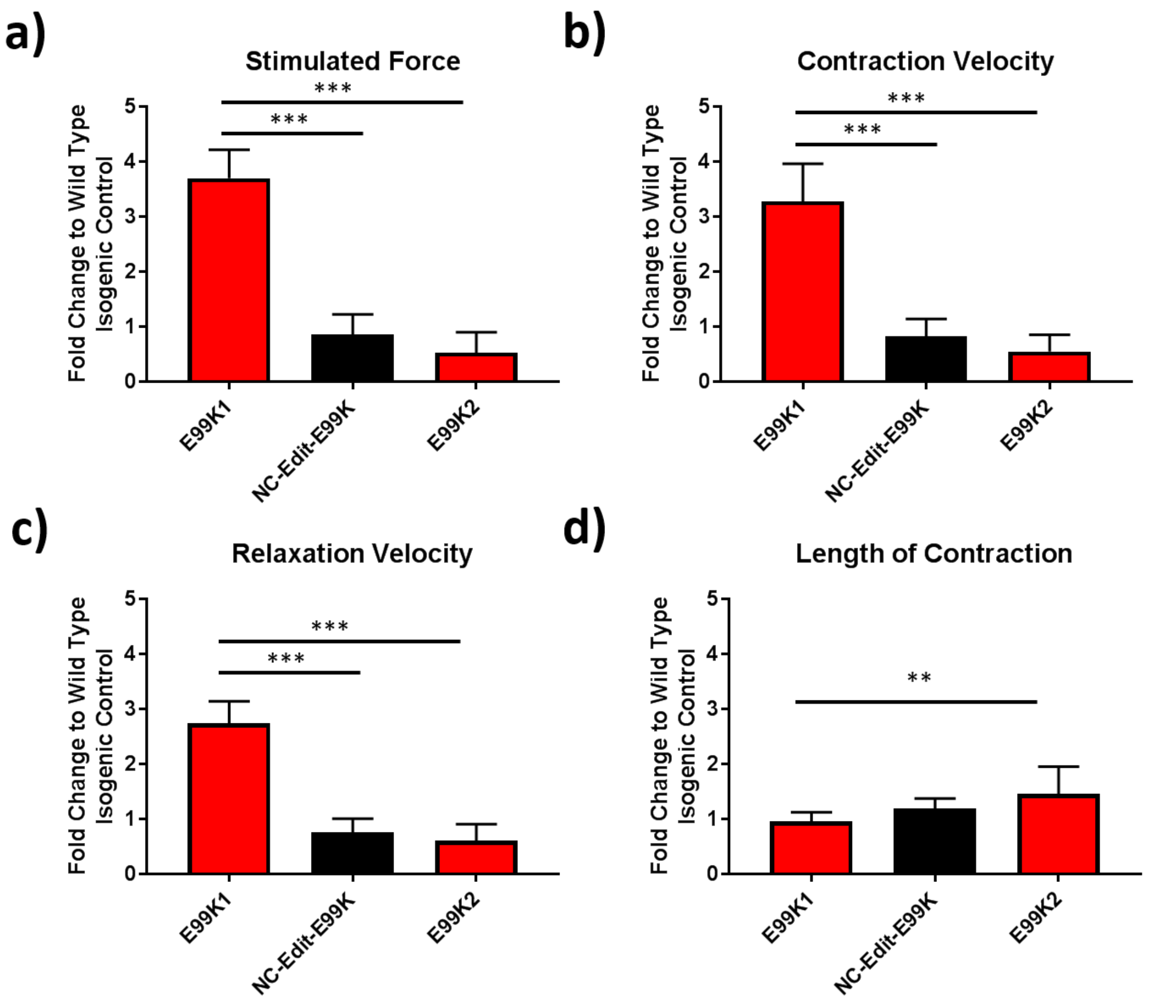

Figure S6. Normalised stimulated contraction of mutant hiPSC-CM EHTs. Auxotonic EHT contractions were recorded at 100f.p.s under stimulated conditions (1Hz). In (a) stimulated contraction force, (b) contraction velocity, (c) relaxation velocity, (d) length of contraction are shown respectively and normalised by fold change to healthy isogenic control lines. All error bars represent standard error of the mean. Significance was determined by one-way ANOVA, where: ** = $p<0.01$, and *** = $p<0.001$. n = 10 E99K1, 11 NC-Edit-E99K, 5 E99K2. Red, mutant ACTC E99K, black wild-type.

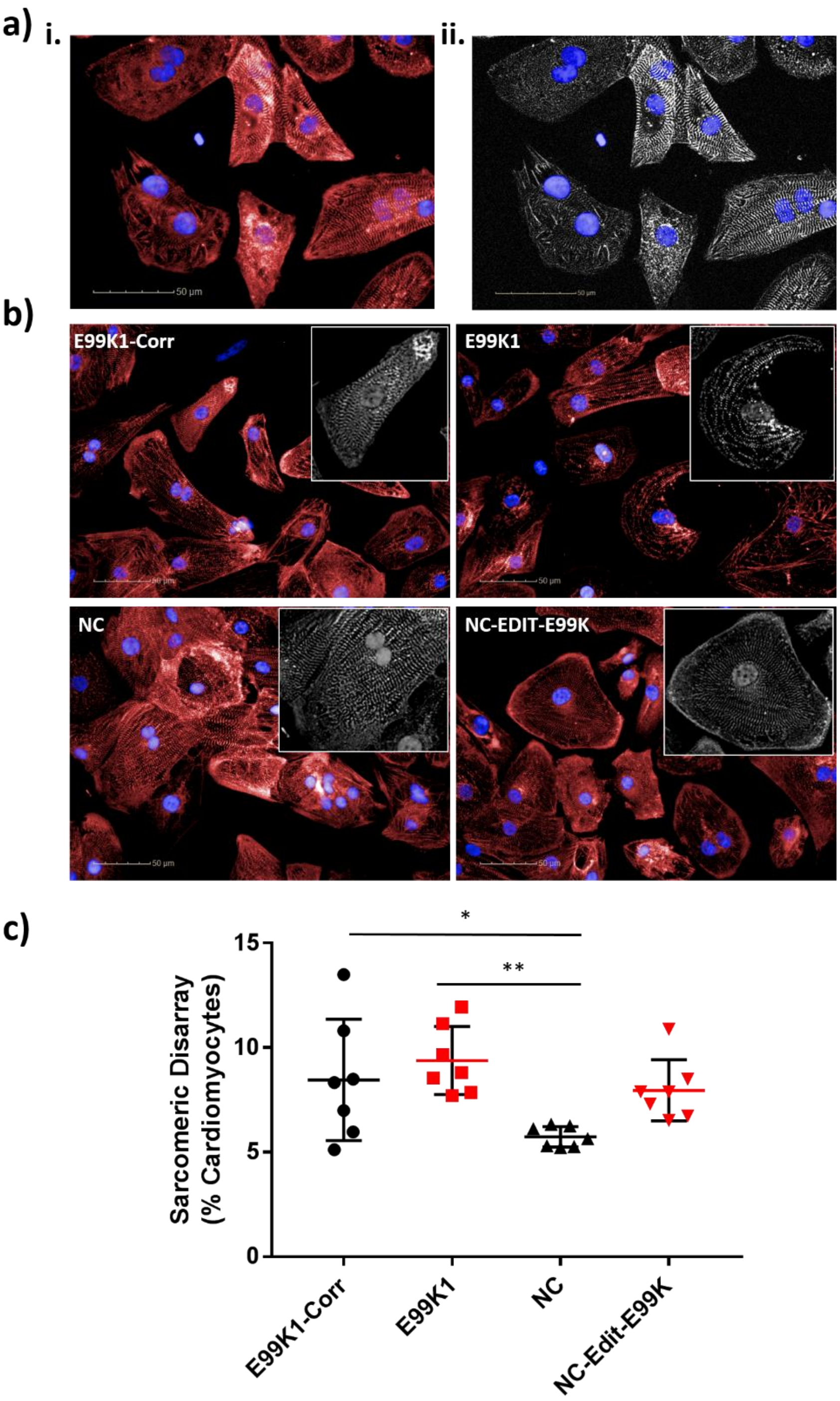

Figure S7. Sarcomeric disarray of hiPSC-cardiomyocytes. (ai) Images of human pluripotent stem cell-cardiomyocytes immunostained for sarcomeric banding (red is a-actinin staining; blue is DAPI), were sharpened using a mathematical correction (sliding parabola) to achieve higher signal resolution for alignment analysis **(aii)**. Sarcomeric disarray in isogenic pairs of hiPSC-CMs was imaged **(b)** and quantified **(c)** through analysis of morphological and texture properties in a PhenoLOGIC™ machine learning imaging algorithm (adapted from PerkinElmer). n=7. Average cells per n analyzed for E99K1 (4187), E99K1-Corr (2938), NC (3961), and NC-Edit-E99K (3072). Scale bar =50µm. Significance was determined by ANOVA, where: * = $p<0.05$, ** = $p<0.01$.

Supplementary Experimental Procedures

Fibroblast isolation

Fibroblasts were released from skin punch biopsies through manual dissection of tissue into 1mm pieces, and enzymatic digestion at 37 ^{0}C with 2.5% trypsin Trypsin-EDTA (LifeTechnologies #10462502) for 20 minutes, followed by 1 mg/ml collagenase IV (LifeTechnologies #17104019) for 90 minutes. Released fibroblasts were centrifuged at 200xg for 5 minutes, and resuspended in a fibroblast growth medium consisting of DMEM basal medium (Gibco #11965092) supplemented with 20% heat-inactivated fetal calf-serum (FCS, Biochrom #S0615), 1% Non-Essential Amino Acids (NEAA, Gibco #11140050), 1% GlutaMAX (Gibco#35050061), 100 µM β-mercaptoethanol (Sigma #63689) and 1% penicillin/ streptomycin (PEST, Gibco).

hiPSC culture

All cell culture experiments were performed in a type II Biological Safety Cabinet, and cells were incubated in a humidified incubator at 37 °C and 5% CO_2. hiPSCs were routinely maintained in E8 medium on 1:100 Matrigel (Corning #356235)-coated plastic ware (Nunc). Cells were passaged every 3 days by washing once with Ca^{2+}/ Mg^{2+}-free Phosphate Buffer Saline (PBS, Gibco #14190-094), followed by incubation with TrypLE for 6 minutes. Thereafter, hiPSC were resuspended in E8 supplemented with 10 µM Y-27632 (ROCKi, Tocris Bioscience #1254/10) and seeded into new Matrigel-coated flasks at approximately 20000 cells/ cm^2. Medium was changed every day and cell lines were used between passages 20-30.

hiPSC-CM dissociation

hiPSC-CMs generated by the monolayer differentiation method were dissociated using a Collagenase II-based protocol, as previously described (Breckwoldt et al. 2017). Briefly, cells were washed twice with Ca^{2+}/Mg^{2+}-free Hank`s Balanced Salt Solution (HBSS, LifeTechnologies #14175095). Subsequently, cardiomyocytes were incubated with 200U/ml Collagenase II (Worthington #LS004176) in Ca^{2+}/Mg^{2+}-free HBSS, supplemented with 1mM HEPES (Sigma

#H4034), 10 µM Y-27632 and 30nM N-Benzyl-p-toluenesulfonamide (BTS, TCI #B3082), for 3.5 h at 37 °C at 5% CO_2. Thereafter, dissociated cardiomyocytes were collected from the flasks and washed with RPMI supplemented with 24 µg/ml Deoxyribonuclease II (DNAseII – Sigma #D8764), followed by centrifugation at 100 xg for 15 min. Subsequently, cells were resuspended in warm RPMI and pipetted slowly through a 100 µm cell strainer (VWR # 89508-840) into a tube. Cells were then counted using an automated CEDEX HiRes counter (Roche) and centrifuged again as above. Afterwards, cardiomyocytes were resuspended in RPMI+B27+INS for seeding into Vitronectin-N (VN, Lifetech #A14700)-coated vessels.

Confocal line scans of targeted GECI hiPSC-CM Ca^{2+} transients

Targeted GECI hiPSC-CMs were generated as previously described (Mosqueira et al. 2018), cultured in RPMI+B27+INS without phenol red (Gibco #11835030) and dissociated on day 15. hiPSC-CMs were seeded at a density of 150,000 cells per well in VN-coated 35mm-diameter MatTek dishes (Nunc). CMs were assayed no earlier than day 24 and no later than day 30 of differentiation. Intracellular Ca^{2+} transient measurements were made using an LSM 880C confocal microscope (Carl Zeiss) in the line-scan mode, as previously described (Yazawa et al. 2011). Briefly, CMs were located using a 40x oil objective and a longitudinal line was drawn across a single CM. Line-scan images were taken every 75 milliseconds for a total of 4000 cycles resulting in a 5 minute scan. CMs were kept at 37 °C and 5% CO_2 throughout data acquisition. Confocal line scan images were analysed in Fiji software (National Institute of Health). The average fluorescence intensity of each line was calculated over time to generate a confocal line-scan trace. Using the multi kymograph Fiji plugin, a corresponding kymograph image was produced. In order to calculate beat rate and arrhythmic events, data was fed into pClamp software (Molecular Devices). Baselines were adjusted to account for photobleaching, and Ca^{2+} transients were counted and analysed using the 'event detection' function. In order to determine abnormal delayed after depolarization DAD-like Ca^{2+} transients, median peak height analysis was performed using Excel (Microsoft), wherein the median peak height for a line-scan image was calculated, and any Ca2+ transient events that were below 75

% of the peak height were considered ‘abnormal’, including those that did not return to baseline and gave a ‘double peak’.

BNP assay

BNP assay was performed as previously described (Carlson et al. 2013). In brief, dissociated hiPSC-CMs were seeded at 100,000 cells/ cm^2 in VN-coated 96 well plates (CellCarrier, Perkin Elmer). One week later, cells were incubated with either Tyrode’s solution with varied Ca^{2+} concentrations, or RPMI+B27+INS with or without 10 µM ranolazine (SelleckChem #1425) or dantrolene (Cayman Chemicals # 14326) for 15 h, after which 1 µg/ml Brefeldin A (Sigma #B7651) was added to the medium and incubated for another 3 h, at 37 °C and 5 % CO_2. Thereafter, cells were fixed and immunostained as described below. Image acquisition was performed as described below and BNP signal intensity was determined in the perinuclear region of cardiomyocytes, and divided into high, medium and low/negative according to pre-established empirical thresholds.

Immunocytochemistry (ICC) and image acquisition

Dissociated hiPSC-CMs or hiPSC were cultured in VN- or Matrigel-coated 96-well plates (CellCarrier, Perkin Elmer #6005550) respectively, at approximately 50,000 cells/cm^2 as described above. Cells were washed with PBS and fixed in 4% Paraformaldehyde (PFA, Sigma) at RT for 15 min. Afterwards, cells were washed in 0.1% Tween-20 (Fisher Scientific) in PBS, permeabilized with 0.1% Triton-X (Sigma) in PBS for 30 min at RT, and incubated with 4% goat serum (Sigma) in PBS (blocking solution) for 1h at RT, to prevent unspecific antibody binding. Subsequently, primary antibody incubation was performed overnight at 4 °C in blocking solution, at the following dilutions: anti-OCT4-1:200 (Santa cruz biotech #sc-5279), anti-α-actinin-1:800 (Sigma #A7811), anti-TroponinT-1:500 (Abcam #45932), anti-E99K-ACTC1: 1:800 (OriGene Technologies #AP54763PU-N), (Rowlands et al. 2017), anti-ProBNP4-1:500 (Abcam #13115). Thereafter, samples were washed 3 times with 0.1% Tween20 in PBS and incubated with Alexa Fluor secondary antibodies (Life Technologies) 1:400 in blocking solution for 1h at RT. Afterwards, cells were washed with 0.1% Tween-20 in PBS for (3x 5min), followed by nuclei counterstaining with 0.5

μg/ml DAPI (Sigma #D9542) in PBS, respectively, for 15 min at RT. Samples were subsequently washed and stored at 4 °C in PBS until automated image acquisition was performed in the Operetta high-content imaging system (Perkin Elmer) and analysed using Harmony high-content imaging analysis software.

CellOPTIQ analysis

The CellOPTIQ platform (Hortigon-Vinagre et al. 2016) was used to record optical-based Ca^{2+} transients from hiPSC-CMs. Cells were seeded into Matrigel-coated 96-well plates at a density of 50,000 cells per well. These hiPSC-CMs were then incubated at 37 °C and 5% CO_2 for 48 h to allow cells to recover. To image the Ca^{2+} handling properties of the hiPSC-CMs, they were loaded with Fluo4-AM (LifeTechnologies #F14201). Cells were incubated in RPMI media containing 10 μM Fluo4-AM at 37°C and 5% CO_2 for 30 min. After the 30-min incubation, the medium was removed and the hiPSC-CMs washed once before 100 μL of fresh medium was added to wells. These plates were then incubated at 37 °C and 5% CO_2 for 15 min to allow the hiPSC-CMs to equilibrate before recording traces. Data were analysed using CellOPTIQ proprietary software of Clyde Biosciences and were normalized to a maximum amplitude of 1 and minimum of 0 to standardize height for comparison of traces created in Origin software package.

Real-time qPCR

RNA was extracted using the RNeasy mini kit (Qiagen), and cDNA synthesis performed using Superscript III (Life Technologies) following manufacturer's instructions. Real-time qPCR reactions were performed using TaqMan® Gene Expression Assays (Applied Biosystems) following manufacturer's instructions. Briefly, reactions were performed in MicroAmp Fast 96 well plates (#4346907) and contained 2x Taqman® gene expression mastermix (#4369016) and the relevant TaqMan® gene expression assay (CASQ2-Hs00154286_m1, CALM1-Hs00300085_s1, CAMK2D-Hs00943538_m1, PPP3cA-Hs00174223_m1, PPP3cB-Hs00236113_m1, IRF8-Hs00175238_m1, PLN-Hs01848144_s1, CAPN1-Hs00559804_m1, CACNA1C-Hs00167681_m1). Amplification was performed in ABI 7500 Real-Time PCR system (Applied Biosystems). Normalisation was performed

using the cardiac gene TNNT2 and 18S as reference genes, as previously described (Burkart et al. 2016), and the WT isogenic cardiomyocytes lines were used to calculate relative expression using the 2-(ΔΔCT) method (Schmittgen and Livak 2008).
